# A satellite based machine learning approach for estimating high resolution daily average air temperature in a megacity in Brazil

**DOI:** 10.1038/s41598-026-35689-x

**Published:** 2026-02-05

**Authors:** Aina Roca-Barceló, Rochelle Schneider, Monica Pirani, Alessandro Sebastianelli, Frédéric B. Piel, Paolo Vineis, Adelaide Cassia Nardocci, Daniela Fecht

**Affiliations:** 1 MRC Centre for Environment and Health, Department of Epidemiology and Biostatistics, School of Public Health, Imperial College London, 90 Wood Ln, London, W12 0BZ UK; 2 Φ-lab, European Space Agency (ESA), Frascati, Italy; 3 Forecast Department, European Centre for Medium-Range Weather Forecast (ECMWF), Reading, UK; 4 Faculty of Epidemiology and Population Health, London School of Hygiene and Tropical Medicine (LSHTM), London, UK; 5 CMCC Foundation - Euro-Mediterranean Center on Climate Change, Caserta, Italy; 6 UK Small Area Health Statistics Unit, Department of Epidemiology and Biostatistics, School of Public Health, Imperial College London, London, UK; 7 Department of Environmental Health, School of Public Health, University of São Paulo, São Paulo, Brazil

**Keywords:** Random forest, Ambient temperature, Remote sensing, Spatial cross-validation, Forward feature selection, Climate sciences, Ecology, Ecology, Engineering, Environmental sciences, Mathematics and computing

## Abstract

Spatiotemporally resolved ambient temperature data are essential for environmental epidemiology, especially in urban areas where temperature can vary sharply over short distances, influencing population exposure. Additionally, heat distribution often reflects built environment patterns and may correlate with existing social and environmental disparities. Continuous temporal records at high spatial resolution are, however, often lacking, especially in low- and middle-income countries. We developed a generalizable tree-based machine learning approach to estimate daily mean temperatures at 500 × 500 m resolution using São Paulo, a megacity in Brazil, as a case study, to demonstrate its utility in highly urbanized settings with a heterogeneous urban fabric and unevenly distributed temperature monitoring stations. We trained a Random Forest model using open-access remote sensing data, along with derived products, and temperature measurements from 43 ground stations. To prevent overfitting and select relevant features, we employed a forward feature selection algorithm with target-oriented (spatial) cross-validation. Hyperparameter tuning was performed using grid search approach. The model was validated through ten-fold station-based cross-validation and an external hold-out dataset. The model demonstrated strong performance (RMSE_RF_ = 0.80; R^2^_RF_ = 0.95), with slightly reduced accuracy in rural areas (R^2^_rural_ = 0.91; R^2^_urban_ = 0.95). Compared to traditional multilinear approaches (RMSE_MLR_ = 1.02; R^2^_MLR_ = 0.92), the Random Forest model outperformed, likely due to its ability to better capture microclimates and complex relationships between data sources. This 500 × 500 m daily temperature dataset is the first of its kind in South America, with the São Paulo pipeline and data freely accessible. The approach is adaptable to other regions with appropriate retraining and validation, enabling high-resolution exposure assessments.

**Supplementary Information:**

The online version contains supplementary material available at 10.1038/s41598-026-35689-x.

## Introduction

Non-optimal temperatures are known to have significant adverse health effects. Most of this evidence comes from time-series studies that link citywide daily ambient temperature (*Ta*) to daily mortality or hospital admissions^1–6^. These studies often use a temperature summary over the entire city extent or a unique point measurement to represent the city, thus, assuming a uniform temperature distribution across cities. This approach overlooks local variations and underestimates the impacts of temperature difference within cities. This is largely due to the lack of high-resolution *Ta* data, particularly in low- and middle-income countries (LMIC)^5,7^. Consequently, the relationship between temperature and health outcomes within cities, including vulnerabilities among specific populations and neighbourhoods, remains poorly understood. This gap is particularly concerning as over half of the global population currently resides in urban areas, a figure projected to rise to 68% by 2050, increasing population exposure to urban temperature^8^.

While gridded global *Ta* products exist, most lack either the temporal or spatial resolution necessary for epidemiological studies concerning urban settings, which typically require daily or weekly data at spatial resolutions finer than 1 km^2^. The fifth generation European Centre for Medium-Range Weather Forecasts (ECMWF) atmospheric reanalysis products, ERA5 (31 × 31 km) and ERA5-Land (9 × 9 km), are Copernicus reanalysis products, which integrate numerical simulations with historical data to provide consistent global hourly *Ta* estimate. Masselot and colleagues^9,10^ used these Copernicus reanalysis products and demonstrated their ability to replicate citywide temperature-mortality relationships similar to those derived from station-based data. However, their spatial resolutions are too coarse to capture temperature variations within city environments accurately. On the other hand, the Global Seamless High-resolution Temperature Dataset (GSHTD) offers a much finer spatial resolution yet remains limited to monthly averages^11^.

Urbanization is one of the most significant and transformative forms of land conversion, typically involving the shift from natural or agricultural land to urban areas dominated by impervious surfaces. These areas are often characterized by high building and population densities. While urbanization can take various forms, its influence on local environmental and climatic conditions, particularly temperature, is well documented^12–14^. Studies have shown that the spatial and temporal distribution of temperature evolves as cities grow^15^, with microclimates of localized heat and cold islands dynamically forming and vanishing within cities as they change^16^. Thus, ignoring the spatial and temporal variability of temperature within cities can introduce inaccuracies or even biases in epidemiological and climate studies^17^.

To address these challenges, recent research has increasingly focused on using statistical models to estimate *Ta*. As opposed to physical and numerical models, which are complex and computationally intensive, requiring highly qualified operators, and large amounts of resources and time; statistical models provide a simpler, scalable and less computationally demanding solution. These models can generate high-resolution, spatiotemporally detailed temperature estimates by combining temperature data from ground measurements with predictor variables, often gridded data with high spatial and temporal resolution, such as land surface temperature (LST), a common approach for other exposures like air pollution^18,19^. To enhance model accuracy, additional covariates such as vegetation indices, water body indices, and population density are often incorporated to account for variations not captured by LST alone. However, most of these products are either born from academic interest such as those for London^20^, Serbia^21^, or the recently published dataset for Peru^22^, or only available in regions with the necessary infrastructure to support their development and sustainability, often located in the Northern Hemisphere^23^. Thus, there is a pressing need for better coverage of the Southern Hemisphere, particularly in rapidly urbanizing regions^7,24,25^, such is the case of São Paulo, Brazil. São Paulo is Brazil’s largest metropolitan area with over 22 million residents. It represents 10% of the country’s population and is projected to reach nearly 24 million by 2030^8^. Over the years, there has been an increase in the city’s mean annual temperature, which has been primarily linked to both climate change and the rapid urban growth experienced by the area in the last 30 years^15^.

Here, we developed a modelling framework, based on an ensemble learning Random Forest (RF) algorithm, to produce a high spatial resolution daily ambient mean temperature dataset for São Paulo. Both the dataset and the modelling framework are openly accessible. The modelling framework is generalizable and easily replicable (with the appropriate retraining and validation) to other periods and locations, providing a resource-efficient approach to expand and refine local climate data and insights.

## Methods

We estimated daily *Ta* across São Paulo, Brazil at a 500 × 500 m spatial resolution for 5 years (2015 to 2019) using a random forest regression (RF) model. The model was trained on 43 ground monitoring stations and 8 predictor variables selected through forward feature selection (FFS) with station-based cross-validation (CV). In detail, we tested the robustness of the model using station-based CV and external validation using 5 hold-out stations. We also compared our model to a traditional multi-linear regression model. Figure S1 depicts the modelling approach. All data processing and handling of temperature and predictor variables were performed using R software (version 4.1.3). Random Forest model training and validation were conducted in Python (version 3.10) on Google Collab using the scikit-learn package.

### Study area

**Fig. 1:**
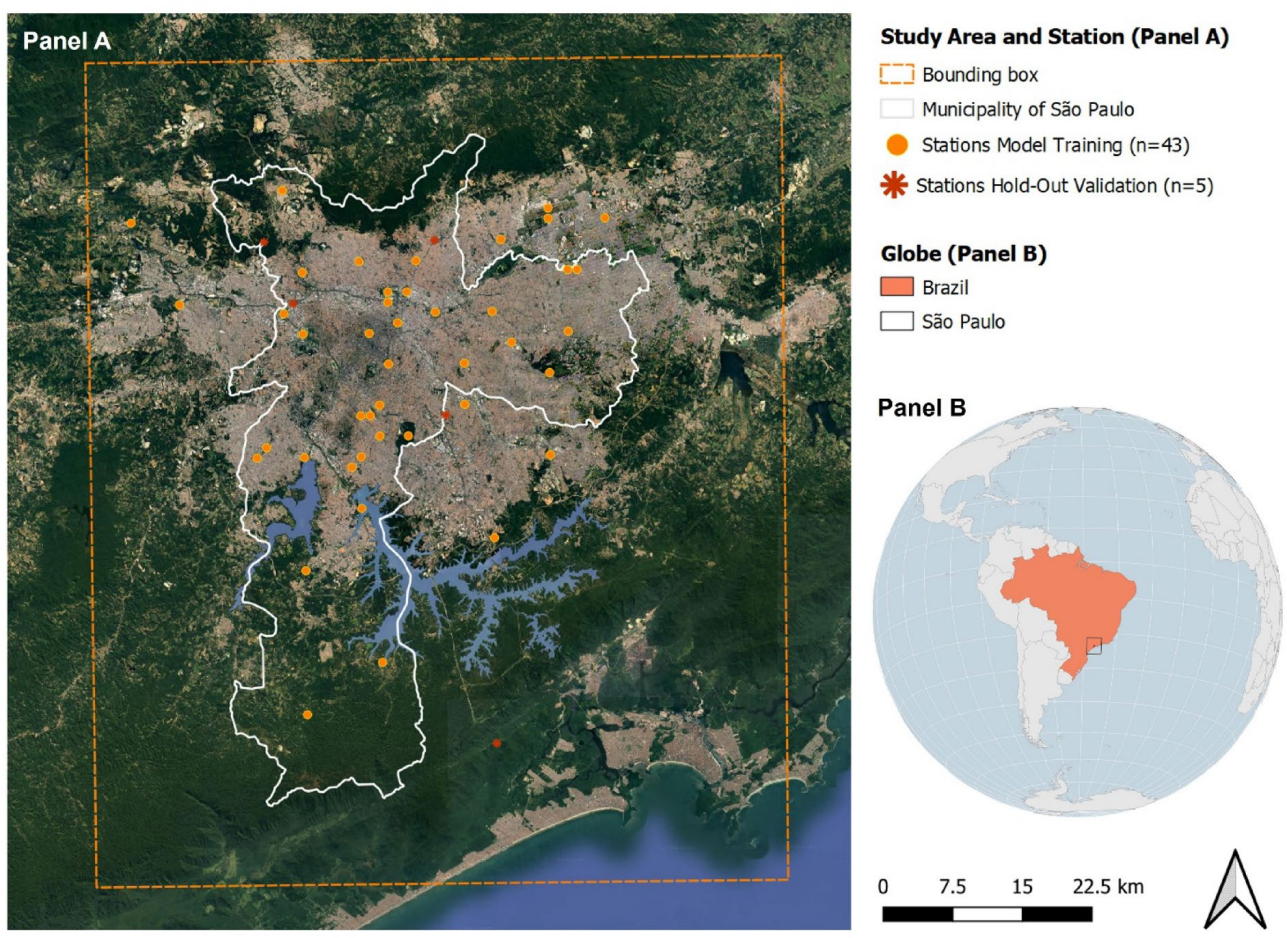
Panel (**A**), study area including Municipality of São Paulo (white) and delimited by the bounding box (dashed orange line). Overlayed, the meteorological stations used for model training (orange dots, *n* = 43) and those set aside for the external or hold-out validation (red asterisk, *n* = 5). Panel (**B**), location of the study area of São Paulo (black bounding box) within Brazil (orange shade) in South America.

The study area was delineated to cover the municipality of São Paulo, which contains the megacity of São Paulo. With over 11 million inhabitants over an area of 1521.1 km^2^, the municipality of São Paulo is the largest urban agglomeration and most populous urban area in Brazil^8^. It has a varied urban fabric with a large spatial heterogeneity in the building type, building density and layout, deprivation, distribution of green and blue spaces and population density. The São Paulo municipality can be divided into humid subtropical climate in the north and temperate oceanic climate in the south which receives influence from the ocean breeze^26^. The modelling domain was defined by the envelope of São Paulo municipality administrative boundary extended to include additional monitoring stations (lon_min_: -46.9559; lat_min_: -24.0854; lon_max_: -46.2226; lat_max_: -23.2839, coordinate reference system: WGS84) (Fig. 1 Panel A). The study area covered 6,213km^2^ and consisted of 24,853 grid cells with a 500 × 500 m resolution.

### Ambient daily temperature

We collected daily mean temperature data from 55 stations operating at least one year between 2015 and 2019 from seven monitoring networks. Duplicated stations (*n* = 4) were identified, and those with the most complete series retained. Stations with less than one year of data were excluded (*n* = 5). The final dataset comprised 48 ground stations from seven networks (Fig. 1 Panel A/B, Figure S2) and included 78,569 observations after removing 10% missing values (9,079 days out of 87,648). Further details regarding the selected temperature indicator and the data processing steps are provided in Supplementary Materials S1 and 2.

### Spatiotemporal predictor variables

Spatiotemporal predictor variables were chosen based on their association with daily mean temperature distribution and their free, global availability for model transferability. The latter condition was to allow for future transferability of the model to other locations. The workflow for processing predictor variables is shown in Figure S3. We analysed 23 candidate predictor variables, numbered [1-23] in the text (Supplementary Material Table S1 for more information).

**Atmospheric variables**:^1^ Land Surface Temperature (LST) is the thermal radiance emitted by Earth’s surface due to solar radiation, influencing surface energy balance and evapotranspiration, and thus temperature. LST data were derived from the LST gap-filled algorithm developed by Shiff and colleagues^27^ which uses level 3 MODIS LST^28^ product (MYD11A1 Version 6) from the Aqua polar-orbiting NASA sun-synchronous satellite (1:30 AM/PM local time) combined with surface air temperature derived from the NCEP CFSv2 model^29^. This product, while having a coarser spatial resolution (1 × 1km) than other remote sensing products (e.g., Landsat), offer daily temporal resolution and no missing values. LST is the thermal radiance emitted by Earth’s surface due to solar radiation, influencing surface energy balance and evapotranspiration, and thus temperature^2^. Solar Zenith Angle (SZA) is the angle between the local zenith and the line of sight to the sun, and it has been shown to be a strong predictor of temperature^20,30,31^. We used the 16-day SZA band from the “MODIS/Terra Vegetation Indices 16-Day L3 Global 1km SIN Grid” USGS data product^32^^3^. Black Sky Albedo (*BSA*) or directional hemispherical reflectance, measures the reflectance ratio using only the direct component of incoming radiation, unlike white-sky albedo, which considers only diffuse radiation. The scale ranges from 0 (total absorption) to 1 (total reflection) and tends to be higher in urban and industrial areas, while lower over water and greenspaces. Describing energy flux at the land boundary layer, surface albedo influences regional and global climate patterns, making it an interesting parameter to include in temperature modelling^33^. We used the shortwave band of the MODIS Terra and Aqua Bidirectional Reflectance Distribution Function and Albedo Version 6.1.’ data product (MCD43A3v061)^34^. It provides both black and white-sky albedo data for every day at local solar noon at 500 × 500 m spatial resolution. It uses 16-days of Terra and Aqua MODIS data temporally weighted to the ninth day of the 16-day window.

#### Weather data (ERA5-Land)

We extracted several atmospheric variables^35^available hourly at a 9 × 9 km spatial resolution, from the ECMWF Re-Analysis 5th generation Land (ERA5-Land)^35^. The^4^ 2 m air temperature (*t2m*) represents *Ta* at a height of two metres above the Earth’s surface, while the^5^ 2 m dew point temperature (*d2m*) indicates the temperature at which air would saturate at the same height^6^. Relative humidity (*rh*) was calculated from *t2m* and *d2m* using Wright’s formula (1997). The^7^ skin temperature (*skt*) is the theoretical temperature required to satisfy the surface energy balance. The^8^ surface pressure (*sp*) reflects the pressure of the atmosphere at an area of the Earth’s surface, measured as the height of air in a vertical column in force per unit area in Pascals, Pa. Finally, the^9^ 10 m eastward (*v10*) and^10^ northward wind component (*u10*) measure horizontal air speed moving North and East, respectively, at a height of ten meters above the Earth’s surface.

#### Topography

São Paulo includes mountainous areas and thus, elevation and slope were important variables to consider. For^11^ elevation, we used the ‘NASA SRTM Digital Elevation model version-3 product‘^36^ part of the NASA Shuttle Radar Topography Mission (SRTM)^37^ at 500 × 500 metres resolution. We derived^12^ slope (*slope)* from the DEM dataset using the GEE function *ee.Terrain.slope()*, which determines elevation change based on each pixel’s four-connected neighbourhoods. All topography data were downloaded and processed in the GEE cloud-based platform^38^ using the Python API.

#### Land use variables

We used 500 × 500 m Normalized Difference Vegetation Index (*NDVI*) data from the ‘USGS Landsat 7 Collection 1 Tier 1 calibrated Reflectance’ courtesy of the U.S. Geological Survey [255]. NDVI measures vegetation health by comparing the reflectance of visible and near-infrared light. Negative values indicate clouds or water, values near zero indicate bare soil, values between 0.1 and 0.5 correspond to sparse vegetation, and values above 0.5 indicate dense vegetation. High NDVI values (0.6–0.9) indicate healthy, dense vegetation, while lower values (0.2–0.5) reflect sparse or unhealthy vegetation. Daily NDVI data was downloaded and processed in the GEE. Daily estimates were largely affected by cloud cover which led us to use 3-months averages to capture seasonal variation.

**Fig. 2:**
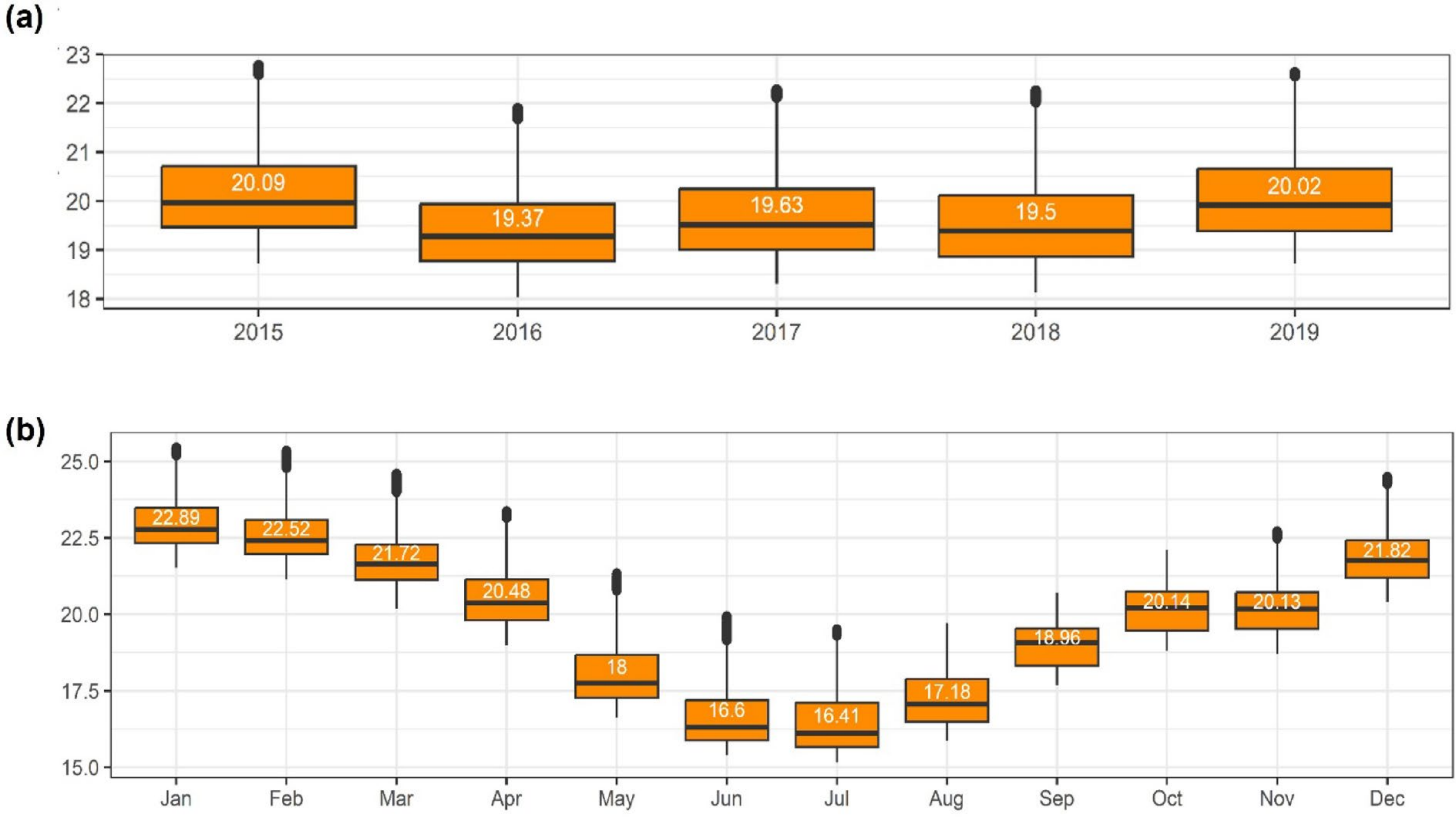
Boxplot of the predicted daily mean temperature by (**a**) year and (**b**) month, as predicted by the RF algorithm. Temperature in degrees Celsius (°C).

São Paulo is situated near the coast and within the Tietê River Basin of the Paraná Hydrographic Region, which includes three major reservoirs: Guarapiranga, Rio Pedras, and Billings. Thus, it was important to consider both inland and coastal blue spaces. For each reservoir, we obtained the maximum water extent layer by the *Alto Tietê* data platform^39^ and created^14^ 200 m (*b200m*) and^15^ 400 m distance buffers (*b400m*) to the dam/lake^16^ perimeter (*lake*) (Figure S4a-b). The *Rio Pedras* and *Billings* dams were considered together as they are physically joined. We overlaid these buffers onto a 500 × 500 m target grid. For each grid cell, we estimated the percentage of area intersecting each buffer (Fig. 2). We also explored the use of a global gridded dataset (~ 100 × 100 m) developed by WorldPop, which captures^17^ the distance (in kilometres) to the nearest inland water body (*water*) as defined by the ESA-CCI-LC water bodies classes, which contain both large and small water bodies^36^. Finally, we assessed the effect of coastal blue spaces using the R package *rnaturalearth* to calculate the^18^ distance from each grid cell centroid to the coastline (*coast*).

^19^ Population density (*popdens*) (~ 1 × 1 km) was obtained from WorldPop^36^, which produces high-resolution global population estimates by downscaling census data using Random Forest machine learning methods using two approaches: the constrained method, which limits data to known settlements, and the unconstrained method, which does not assume full settlement accuracy, thereby capturing areas where settlements may be unrecorded. To include informal settlements, common in São Paulo and often missed in regular datasets, we used the unconstrained approach for a fuller spatial distribution. This dataset has annual resolution, covering 2000–2020, reporting density as people per square kilometre.

To complement the population density data, we also utilized an^20^ impervious surface dataset (*impsurf*) developed by Zhang and colleagues^40,41^. This global dataset, available at 30 × 30 m resolution for 2015, uses a RF Classifier to identify impervious surfaces (binary). With accuracy of 95.1%, this dataset outperforms other available impervious surface maps. Finally, we used a^21^ land cover (*landcov*) dataset (~ 300 × 300 m) from the European Space Agency (ESA) Climate Change Initiative (CCI) Land Cover 2015 annual product (v2.0.7), and a^22^ distance-to-artificial-land cover (*artland*) dataset (~ 100 × 100 m) from WorldPop^36^, estimated as the geodesic distance from each grid centroid to the edges of the re-classified ESA-CCI-LC classes 2015 artificial surface layer available.

#### Temporal predictor variables

We included^23^ *daylength* to capture temporal and seasonal variations, as done in previously^42,43^.

#### Missing data and spatiotemporal harmonization

Since atmospheric and NDVI variables rely on optical sensors, they required additional processing to address cloud cover, which introduced missing values. For LST, we applied the gap-filling algorithm validated by Shiff and colleagues^27^. For variables without a specific algorithm, we used the gap-fill imputation method proposed by^44^ (Supplementary Material S3). We verified that all predictor variables met expected ranges, covered the designated area and timeframe, and showed expected spatial and temporal variability.

All predictor variables were rescaled to the target spatial resolution (500 × 500 m) using nearest neighbourhood or bilinear interpolation for continuous variables, selecting for each predictor the method that preserved spatial variability while avoiding excessive smoothing of critical extreme values crucial for model training. To harmonize the temporal component to daily average, we performed a cell-wise linear interpolation using each predictor’s raster stack across the study period. The number of available time slices varied based on the native temporal resolution of each dataset (see Table S1). The result was a raster stack of daily data in a 500 × 500 m regular grid for each of our 23 predictor variables. See Supplementary Material section S4 for more detail.

### Statistical methods

After processing the temperature and predictor data, we defined the training, test, and validation datasets (Figure S4 and S5), before training the model, generating predictions, and performing validations. We constructed the training dataset by overlaying monitoring stations onto the raster stack of daily predictor variables and extracting the intersecting cell values. The resulting dataset included station location, recording date, temperature measurement, and the values of intersecting predictor variables for each day. Days with missing temperature values were excluded (*n* = 9,079, resulting in 78,569). The dataset was then partitioned into a training and test set, and an external validation set. The external validation set was designed to assess the external validity of the model predictions, simulating performance at unsampled locations. It comprised 10% of the stations (*n* = 5), selected through random sampling. To identify these stations, random subsets were drawn and their temperature distributions compared with those of the remaining data using a two-sample *t*-test. The first subset with a *p*-value < 0.05 was chosen, indicating a statistically different but related distribution. This approach provided a stricter and more realistic evaluation of the model’s generalization ability. The external validation set contained 6,799 observations across five stations and was held completely separate from the training and test data for independent validation. This dataset was held separate from the training and test set and used solely for external model validation. The training and test set was composed of the remaining 43 stations, totalling 71,770 observations.

Random Forests (RFs)^45^ are ensemble machine learning which utilize multiple decision trees generated from bootstrap samples of the original dataset for classification and regression tasks. Decision trees are trained independently, with each sample drawn from the dataset being independent and possessing a similar distribution. In regression tasks, tree predictions are aggregated using an arithmetic mean. Feature randomness reduces tree correlation and enhances diversity in splits. The algorithm estimates information gain or loss using a loss function, commonly Root Mean Square Error (RMSE) (Supplementary Material S5 for notation). The branch with the lowest RMSE at each decision tree node is prioritized, ensuring optimal decision-making during training. RFs handle non-linearity and complex interactions while managing correlated predictors without compromising performance. Unlike kriging and geospatial interpolation methods, RFs do not require rigid statistical assumptions about variable distribution or stationarity. They are flexible with predictors and less reliant on the spatial density of meteorological stations, making them superior for prediction in data-sparse areas^46,47^. RFs have been successfully utilized in prior studies for predicting environmental variables, including temperature^20,23,48^.

#### Model training: hyper-parameter tuning and feature selection

The performance of RFs is influenced by the number of trees to grow (*n_estimators*), the maximum number of features considered at each split (*max_features*), and the maximum depth of the tree(*max_depth*). Tuning these parameters, known as hyperparameter tuning, is crucial for optimizing RF performance. Moreover, in spatiotemporal modelling, overfitting can arise from the inclusion of temporally or spatially static variables with limited variability. While the presence of numerous trees in RFs helps mitigate overfitting, removing uninformative features further reduces this risk while enhancing interpretability and generalization. To address this challenge, Meyer and colleagues^49^ proposed the Forward Feature Selection (FFS) method. FFS iteratively selects features based on performance improvement, minimizing the loss function (e.g., RMSE) through ten-fold station-based CV. Unlike traditional approaches, FFS directly evaluates model performance rather than relying on feature importance scores, outperforming traditional approaches and reducing risk of overfitting and bias^49^. We implemented the FFS algorithm using the *SequentialFeatureSelector()* in Python *Scikit-learn*^50^.

Feature selection and hyper-parameter tuning were performed simultaneously. Exploring all combinations of hyperparameters is computationally impractical; thus, we tested a subset of combinations (*n_estimators:300*,*500*,*700*,*1000; max_features: ‘auto’*,* ‘sqrt’*, and *max_depth: 10*,* 15*,* 20;* Table S2). Using a grid search approach, we iteratively ran the FFS algorithm for each hyper-parameter combination and assessed the model performance. We used a 50% random sample (n = 35,885) stratified by daily mean temperature groups and representative of all datasets (*t*-test=-0.189, *p*-value = 0.8501) to minimize computational costs whilst ensuring representativeness (Figure S6). The feature and hyperparameter combination that resulted in the lowest RMSE value obtained through a ten-fold station-based CV was selected.

We employed a permutation-based feature importance algorithm, or mean decrease accuracy score, to assesses the predictor’s importance at estimating daily mean temperature at unknown spaces/times. A baseline model is first fitted to a set of stations which are hold out from the training. The algorithm then randomly re-shuffles the values from one of the predictors in the hold-out dataset, passes the dataset to the model to obtain predictions, and calculates the performance. Feature importance is determined as the average difference between the baseline and the modified scored after re-shuffling. To ensure stability, we conducted ten iterations and report their average. This approach is less prone to overfitting compared to other feature importance algorithms, such as the Gini importance algorithm, which rely solely on the training dataset and favours continuous variables^45,51^.

#### Model validation

We evaluated the model using two methods: a ten-fold station-based CV and an external validation approach. CV involves dividing the data into training and validation sets, with multiple iterations or folds to ensure each data point is validated. The behaviour and performance are highly sensitive to the cut-offs used to define the folds. As the objective of this model was to predict temperatures at unsampled locations, assessing performance and accuracy in predicting unseen locations is of particular interest. Thus, we used a ten-fold station-based CV approach^49^, which iteratively splits the data in ten groups of stations to test the performance of the model. All model validations were conducted using the daily estimates of air temperature. For the purposes of analysis, these daily validation results were subsequently aggregated to monthly and annual temporal scales, as well as to different spatial scales (urban vs. rural), to examine whether model performance varied across time and location’s characteristics.

External validation involves the use of a hold-out dataset to investigate the model’s ability to generalize across unseen locations and time. This is particularly important to ensure the model is generalizable within the prediction domain and to mitigate the risk of overfitting. We used a hold-out dataset comprising 10% of all available stations (*n* = 5 stations; 6799 observations), reserved from the outset. Performance statistics for both included the RMSE and the coefficient of determination (R^2^). Full algebraic expressions are provided in Supplementary Material S5.

Finally, following Kloog et al. 2014’s approach, we calculated the temporal and spatial error associated with the model, measured through regression^52^. The *temporal error* was calculated by regressing the difference between the observed temperature at time *t* and space *s*, and the annual mean temperature observed, against the difference between the predicted temperature at time *i* and space *j*, and the annual mean temperature predicted. The *spatial error* was calculated by regressing the station-specific annual mean estimates in observed temperatures against the station-specific annual means from the predicted temperature. Full algebraic expressions are provided in Supplementary Material S5.

### Sensitivity analyses and model comparisons

To assess the robustness of the model to changes in the predictor variables, we tested the other top-3 best fitting feature combinations. For each combination, we estimated RMSE and R^2^ of the ten-fold station-based CV and external validation. Finally, to quantify the added value of using a RF over other simpler popular methods, we compared our model to a multi-linear regression (MLR), widely employed to predict spatiotemporal environmental variables. The features used were identical to those selected for the RF approach, ensuring comparability. We assessed model assumptions and outlier influence by examining a histogram of regression standardized residuals, a normal Q-Q plot, and a scatter plot of residuals against fitted values (Figure S5.1). Detailed formulation, model checks and outputs are presented in Supplementary Material S5. The spatial distribution of the predictions was compared by mapping average daily mean temperatures throughout the study period, analysing yearly and monthly variations, and calculating delta temperature (∆Temp) as the difference between RF model predictions (reference) and the MLR model. Pixel-level temperature correlation and ∆Temp distribution histograms were plotted for the same temporal resolutions. To compare the temporal dimension, we examined the annual and monthly predictions using box plots and chi-square test. Lastly, we compared the RMSE and R^2^ scores resulting from ten-fold CV and external validation for both models.

## Results

### Meteorological station and Spatiotemporal predictors data

The final dataset comprised 48 ground monitoring stations, of which 43 were used for training and five were set aside for the external validation. The stations mostly concentrated in higher latitudes and around the city centre where most population resides (Fig. 1). The number of valid stations increased over time, with the highest coverage observed between 2015 and 2019 (Supplementary Material S2, Figure S2.1). Missing values were spread randomly across time, monitoring networks, spatial closeness, and area-level characteristics (i.e. urban/rural and deprivation), indicating no systematic bias (Supplementary Material S2, Figures S2.1-2.9).

**Fig. 3:**
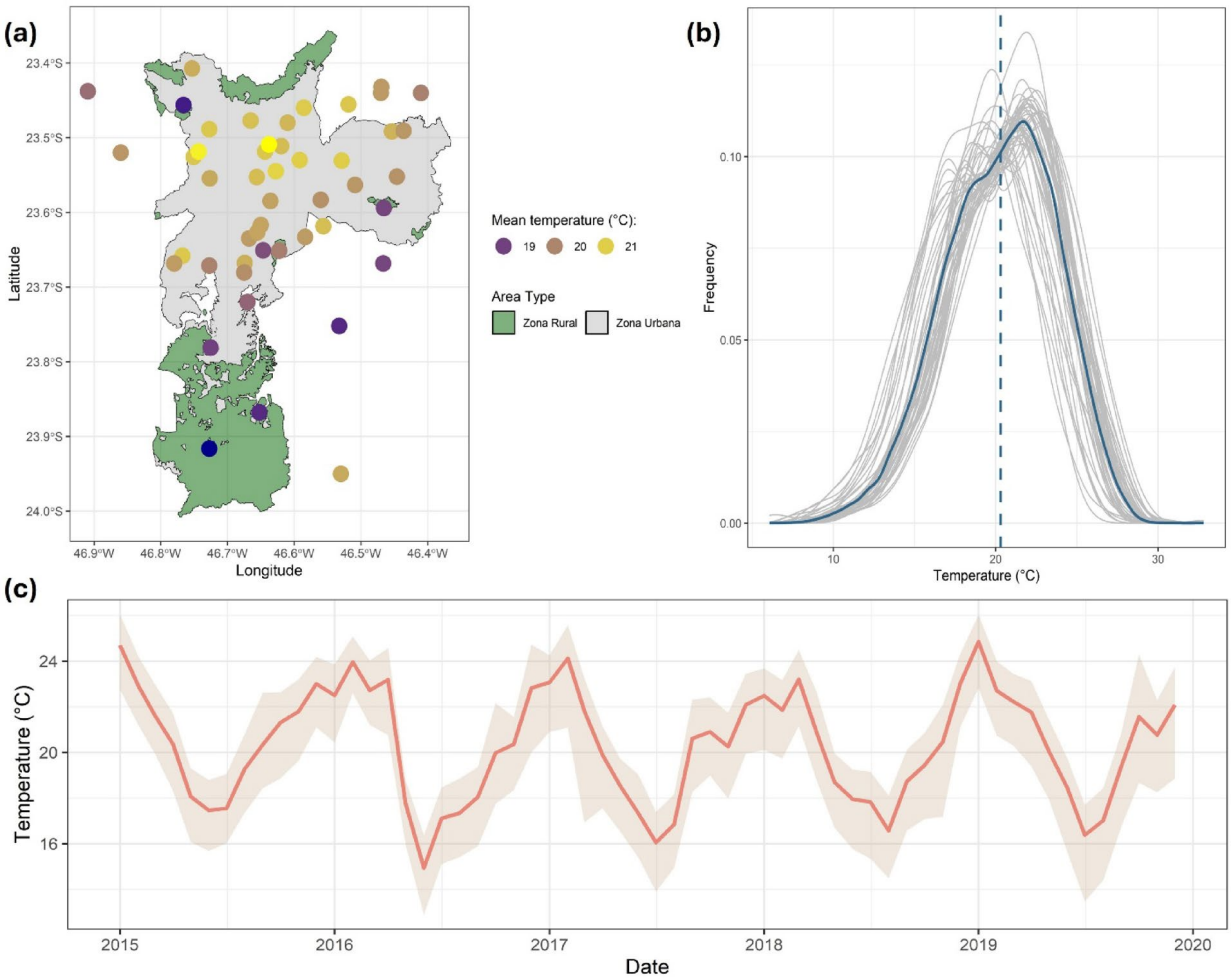
(**a**) Daily mean temperature at each meteorological station averaged over the study period (2015–2019) and overlayed on a map showing area type (rural vs. urban). (**b**) Density plots of daily mean temperature as recorded by each station (grey) and averaged across all stations (blue), with the mean shown as a vertical dashed blue line. (**c**) Monthly average daily mean temperature with the mean of all stations shown as a solid red line, and the maximum and minimum monthly averages across stations as shaded pink.

Between 2015 and 2019, there were 78,569 daily mean temperature recordings across all valid stations, after removing 10% missing values (9,079 days out of 87,648). On average, the highest temperatures were observed in stations located in highly populated urban areas in the city centre (Fig. 3a). The daily mean temperature fluctuated between 6.1 °C and 32.8 °C, averaging at 20.3 °C (Fig. 3b; Table S3). A seasonal pattern was evident, with the warmest months occurring from December to March (average *Ta* = 22.9 °C) and the coolest months from June to August (average *Ta* = 17.2 °C) (Fig. 3c). The monthly averages of daily mean temperature varied between 1.0 °C and 4.0 °C (average 2.0 °C) across stations (Fig. 3c). These differences remained similar at a daily and weekly scales (Figure S7), demonstrating the presence of spatial variation.

We verified all predictor variables met expected criteria. Table S3 provides summary statistics, Figures S8-S9 show expected correlations. The observed correlations were consistent with our understanding of the relationships between temperature and the given predictors. Figure S10 highlights non-linear associations for some of the predictors such as *bsa* or *sza*, supporting the use of RF over linear models.

### Hyperparameter tuning and feature selection

The combination of features and hyperparameters with the best model performance (RMSE = 1.028) were selected, which corresponded to the following 8 features: solar zenith angle *(sza)*, land surface temperature (*lst)*, relative humidity (*rh)*, dew and 2 m air temperature (*d2m* and *t2m)*, eastward and northward wind components (*v10*,* u10)*, and *daylength*, and hyper-parameters: *n_estimators = 1000*,* max_features = sqrt*, and *max_depth = 15.* This was the final combination of features and hyperparameters used to make the predictions. Figure S11 summarizes RMSE values by hyperparameter combination in a boxplot. Figure S12 shows the model performance (RMSE) for the best combination of features for each of the 24 hyper-parameter combinations tested.

### Feature importance

Based on the permutation-based feature importance approach, temperature at 2 m (*t2m*, 0.492) was the most important feature, followed by remote sensed LST (*lst*, 0.144), dew-point temperature (*d2m*, 0.068) and relative humidity (*rh*, 0.052), the eastward wind component (*v10*, 0.028), and day length (*daylength*, 0.023), solar zenith angle (*sza*, 0.014) and the northward wind component (*u10*, 0.011) (Table S4).

### Model predictions

**Fig. 4:**
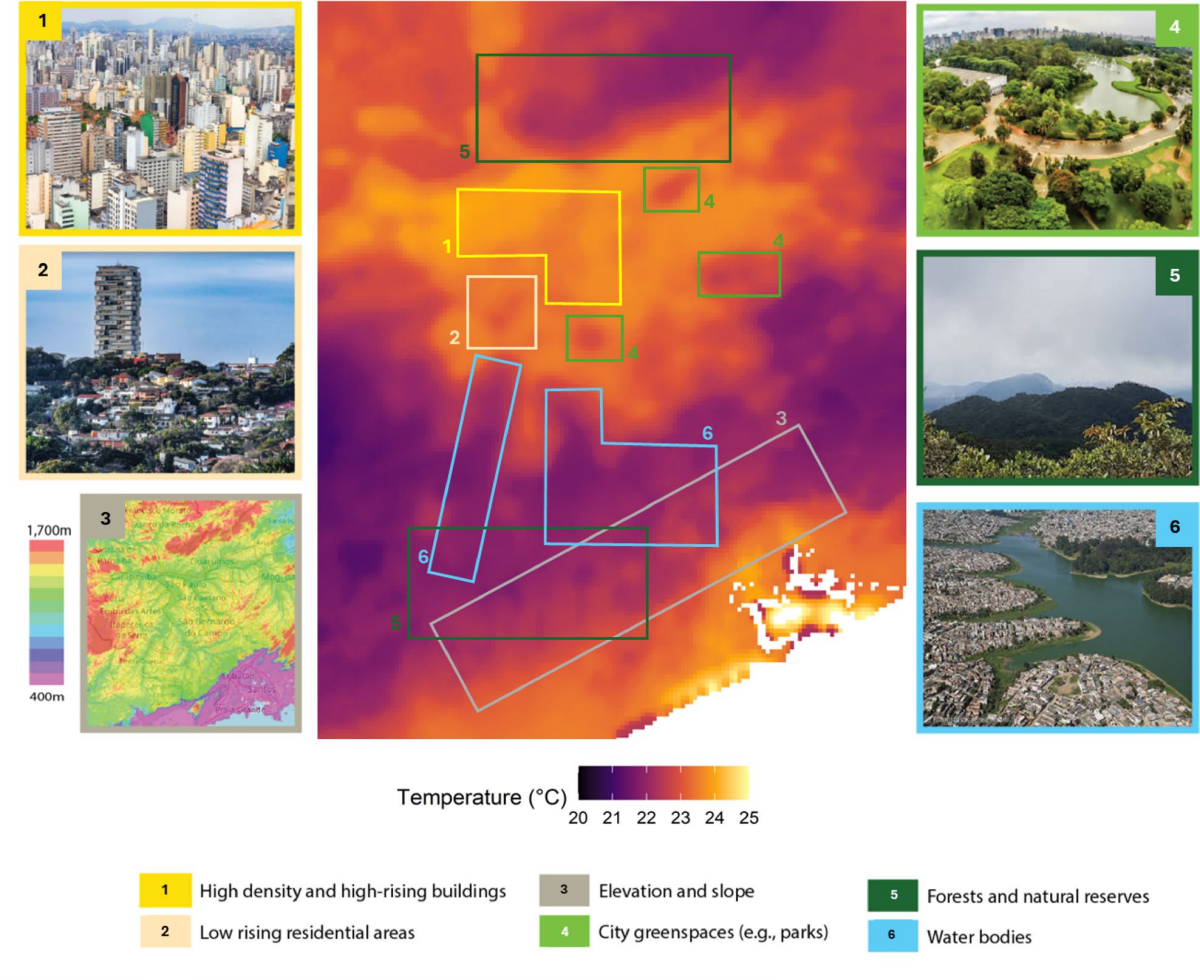
Spatial distribution of predicted average annual daily mean temperatures. The image in the centre shows the average daily mean temperature for the period 2015–2019. Coloured boxes mark distinct spatial patterns in the temperature distribution. On the left and right side, exemplary images of the landscape characteristic for each box, with borders coloured accordingly. Numbers used in the text to refer to specific locations and neighbourhoods in the city.

We used the best model to predict daily mean temperature between 2015 and 2019 at a 500 × 500 m spatial resolution. Figure 4 illustrates the spatial variability of predicted temperatures across São Paulo, as the daily average across 2015–2019. Box numbers in brackets and colour references are used to locate specific locations on the map and correspond to numbers in Fig. 4. For clarity, the same image without the overlaid boxes is provided in Figure S13. Lower temperatures can be seen in areas dominated by large green spaces like the *Parque Estadual Cantareira* (box 1) in the North of the city, or the *Serra do Mar* (box 2), expanding Southeast near the coastline (dark green). The cooling effect of urban parks (light green) can also be observed, such as over the *Parque Ecologico do Tiete* (box 3), *Carmo Park – Olavo Egydio Setubal* (box 4) and the *Parque de Ciencia e Tecnologia da Universidade de São Paulo* (box 5). Large blue spaces, such as *Represa de Guarapiranga* and *Represa Billings* (boxes 6 and 7, respectively; light blue), also exerted a temperature-modulating effect, resulting in lower temperatures. Conversely, the warming effect of built-up areas, characterized by high prevalence of impervious surfaces, high building density, and presence of anthropogenic heat sources, was evident in the city centre (boxes 8 and 9; yellow and beige), with temperatures up to 5 °C higher than in the nearest rural surroundings. Temperature variations were clear between rural and urban areas, while distinctions among different urban types were weaker and sometimes only perceptible at specific temporal resolutions. For example, the difference between the city centre and affluent residential neighbourhoods like *Jardins*,* Itaim Bibi*,* Butanta*,* Perdizes*,* Pinheiros*, and *Brooklin* (boxes 8 and 9; yellow and beige) was more pronounced in the hottest and coolest months (Figure S15). Despite not being direct predictor variables, slope and elevation effects were indirectly captured, possibly through the *t2m* and *lst* variables. For instance, high mountain ranges and significant elevation changes, such as those in the south and across the *Serra do Mar* (box 10; grey), contributed to distinct temperature patterns, concentrating warmer temperatures on the seaside due to the blocking effect of high mountains on warm breezes.

Maps of the average temperature predicted for each month and year are available in Figure S14 and S15. Years 2015 and 2019 were the warmest, with mean annual temperatures of 20.1 °C and 20.0 °C, respectively. The hottest months occurred from December to March, while the coolest months spanned from May to August, aligning with the seasonal oscillations observed in the ground truth data (Fig. 3).

### Model evaluation and validation

**Fig. 5:**
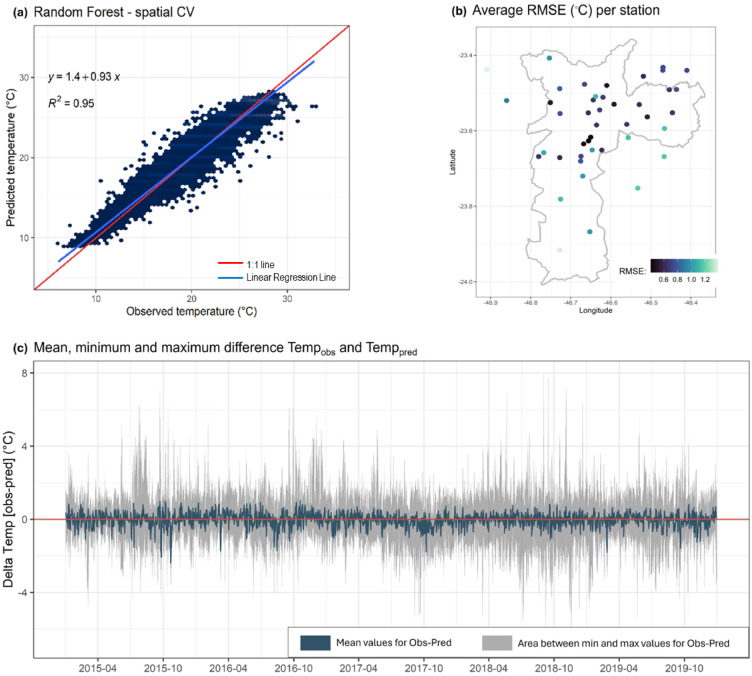
Station-based cross-validation results. (**a**) Density scatter plot of daily mean temperature observed at the meteorological stations (x-axis) and that predicted by the RF model (y-axis), for all study period (2015–2019), assessed through station-based CV. The red and blue lines represent the 1:1 line and the linear regression, respectively. The R^2^ and regression equation shown for each plot. (**b**) Average RMSE (°C) per station for the period 2015–2019, assessed through spatial-CV. (**c**) Mean (dark blue), minimum and maximum (shaded) difference between observed and predicted daily temperatures across all stations for each day in the study period (2015–2019). The red line indicates the absence of differences (i.e., difference = 0).

We validated the model using ten-fold station-based CV at various temporal resolutions (monthly and annually) and area characteristics (urban and rural) using: (i) scatter plots of observed against predicted values with a fitted linear regression (Fig. 5); (ii) the RMSE and R^2^ coefficients as metrics of performance (Table S5); and (iii) box plots of the difference between daily observed and predicted (∆Temp). The results are presented aggregated by temporal scale (monthly and annual) and spatial scale (urban vs. rural) to evaluate potential variations in model performance across time and space (Figure S17-20). Finally, we estimated the temporal and spatial error (Table 1).

Based on the ten-fold station-based CV, the model demonstrated a strong fit (R^2^ = 0.95) and low error (RMSE = 0.80 °C) over the entire period (Table S5), yet a slight tendency to underestimate high temperatures and overestimate low ones was observed (Fig. 5a). No clear systematic error was discernible over time when looking at daily variations (Fig. 5c). Across years, the model accuracy remained similar, with 2016 exhibiting the highest accuracy (R^2^ = 0.96) and the second-lowest error (RMSE = 0.79 °C) (Table S5; Figure S16a). Temperature differences showed a mean difference of − 0.03 °C with minor variation (Standard Deviation (SD) = 0.06 °C) (Figure S16(b)). Occasional deviations in predictions were observed, with rare instances of up to 8 °C lower or 4 °C higher than expected temperatures. Monthly variations revealed slightly lower accuracy during hot months, particularly January, February, and March, with R^2^ < 0.9 and higher RMSE values (Table S5; Figure S17a). The mean difference across all months remained negligible at − 0.03 °C, with minor variability (SD = 0.07 °C; Figure S17b). The model performance was generally lower over space, and with different performance across different areas. For example, the RMSE for stations in the city centre being the lowest (Fig. 5c). The same spatial gradient remained when stratified by year (Figure S18). Further investigation confirmed higher model accuracy for stations located in urban (R^2^ = 0.95) compared to rural settings (R^2^ = 0.91) (Table S5; Figure S20) with a tendency for the model to slightly underestimate temperatures recorded by rural stations (mean = -0.22). Outliers were observed in the model underestimating temperatures by up to 7–8 °C.

Table 1 shows the annual R^2^, intercept and slope associated to the spatial and temporal component of the error measured through regression. The model showed a good R^2^ for the temporal component (R^2^ = 0.96; year-to-year variation: 0.95–0.97); whilst a substantially lower R^2^ for the spatial component (R^2^ = 0.65; year-to-year variation: 0.58–0.69). Finally, the slope values close to one indicated that there was little to no bias in the CV results, for either the temporal or the spatial component.

**Table 1:** Model accuracy by year (spatial and temporal component).

Year	Spatial component			Temporal component		
	*R* ^2^	Intercept	Slope	*R* ^2^	Intercept	Slope
2015	0.70	0.14	0.99	0.95	0.00	1.02
2016	0.64	0.54	0.98	0.97	0.00	1.02
2017	0.69	− 1.40	1.06	0.96	0.00	1.01
2018	0.58	− 0.06	1.00	0.96	0.00	1.01
2019	0.66	− 0.25	1.01	0.96	0.00	1.01
Overall	0.65	− 0.21	1.01	0.96	0.00	1.01

Finally, based on the external validation with the five hold-out stations, our model showed good performance (RMSE = 1.00 °C; R^2^ = 0.92). The accuracy of the model varied slightly by station, with R^2^ ranging from 0.81 to 0.98 (Table S6). The worst performance was recorded for station CETESB 19 (RMSE = 1.74 °C; R^2^ = 0.86) with predictions systematically higher than the observed (Figure S20). Together with the A744, CETESB 19 is the only other rural station of the hold-out dataset.

### Results from the sensitivity analyses and model comparisons

The model performance for the three sensitivity analyses, overall and across all groups, was virtually identical to the main model, demonstrating the robustness of the model. More information on the sensitivity analyses model performances and spatial agreement of predictions is included in Table S7 and Figure S21.

To quantify the added value of this RF model over traditional simpler models, we compared it to a MLR model. Overall, the RF exhibited a better performance than the MLR (RMSE_RF_=0.80 °C; R^2^_RF_ = 0.95, and RMSE_MLR_=1.02 °C; R^2^_MLR_ = 0.92) (Table S5; Figure S23a). This superiority persisted when compared by year, months and urbanicity classification (Table S5). When evaluated using the hold-out dataset, the RF model (R^2^_RF_ = 0.92 and station-to-station variation_RF_: 0.81–0.98) was slightly superior to the MLR model (R^2^_MLR_ = 0.90 and station-to-station variation_MLR_: 0.79–0.95) (Table S5; Figure S23b).

The distribution of differences indicated that the RF tended to predict slightly higher temperatures, which was corroborated when comparing predictions from the RF and MLR models overall, annually and monthly (Figure S23a-c). Differences between annual estimates (Figure S23b) were also minor whilst slightly larger differences were observed for monthly predictions (Figure S23c). RF predicted lower temperature for the coolest months of April to August, and higher temperatures for the warm months of November to January. When considering spatial distribution (Figure S24), the MLR model captured broadly the same patterns as those observed in the RF model, with some exceptions such as for the area nearing the coastline and the northern parts of the prediction area where the MLR model seemed to predict higher temperatures than the RF model (∆Ta < 0 °C). The spatial patterns persisted across the years (data not shown). When examining extreme values within the prediction range (Figure S23), RFs tended to produce a narrower range of predictions, demonstrating the poorest overall ability to predict extremes, which aligns with expectations given its underlying functioning.

## Discussion

Our study demonstrated the feasibility and value of using a RF algorithm to predict daily mean temperature at a 500 × 500 m resolution in settings with a spatially heterogeneous distribution of ground measurements and data with non-linear data associations. Our approach integrated multiple earth observation products and re-analysis data, relying exclusively on open-access data and employing a parsimonious configuration to facilitate model transferability, interpretability, and reproducibility. The model demonstrated good performance in capturing the major temporal variations, with some limitations noted in capturing extreme conditions and spatial variation.

### Spatiotemporal predictors

Despite the substantial number of predictors investigated, a simpler model with eight variables yielded the best performance minimizing overfitting. Our variable selection prioritized a balance between model performance, overfitting reduction, and the generalizability of the modelling pipeline. Consequently, land-use data were excluded for only data for one year was available, making it temporarily static. Similarly, highly localized variables, e.g., latitude or longitude, were excluded for their inclusion can lead to overfitting due to their high spatial autocorrelation^49^. Instead, we focused on variables with strong seasonal and spatial patterns. Some studies include land-use variables for mechanistic reasons despite their limited model importance and risk of overfitting^23^; yet, our goal was to develop a model that is both robust and accurate and not overfitted to a location and time.

In our RF model, ERA5-Land variables were key predictors, with 2 m temperature (*t2m*) being the most important predictor. Some studies have suggested that ERA5-Land temperature products can be used alone in epidemiological studies in the absence of ground measurements^9,10,53,54^. When compared to local temperature recordings, they showed good alignment at the city-level^10^. Nevertheless, there is evidence that ERA5-Land generally performs better at lower temperatures than at higher ones, with factors such as distance to the coastline and altitude influencing its accuracy^53,55^. Additionally, ERA5-Land’s performance declines in urban areas^53^, where it struggles to accurately capture the UHI effect and extreme temperature events^55^. As a result, while these datasets may be suitable to be used directly in regional health impact studies or city-wide analyses, their limitations may introduce biases in suburban and highly urbanized areas. In these settings, combining ERA5-Land with other datasets, such as LST, can provide more accurate temperature estimates.

LST is a key factor influencing *Ta* in urban environments, affecting surface radiation, energy exchange processes, and human comfort^56^, being a critical predictor in many studies^20,31,43,52,57–60^. This was not the case in our study. Previous research has highlighted variability in the LST-temperature relationship by ecosystems and regions^61^, season^62^ and time of the day^58,63,64^. For example, Zhu and colleagues found that night-time LST was a strong predictor of minimum temperature (RMSE = 2.97, R^2^ = 0.94), whilst daytime LST was less effective in predicting maximum temperature (RMSE = 7.45, R^2^ = 0.83)^58^. During the day, more complex, non-linear associations are observed due to interactions with other factors, such as satellite-sun geometry and surface properties, potentially weakening LST’s predictive power. This may explain why LST was not the most important variable in our model. Additionally, ERA5-Land temperature variables, which had not been included in previous studies (Do Nascimento et al., 2022) likely captured much of the spatiotemporal variation explained by LST.

### Spatial and Temporal distribution of predicted temperature

The model showed the expected seasonal changes. *daylength* was included to account for sunlight duration variations, yet its lower score in permutation-based feature importance suggests that temporal variations were primarily captured by other variables, such as *lst* and *d2m.* Despite this, given the potential season-dependent association between LST and *Ta*^62^, retaining the daylength variable seemed valuable for the model to accurately identify seasonal changes.

The model showed a relatively poor performance in the spatial component (R^2^ = 0.65). This is likely to be linked to the inability of the available stations to capture the complexity of the landscape and its spatial variability or missing key covariates in the model. However, the model did successfully capture many large-scale features in the area. Several ‘cool islands’ were observed in the city centre, coinciding with the location of major parks, as well as larger greenspaces in the North and South. Vegetation cover influences the surface thermal conditions^65^ and the evaporative control of energy portioning^66^, resulting in lower temperatures. After LST, NDVI is probably the most common variable used to model temperature^20,48,67–69^. NDVI was not selected by our model feature selection process, probably due to the presence of uncaptured modifying variables such as vegetation type, season, altitude, and climate region as shown in other studies^20,31,58,62^, the presence of other variables like *lst*, *rh*, and *t2m* which were may have indirectly incorporate the effects of NDVI, or coarse temporal and spatial resolution of the data used. The two dams/lakes in the mid and southern regions also showed lower temperatures, despite features directly linked to water bodies were not included in the model due to their static nature. Similarly, despite not being explicitly included, the model successfully captured topographical elements like elevation and slope, reflecting lower temperatures in higher elevation areas such as *Cantareira National Park*, home to *Pico do Jaraguá*, the municipality’s highest mountain. In short, the model accurately captured temperature variations between peri-urban/rural and urban areas aligning with prior research from São Paulo^70^, emphasising the limitations of using point data for exposure assessment.

The ability of the model to perform well spatially relies on the number and distribution of the ground meteorological stations used for training, which was limited to 43 stations for training. The heterogenous and complex characteristics of the region and the lack of enough stations, may explain the poorer performance of the model on the spatial over the temporal component. This underscores the value of in-situ measurements for model training and accuracy, highlighting the need to expand station networks, particularly in the Southern Hemisphere where coverage is lacking^24,61^. This calls for urgent action to enhance coverage, especially in rapidly urbanizing regions, to effectively monitor urban heat fluctuations^7,24,25^.

### Comparison with previous temperature models

Compared to other studies predicting daily mean temperature, our model (RMSE = 0.80 °C; R^2^ = 0.95) showed overall similar R^2^ but lower errors. Kloog and colleagues achieved a RMSE_global_ of 2.16 °C and R^2^_global_ of 0.95 when estimating daily mean temperature prediction over the Northeast and Mid-Atlantic USA at 1 × 1 km resolution^52^. Similarly, Shi and colleagues obtained a RMSE of 1.38 °C and R^2^ of 0.97 for the Southeastern USA using a mixed model with day-specific random effects^71^. Gutierrez-Avila reported an average RMSE of 1.14 K and R^2^ between 0.78 and 0.95 for the Mexico City^68^, while Kloog achieved a global RMSE of 1.68 °C and R^2^ of 0.95 over France using a linear mixed model^72^. Rosenfeld explored MODIS Aqua and Terra LST data products for modelling daily mean temperature, reporting RMSE values of 0.70 °C and 0.67 °C and R^2^ values of 0.986 and 0.987, respectively^73^. Bussalleu obtained a RMSE of 1.3 °C over Europe between 2003 and 2020 at 1 × 1 km resolution using RF algorithm^23^. Notably, in recent years, there has been an effort to bring down the resolution with some notable studies going as fine as 250 m in Catalonia, Spain^74^ or 100 m in Switzerland^75^. Both studies, reported high r-squared values (above 0.95) and relatively low RMSE values (below 2°C). Despite the similarities, comparisons should be interpreted with caution as there are substantial differences in the geographic location, study area size, statistical modelling approach, temporal resolution and coverage, and meteorological station density. Most notably, there is a lack of studies investigating large cities in LMICs, such as São Paulo, making direct comparisons challenging.

Some studies employed a two-stage approach for estimating temperature. The initial stage involved filling in missing values in LST using spatial-temporal predictors, creating gap-filled surfaces for temperature prediction^23,76^. Although this approach may have potentially yielded more locally tailored LST gap-filled products, we chose to utilize an existing and validated global LST gap-filling methodology^27^, favouring user-friendliness and transferability.

Compared to linear regression models, machine learning approaches, and particularly RFs, show better performance. Zhang and colleagues found that models accommodating non-linearities outperform linear ones, particularly when input data quality is poor^48^. Xu et al. observed that RF models achieved better accuracy in predicting maximum temperature over British Columbia, Canada, at a 1 × 1 km resolution (Mean Absolute Error (MAE) = 2.02 °C, R^2^ = 0.74) than MLRs (MAE = 2.41 °C, R^2^ = 0.64)^77^. Similarly, Ho and colleagues found RF (MAE = 2.31 °C) to outperform MLRs (MAE = 2.46 °C) when predicting daily maximum temperature at 1 × 1 km over Great Vancouver, Canada^57^. Dos Santos and colleagues confirmed the superiority of machine learning algorithms, including RFs, in urban temperature modelling over linear regression approaches when modelling daily maximum temperature over Greater London between 2006 and 2017 at 1 × 1 km resolution^20^. When comparing RF to other machine learning algorithms, the findings are more heterogeneous. Mohsenzadeh Karimi highlighted RF’s advantage over support vector machines and artificial neural networks for predicting monthly temperature in Iran^78^. Dos Santos found that gradient boosting algorithms (RMSE = 2.03 °C and R^2^ = 0.68) slightly out-performed RFs (RMSE = 2.13 °C; R^2^ = 0.65) in urban temperature modelling in Greater London^20^.

Our study supports the use of RF algorithms for modelling environmental variables over linear methods, specially thanks to RF’s capacity to capture complex interactions and non-linear relationships with low computational demands. Although RFs may not always outperform MLR, they offer resilience to overfitting; accommodate complex and non-linear associations; have low computational demands are less sensitive to parameter choices, and are easier to interpret than other machine learning approaches, making them attractive for various applications^45,79,80^.

### Relevance for urban studies

Urban areas exhibit distinct physical characteristics such as building density, layout, and green spaces, influencing local energy balance and wind patterns, creating city micro-climates with strong effect on the temperature distribution^81–83^ and temperature-mortality association^84^, Numerical models which exploit known physical and geometric principles are known to produce accurate and highly resolved temperature estimates, yet their complex and high memory and computational costs limit their usability over extensive areas and periods. Reanalysis data such as ERA5^85^, ERA5-Land^35^ or CHIRTSdaily^24^, provide accessible and ready-to-use temperature data, but their spatial resolution is inadequate for sub-urban studies. Most studies predicting temperature in urban areas predict at a spatial resolution of 1 × 1 km^20,57,59,68^. Only one other study^86^, conducted over Tel-Aviv, Israel, achieves daily a higher resolution (30 × 30 m), although with slightly lower performance (RMSE = 1.58; R^2^=0.92). Thus, access to reliable temperature data at an adequate spatial and temporal resolution for sub-urban studies (often below 1 × 1 km) remains a limiting factor in epidemiological studies^5^, particularly in LMICs^7^. To the best of our knowledge, our model is the first to provide daily mean temperature estimates at 500 × 500 m.

### Strengths and limitations

A major strength of this model lies in the high spatiotemporal resolution and accuracy of the data. Its fine temporal and spatial granularity can enable aggregation over different periods, such as trimesters or seasons, allowing for detailed examination of temperature trends and fluctuations. Additionally, the data can be analysed at varying spatial scales, facilitating the study of specific groups or regions, such as *favelas* or areas with high levels of socio-economic deprivation. By focusing on these areas, researchers can explore localized temperature patterns, their impacts on vulnerable populations, and the broader implications for climate adaptation and urban planning strategies.

A second major strength is the use of a streamlined model design, prioritizing simplicity, and novel feature selection approaches to mitigate overfitting and enhance its broader application and transferability. By using FFS, we effectively reduced the risk of overfitting avoiding the inclusion of variables with a strong spatial autocorrelation which depict highly local information, such as latitude and longitude^49^. Finally, we intentionally limited the predictors to open-access datasets, ensuring the framework is easily replicable, adaptable to different settings, and retrainable with updated data when needed. Although designed for transferability using widely available methods and data, this model’s actual performance depends on local context, predictors, and ground data. Validation is essential to ensure the approach remains robust. In some cases, it may be necessary to retrain or expand the model.

Finally, the use of a dense network of meteorological stations, consisting of 48 high-quality stations for a relatively small area, is a key improvement to previous studies using fewer stations^20,59^. This enabled the possibility to model temperature at 500 × 500 m resolution, particularly beneficial for sub-urban studies. Finally, by using both ten-fold station-based CV and external validation, we provided a robust error estimation and model performance evaluation.

Capturing temperature extremes with RF models presents challenges, especially when extrapolating beyond the training data. This can affect the spatial distribution, particularly if the training points do not cover extreme conditions well. To address these challenges, our modelling framework was trained on a dataset spanning a wide range of meteorological conditions, including many meteorological stations located in built-up areas, which naturally tend to capture heat hotspots and extreme high temperatures and thus, helping ensure that the model can capture such events within the prediction domain, provided they are represented in the training data. Moreover, when compared to MLR, the underestimation of the extremes was minor while RF had an overall better performance. Nevertheless, if future or unobserved conditions exceed the training range—such as unprecedented heatwaves or cold spells—the RF model may underestimate or truncate those extremes, emphasizing that predictions should be interpreted with this limitation in mind. Future work could address this by integrating hybrid approaches, such as combining RF with physically based models or applying bias-correction methods, to better capture extremes beyond the training distribution.

Concerns also arise about predictor data quality, especially with satellite data, which can be affected by various issues like atmospheric contamination and cloud cover, affecting model accuracy. Additionally, using a limited selection of open-access features may limit model improvement. Another limitation of this study is the lack of explicit uncertainty estimates associated with the temperature predictions. While the random forest algorithm provides robust point estimates, it does not inherently quantify prediction uncertainty. Methods such as quantile regression forests could be used in future work to better characterize the spatial and temporal variability in model uncertainty.

Finally, despite the relatively dense network of stations available for this study, our model exhibited only moderate performance in capturing spatial variability, particularly in rural areas. Initial analyses indicate a tendency to overestimate temperatures in these regions, likely due to their under-representation in the training data. Although the random forest model captured well large-scale spatial temperature patterns, it does not explicitly account for spatial autocorrelation in the residuals. Future work incorporating spatially explicit or hybrid modelling approaches could help improve spatial performance. The uneven distribution and limited number of monitoring stations in certain areas, could also explain the poorer spatial performance by not adequately capture the diverse landscapes across the study area. In urban settings, the density of stations was higher, ensuring a better fit. These findings underscore the importance of a well-designed and spatially balanced monitoring networks. The modest spatial performance observed, especially in rural areas, warrants careful consideration when applying the model in epidemiological studies that rely on spatial contrasts.

## Conclusion

This study provides daily mean temperature estimates over São Paulo at a 500 × 500 m resolution which will facilitate temperature assessment for epidemiological studies. To our knowledge, this is the first ever open-access dataset to provide daily mean temperature estimates at such a high spatial resolution for São Paulo or any large Latin American city, allowing researchers to perform epidemiological studies at an unprecedented spatial granularity in the region. It also serves as a demonstration of the feasibility of using a RF algorithm and open-access only data to produce accurate and unbiased temperature estimates that outperform traditional regression methods. The model proved particularly effective in urban areas, where most population reside, making it a valuable resource for both urban epidemiological studies. It also highlights the importance of denser monitoring networks across heterogenous and large areas to improve model accuracy and stability in peri-urban and rural settings.

## Supplementary Information

Below is the link to the electronic supplementary material.

Supplementary Material 1

## Data Availability

The datasets generated and analysed during the current study are available in this [Zenodo repository](https:/zenodo.org/records/15868840?token=eyJhbGciOiJIUzUxMiJ9.eyJpZCI6ImNkM2ViZTYzLTUzZjctNDVmYy05NjZjLWViZWQxYWFlNmM4MyIsImRhdGEiOnt9LCJyYW5kb20iOiIxNDQ3ZTAwNDFmNjkwN2Y2YTViNmViYjQzYzcyYWRiYyJ9.jR0CTaNtlOunm0XQYBwjC73yFJIdsjtSXUe-F94VXx5a1vgsCEeQP5-XIPnsRa36rV-fZZuCsw4WtZeDMI9IPA)^87^. All the code used in the analyses is available on GitHub at (https:/github.com/AinaRB/DailyTemperature_RandomForest_SaoPaulo) . Additionally, a public-facing website providing accessible, layman-friendly information about the project and its findings can be found at the project’s website: (https://ainarb.github.io/climate_and_health/)^88^.

## Abbreviations

BSA: Black sky albedo
CV: Cross-validation
d2m: Dew temperature at 2 m
DEM: Digital elevation model
ECMWF: European centre for medium-range weather forecasts
ERA5: 5th generation European centre for medium-range weather forecasts (ECMWF) atmospheric reanalysis
FFS: Feature forward selection
GEE: Google earth engine
LMIC: Low and middle-income countries
LST: Land surface temperature
MLR: Multi-linear regression
NDVI: Normalized difference vegetation index
R2: r-squared
RF: Random Forest
rh: Relative humidity
RMSE: Root mean square error
SZA: Solar zenith angle
t2m: Ambient temperature at 2 m
Ta: Ambient temperature
u10: Northward wind component
v10: Eastward wind component
