# Supplementary material for: A satellite based machine learning approach for estimating high resolution daily average air temperature in a megacity in Brazil
Source: Sci Rep. 2026 Feb 5;16:7459. doi: 10.1038/s41598-026-35689-x (PMC12929572; doi:10.1038/s41598-026-35689-x)
Supplement: Supplementary file 1 — Supplementary Material 1 [file 41598_2026_35689_MOESM1_ESM.docx]

**SUPPLEMENTARY MATERIAL**

**A satellite based machine learning approach for estimating high resolution daily average air temperature in a megacity in Brazil**

**Aina Roca-Barceló**^a^, **Rochelle Schneider**^b,c,d^, **Monica Pirani** ^a^, **Alessandro Sebastianelli** ^b,e^, **Frédéric B. Piel** ^f, a^, **Paolo Vineis** ^a^, **Adelaide Cassia Nardocci** ^g^, **Daniela Fecht** ^f, a^

^a^ MRC Centre for Environment and Health, Department of Epidemiology and Biostatistics, School of Public Health, Imperial College London, United Kingdom

^b^ Φ-lab, European Space Agency (ESA), Frascati, Italy

^c^ Forecast Department, European Centre for Medium-Range Weather Forecast (ECMWF), Reading, United Kingdom

^d^ Faculty of Epidemiology and Population Health, London School of Hygiene & Tropical Medicine, London (LSHTM), United Kingdom

^e^ Euro-Mediterranean Center on Climate Change (CMCC), REMHI Division, Caserta, Italy

^f^ UK Small Area Health Statistics Unit, Department of Epidemiology & Biostatistics, School of Public Health, Imperial College London, United Kingdom

^g^ Department of Environmental Health, School of Public Health, University of São Paulo, Brazil.

**Conflict of Interests:** The authors declare that they have no known competing financial interests or personal relationships that could have appeared to influence the work reported in this paper.

**Corresponding author:**

Dr Aina Roca-Barceló

90 Wood Ln, London W12 0BZ, United Kingdom

Email: [ainaroca16@gmail.com](mailto:ainaroca16@gmail.com)

Telephone: +34 663 132 280

Contents

[S1. Temperature indicator: daily mean temperature 3](#_Toc212732670)

[S2. Temperature data processing 3](#_Toc212732671)

[S3. Spatiotemporal predictor variables: missing values 16](#_Toc212732672)

[S4. Spatiotemporal predictors spatial and temporal harmonization 20](#_Toc212732673)

[S5. Metrics for model evaluation 21](#_Toc212732674)

[S6. Multi-linear regression model (MLR) 22](#_Toc212732675)

[References 23](#_Toc212732676)

[Tables 24](#_Toc212732677)

[Figures 29](#_Toc212732678)

## S1. Temperature indicator: daily mean temperature

We selected outdoor daily mean temperature metric for our study because: (i) the data was available and of good quality across most stations in the study area, as opposed to other meteorological measures such maximum and minimum temperature, humidity or wind; (ii) it captured both heat and cold events, as opposed to other measures such as maximum or minimum temperature that focus only on one end of the temperature spectrum, as well as daytime and nighttime temperatures, providing a more representative measure of the overall thermal stress on the population; (iii) previous literature in the region shows little to no effect of humidity on temperature-mortality associations [1], and so a simpler measure was favoured; (iv) it is a widely used indicator in epidemiological research, providing consistence and comparability across studies, and finally, (v) it is a , easily understandable metric, often considered more relevant for public health.

## S2. Temperature data processing

**Operational period:** Initially, we collected data from 55 meteorological stations, each with different operational periods. As an initial assessment, we plotted their data missingness by year (Figure S2.1). Based on the availability of data, we restricted the study period between 2015 and 2019, characterised by the highest proportion of available stations. Stations with no observations between 2015 and 2019 were excluded (n=3), resulting gin 59 stations considered. Hereafter, all data refers to this period and stations.

**
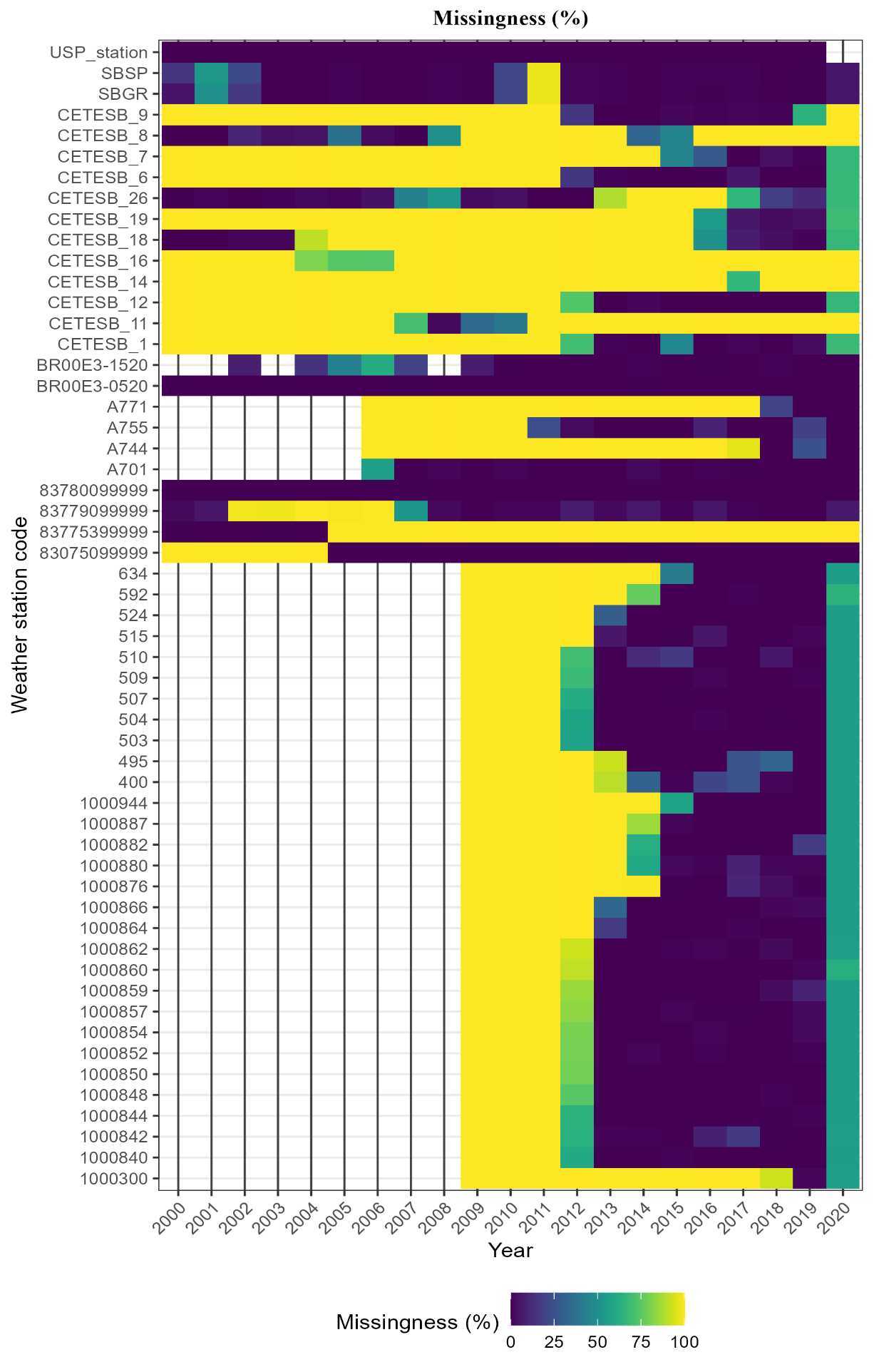
**

**Figure S2.1:** Heatmap of annual missingness as a percentage (%) of the total annual observations by station between 2000 and 2020.

**Spatial clustering:** Prior to the data cleaning, we clustered all 59 stations based on their spatial proximity using k-means spatial clustering. K-means clustering [2] is one of the most used unsupervised machine learning algorithms for partitioning a given data set into a set of *k* groups, where *k* represents the number of groups pre-specified by the analyst. It classifies objects in multiple groups, referred to as clusters, such that objects within the same cluster are as similar as possible (i.e., high intra-class similarity), whereas objects from different clusters are as dissimilar as possible (i.e., low inter-class similarity). In other words, each observation (x*_i_*) is assigned to a given cluster such that the sum of squares (SS) distance of the observation to their assigned cluster centres *µ*k is a minimum. In k-means clustering, each cluster is represented by its centre or centroid, which corresponds to the mean of points assigned to the cluster. In our case, the feature used to cluster the stations was the pairwise physical distance between the monitoring stations. To identify the optimal number of clusters, we iteratively ran the algorithm using different values of *k*. Limiting the maximum number of clusters helps maintain the stability and interpretability of the results by ensuring that each cluster contains an adequate number of samples for reliable estimation and comparison [2]. Accordingly, we capped the maximum number of clusters to below 10. The weighted sum of squares for each iteration is then plotted to identify which *k* value produces the minimum sum of squares. Elbow plots are commonly used for this purpose (Figure S2.2 (a)). It is a line plot with each of the iteration’s Within-Cluster-Sum of Squared Errors (WSS) plotted. The point where the line starts to flatten, the so-called inflection point, indicates the optimal *k* value. This can be hard to visualize sometimes. In those cases, the Silhouette plot can be used (Figure S2.2 (b)). It assigns a value from 1 to -1 to each iteration based on the similarity within clusters and across clusters; the higher the value the higher the similarity within clusters and so the more optimal the *k* value is. Below, we present the results from testing 2:25 *k* values. We selected *k*=7 as the optimal number of clusters and used that value to cluster all the stations. Figure S2.2(c) shows the spatial clusters derived from this procedure.


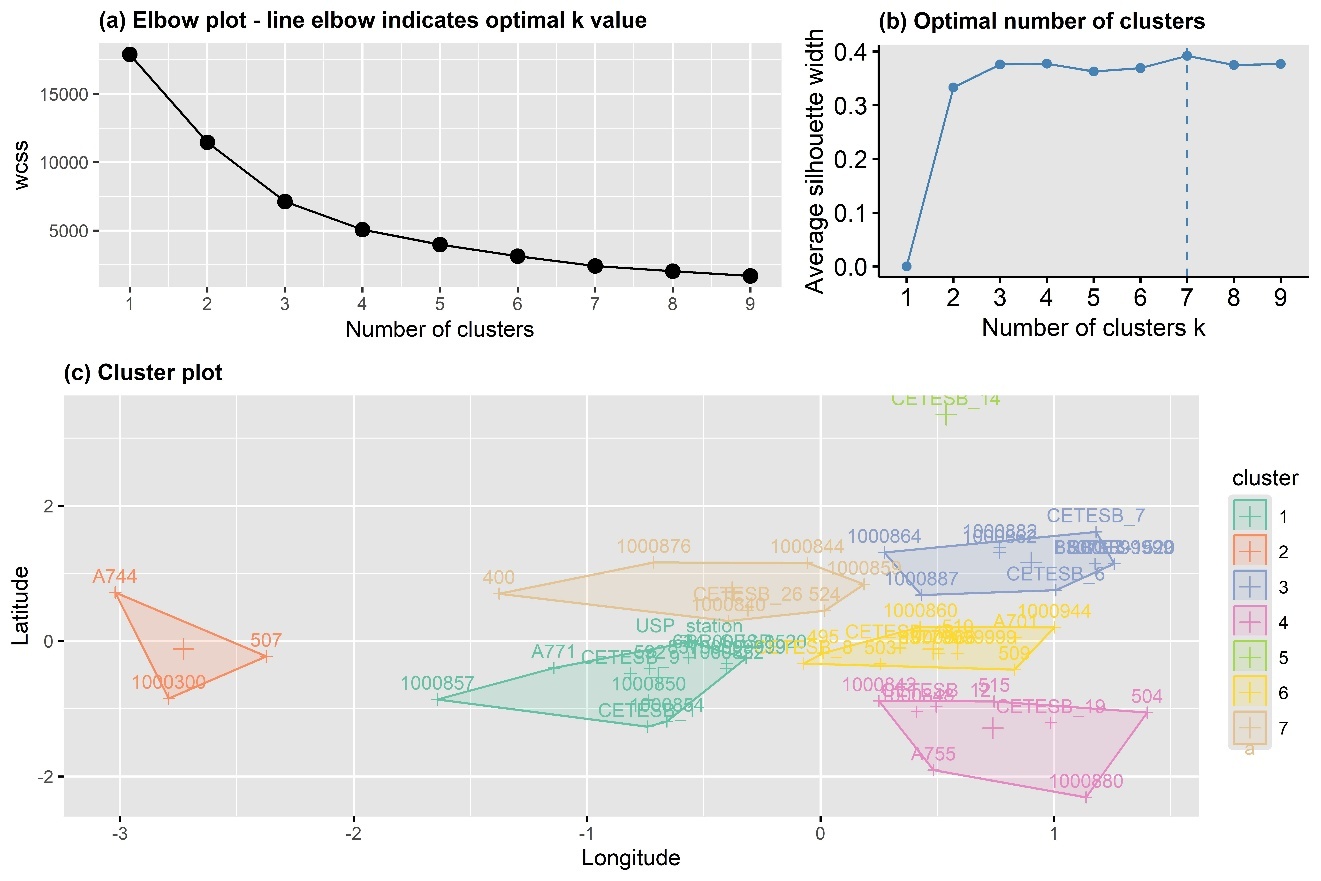


**Figure S2.2:** **Meteorological station spatial clusters.** Elbow plot showing the weighted sum of squares for *k* number of clusters (top left); Line plot of the average silhouette width by *k* number of clusters (top right); Optimal number of clusters (*k*=6) mapped and coloured by cluster (bottom).


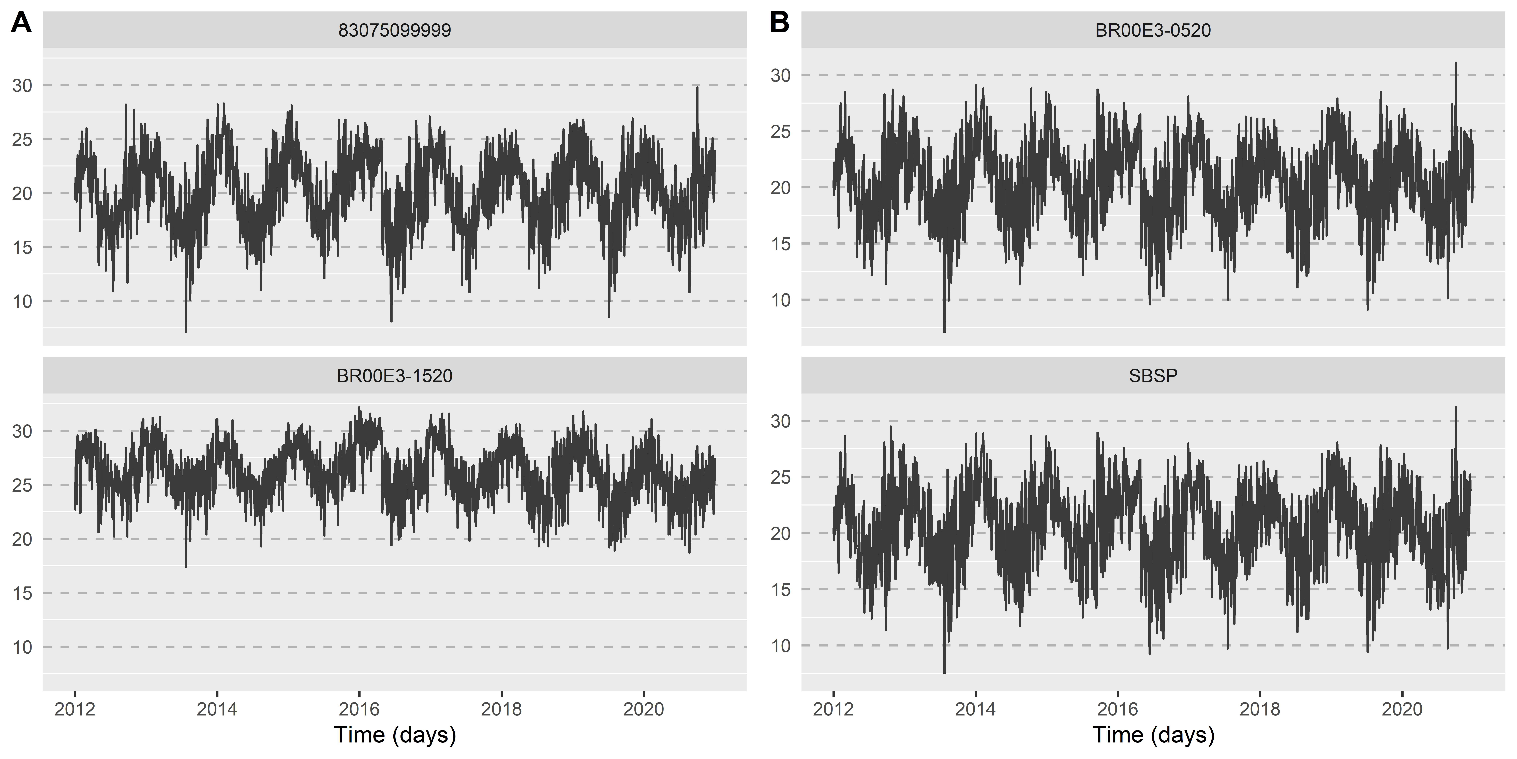
**Duplicated stations:** Stations were considered duplicate if their latitude and longitude coordinates were the same to the third decimal place. Upon identifying a potential suspect of duplicated stations, we confirmed the location in the data providers documentation. There were two pairs of duplicate stations (BR00E3-0520 and SBSP; BR00E3-1520 and 830750999999). We retained the station with a more complete series. For the first pair (Figure S2.3 (a)), despite showing similar oscillations, the BR00E3-1520 station had remarkably higher temperatures (+10 ^◦^C). The differences were large enough to suspect that there was a data issue with one of the stations. To identify which station was the one affected, we compared them to their nearby stations using the spatial clusters defined in the section above. We would expect the temperatures to be similar with stations that are spatially close. The BR00E3-1520 consistently reported temperatures 10 ^◦^C above the other stations in the cluster, and so, it was excluded. For the second pair (Figure S2.3 (b)), the GHCN station (BR00E3-0520) was more complete and thus, retained. This resulted in 53 stations.

**Figure S2.3**: **Meteorological stations duplicates.** Time series of the duplicate stations BR00E30520 and SBSP (A) and BR00E3-1520 and 830750999999 (B).

**Substitution bias:** Several networks report applying basic data imputation methods to fill in gaps in the data. One such method is to fill in missing values with the value recorded on the nearby day. If the window of missing values is large, this can lead to important biases. We refer to this as *substation bias* hereafter. We considered an observation to be affected by substitution bias if the same exact temperature estimate is recorded for more than two consecutive days and for which a similar pattern was not observed in any other station of the same cluster. This can be visually examined using the time series and correlation plots. When such cases were encountered, the affected values were set to *NULL* except for the first one in the series, assuming that it was the true value used to impute the missing following days. We identified two stations affected by at least one event of substation bias.

**Outliers:** Generally, an outlier is an observation that lies an abnormal distance from other values. However, there is no consensus on the exact definition of outliers as it varies depending on the variable of interest and its use. For environmental variables for which extreme values have remarkable health consequences, such is the case of temperature, it is important to consider whether such deviations are indeed data collection/quality issues or real extreme events. In our case, we considered an observation to be a true outlier if the value met both criteria: (i) was at least two degrees above or below the maximum or minimum percentile of a given cluster, respectively, and (ii) was the only observation at that position, i.e., no other station recorded an outlier with a similar value (±1 °C). To identify outliers in the data, we used three complementary tools applied to each individual station, i.e., histograms, normal Q-Q plots and boxplots (Figure S2.4, and S2.5 respectively). In total, 7 observations were considered outliers and set to *NULL*.


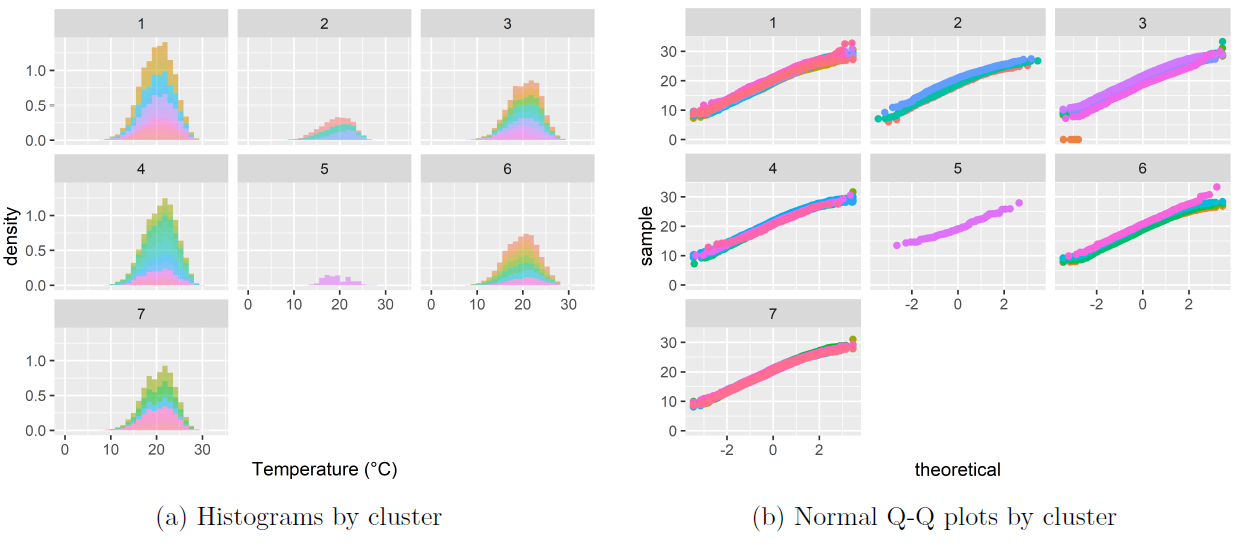

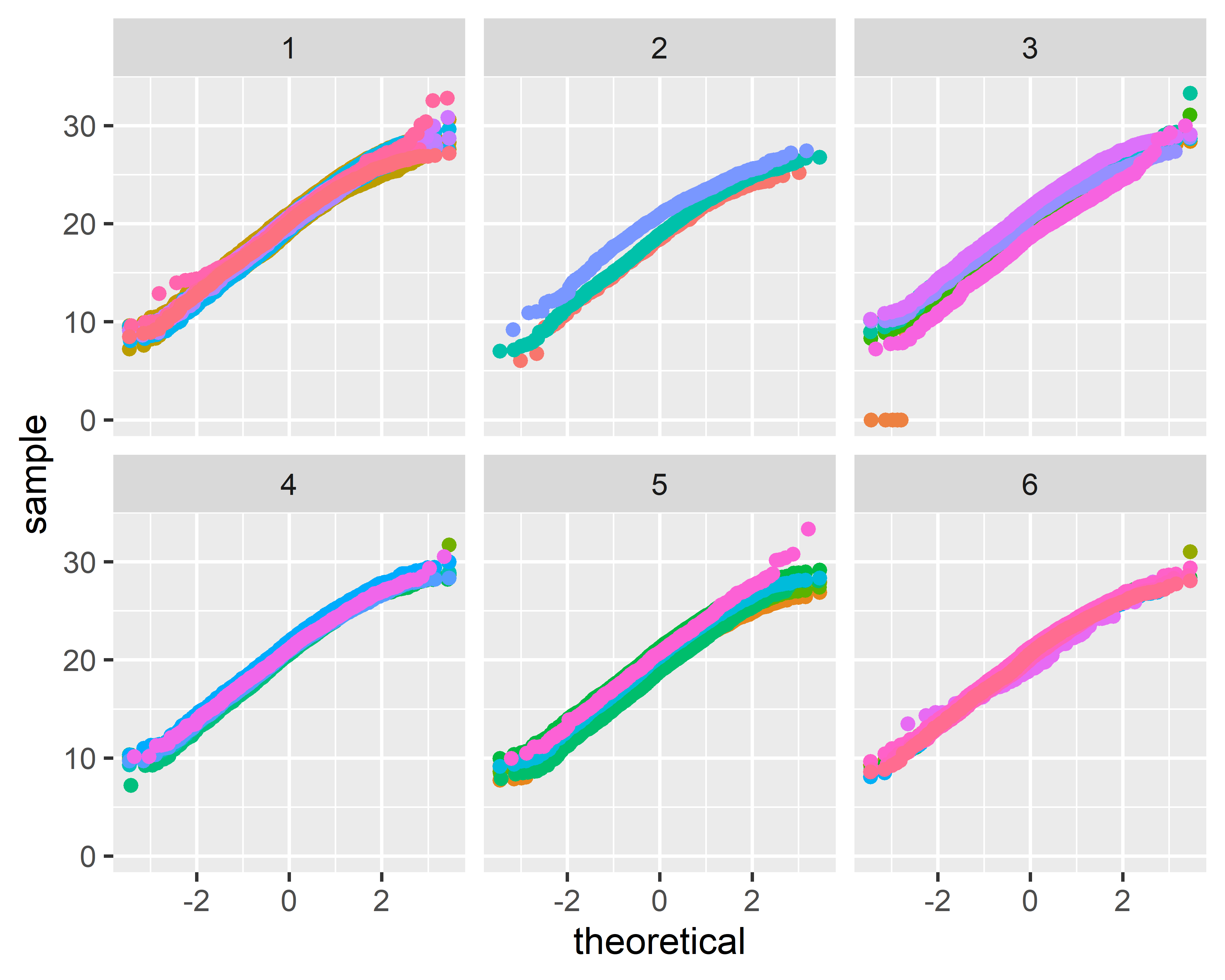

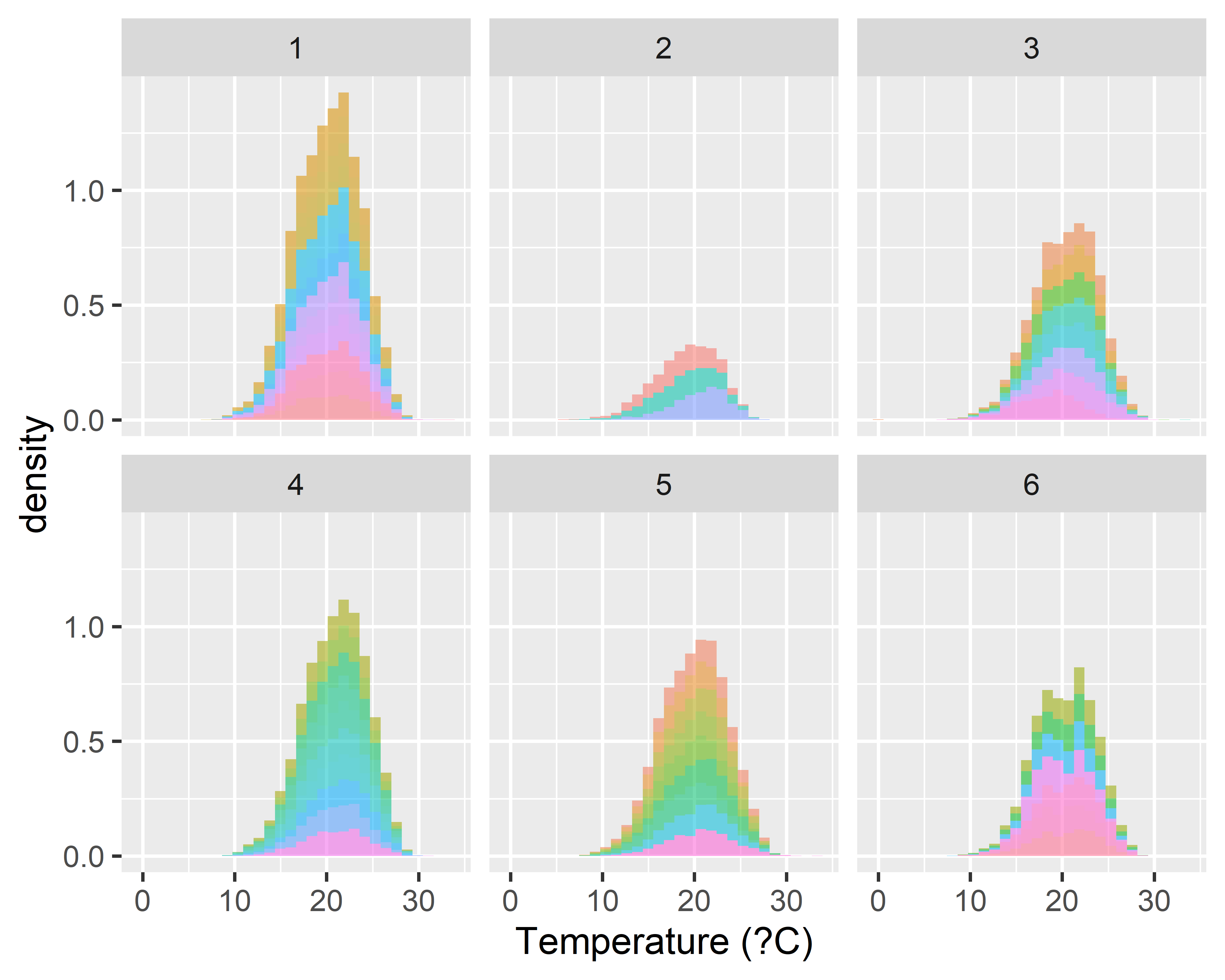


**Figure S2.4**: **Histogram and normal Q-Q plot of temperature by cluster.** (a) Histogram and (b) normal Q-Q plots of the daily mean temperature recordings from each meteorological stations (colors) grouped by spatial cluster (1 → 7).


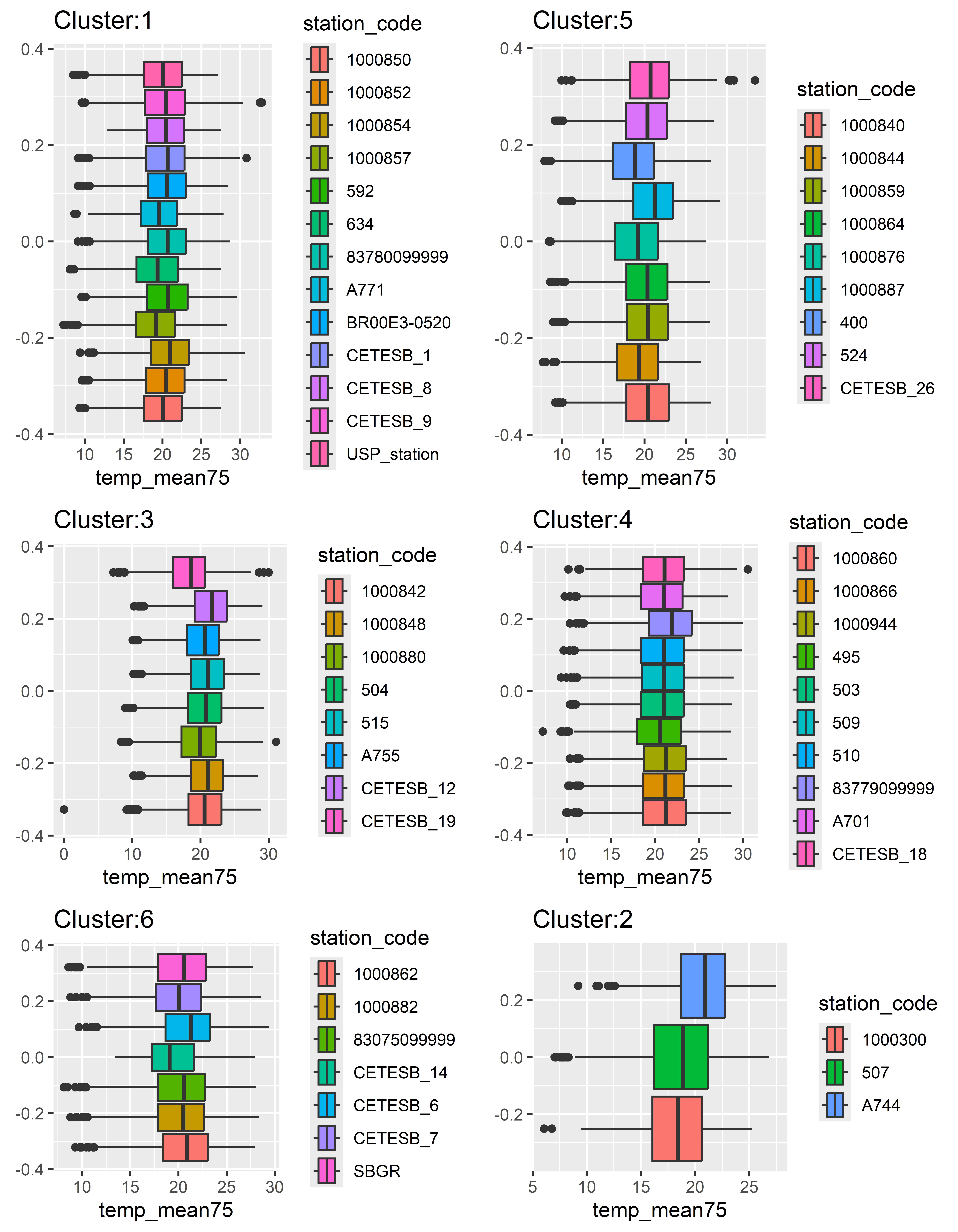


**Figure S2.5**: **Box plot of station-specific temperature by cluster.** Box plots of the daily mean temperature recordings from each meteorological stations (colours) grouped by spatial cluster (1 → 7).

**Completeness:** The Random Forest algorithm requires complete ground truth (target) values for training. Unlike geostatistical methods such as kriging, which explicitly model spatial autocorrelation through covariance structures, Random Forest operates as a non-parametric ensemble learning method that treats observations as independent, without incorporating spatial or temporal relationships unless explicitly included in the feature set through predictor variables. Therefore, it is standard practice to exclude observations with missing target values during training, provided the missingness is random and does not introduce bias. And so, it is important to understand not only the proportion but also the distribution of missing values across the time series. According to this distribution, we defined two classes of missing values:

- **Class 1:** Big windows of missing data during operating period, likely to be associated with faulty or broken equipment. Such events may be arbitrary or associated to vandalism.
- **Class 2:** Finally, missing observations or observations classified as erroneous (e.g., biased, outliers) that occur spread out during the operating period, likely associated to equipment or operator errors. A large proportion of the latter is suggestive of a poor-quality station.

From the 50 stations, we excluded we excluded stations which provided less than one valid year for the entire study period (2015-2019) (n=2), resulting in 48 stations. This restriction tried to ensure that there would be enough training points to capture the associations existing in the unique feature space that was captured by that station. For class 2 and 3, it is important to assess whether missing data are disproportionately affecting a certain set of stations or time periods, as this may introduce a bias to the training dataset and affect its performance. To assess this, we investigated whether they had a differential behaviour over time (day of the week, month, year), network, space (clusters) and area-level characteristics (rurality, deprivation) quantitatively (Table S2.1., with counts (percentage) of missing values per group and chi-square test) and visually (Figures S2.6-9).

- **Temporal bias**. Table 2.1., and Figures S2.6 - S2.7 show the percentage (%) of missing values for each station by year, month and day of the week, respectively. Overall, we observed a slightly higher number of missing data at the start of the study period, with data completeness gradually improving over time. Regarding the months, the highest percentage of missing data was typically observed in the first quarter of the year, with these differences being statistically significant. There was no consistent pattern in the distribution of missing values in the distribution of missing values by day of the week (p-value=0.978).
- **Network bias**. Figure S2.8 (a) shows the percentage (%) of missing values for each station by network. The CETESB was the network with a higher percentage of missing temperature recordings. This is primarily an air pollution network which may explain the lack of prioritization of the temperature recordings when a machine is faulty or damaged. The INMET network of stations also showed a large proportion of missing data, yet in this case it was primarily due to the operational date falling outside the study period for two out of four stations. The rest of networks showed a good annual coverage for the operating period. Differences were statistically significant.
- **Spatial bias.** Figure S2.8 (b) shows the percentage (%) of missing values by cluster. Cluster 1 and 4 suffer from the largest proportion of missing values, which is in line with the network findings as these two clusters are primarily formed by CETESB and INMET stations, and considering they were the smallest clusters (i.e., a smaller number of stations). Differences were statistically significant.
- **Area-level characteristics bias.** We explored whether the percentage of missing values varied with the SES index (Figure S2.9 (a)) of the area where the monitor was found. Devises can be damaged or broken by vandalism, which is more likely to occur in areas of high socioeconomic deprivation. When stratified by socioeconomic status (SES), stations in areas with low SES had a higher proportion of missing data (11.0%) compared to those with high SES (8.7%). Although the difference was statistically significant (p < 0.001), it was relatively small. Finally, we investigated the correlation of missing values with the level of urbanicity of the areas where the monitors are found. We observed a substantially higher proportion of missing data in rural stations (31.3%) compared with urban stations (5.1%), and this difference was statistically significant. By looking at Figure S2.9 (b), there were more stations for which the first years were not available as they were not operative yet. This likely reflects the small population density and thus, need for monitoring in rural settings. It is possible that the lack of services and longer distances may be associated to longer windows of missing data as broken or faulty devices take longer to be repaired. There was no obvious difference in missing data between rural and urban stations.

**Tab1e 2.1.** Number (%) of missing values in daily mean temperature recordings across time (day of the week, month, year), network, space (clusters) and area-level characteristics (rurality, deprivation).

|  |  | **Not missing (%)** | **Missing (%)** | **p-value** |
| --- | --- | --- | --- | --- |
| **Year** | 2015 | 14532 (82.9) | 2988 (17.1) | <0.001 |
|  | 2016 | 15404 (87.7) | 2164 (12.3) |  |
|  | 2017 | 15784 (90.1) | 1736 (9.9) |  |
|  | 2018 | 16759 (95.7) | 761 (4.3) |  |
|  | 2019 | 16894 (96.4) | 626 (3.6) |  |
| **Month** | 1 | 6564 (88.2) | 876 (11.8) | <0.001 |
|  | 2 | 5923 (87.5) | 845 (12.5) |  |
|  | 3 | 6520 (87.6) | 920 (12.4) |  |
|  | 4 | 6446 (89.5) | 754 (10.5) |  |
|  | 5 | 6729 (90.4) | 711 (9.6) |  |
|  | 6 | 6469 (89.8) | 731 (10.2) |  |
|  | 7 | 6765 (90.9) | 675 (9.1) |  |
|  | 8 | 6862 (92.2) | 578 (7.8) |  |
|  | 9 | 6613 (91.8) | 587 (8.2) |  |
|  | 10 | 6890 (92.6) | 550 (7.4) |  |
|  | 11 | 6701 (93.1) | 499 (6.9) |  |
|  | 12 | 6891 (92.6) | 549 (7.4) |  |
| **Day of the week** | 1 | 11325 (90.4) | 1203 (9.6) | 0.978 |
|  | 2 | 11343 (90.5) | 1185 (9.5) |  |
|  | 3 | 11341 (90.5) | 1187 (9.5) |  |
|  | 4 | 11304 (90.6) | 1176 (9.4) |  |
|  | 5 | 11365 (90.7) | 1163 (9.3) |  |
|  | 6 | 11362 (90.7) | 1166 (9.3) |  |
|  | 7 | 11333 (90.5) | 1195 (9.5) |  |
| **Network** | airports | 1819 (99.6) | 7 (0.4) | <0.001 |
|  | cetesb_stations | 11586 (79.3) | 3022 (20.7) |  |
|  | cgs_stations | 52021 (95.0) | 2759 (5.0) |  |
|  | ghcn_noaa | 1825 (99.9) | 1 (0.1) |  |
|  | gss_noaa_stations | 5442 (99.3) | 36 (0.7) |  |
|  | inmet | 4854 (66.5) | 2450 (33.5) |  |
|  | USP_station | 1826 (100.0) | 0 (0.0) |  |
| **Cluster** | 1 | 2862 (52.2) | 2616 (47.8) | <0.001 |
|  | 2 | 10560 (96.4) | 396 (3.6) |  |
|  | 3 | 13696 (93.8) | 912 (6.2) |  |
|  | 4 | 9322 (85.1) | 1634 (14.9) |  |
|  | 5 | 15473 (94.2) | 961 (5.8) |  |
|  | 6 | 15865 (96.5) | 569 (3.5) |  |
|  | 7 | 11595 (90.7) | 1187 (9.3) |  |
| **SES** | Low SES | 24375 (89.0) | 3015 (11.0) | <0.001 |
|  | High SES | 54998 (91.3) | 5260 (8.7) |  |
| **Urbanicity** | Rural | 10029 (68.7) | 4579 (31.3) | <0.001 |
|  | Urban | 69344 (94.9) | 3696 (5.1) |  |


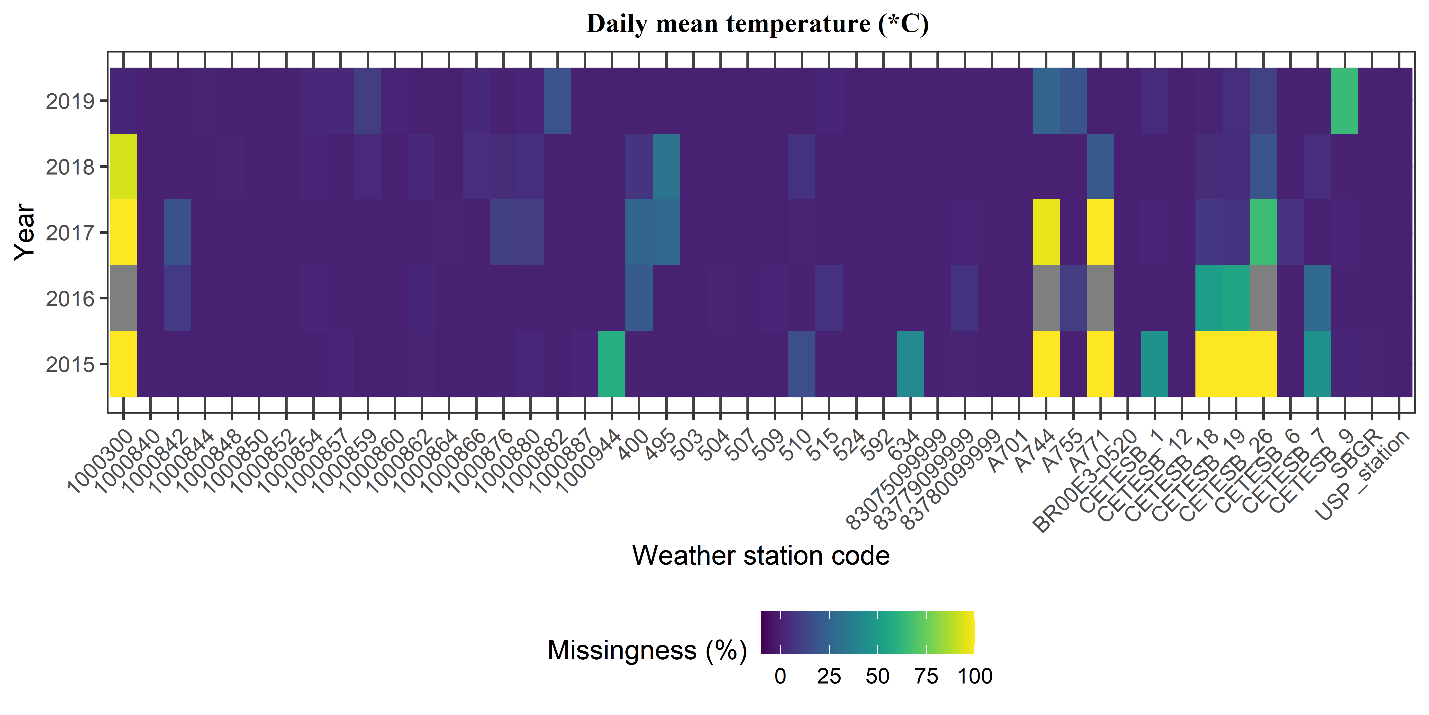


**Figure S2.6:** **Missing values by year** Percentage of daily missing temperature values for each station (*x-axis*) and year (*y-axis*).


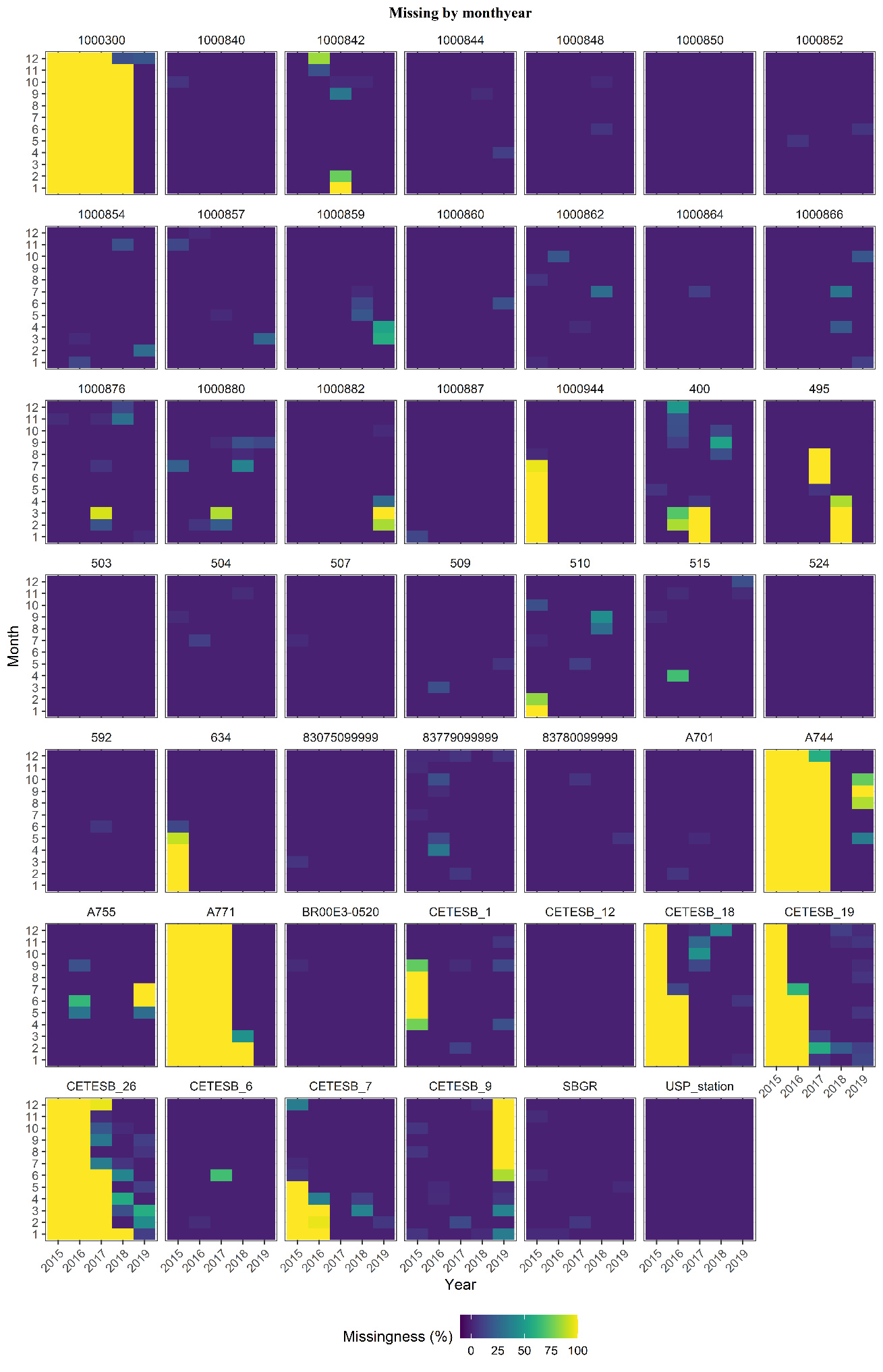


1. Missing values by month.

**Figure S2.7**: **Missing values by month and day of the week.** Percentage of daily missing temperature values for each station by (a) month and (b) day of the week (*y-axis*) across each year (*x-axis*).


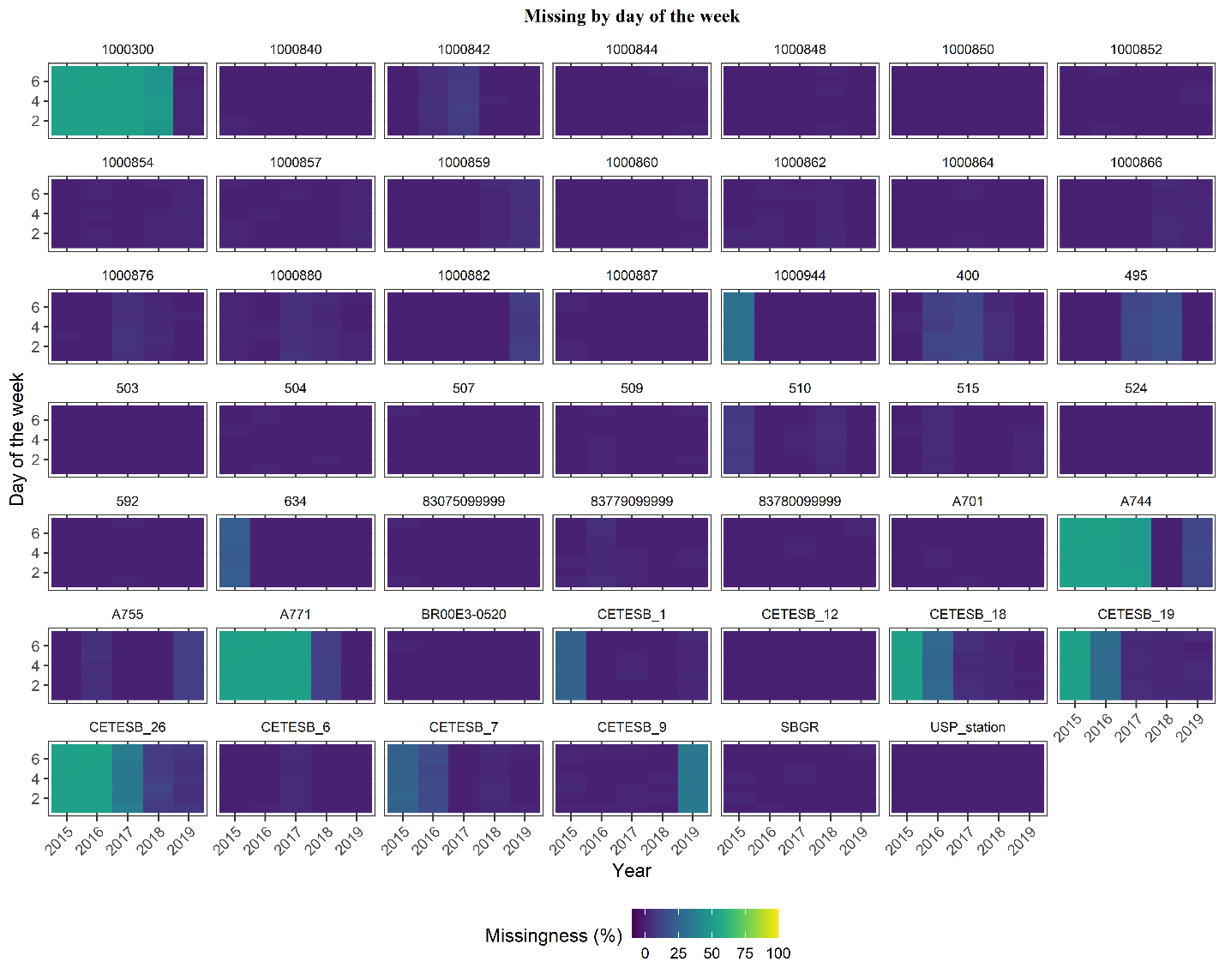


(b) Missing values by day of the week.

**Figure S2.7 (cont.)**: **Missing values by month and day of the week.** Percentage of daily missing temperature values for each station by (a) month and (b) day of the week (*y-axis*) across each year (*x-axis*).


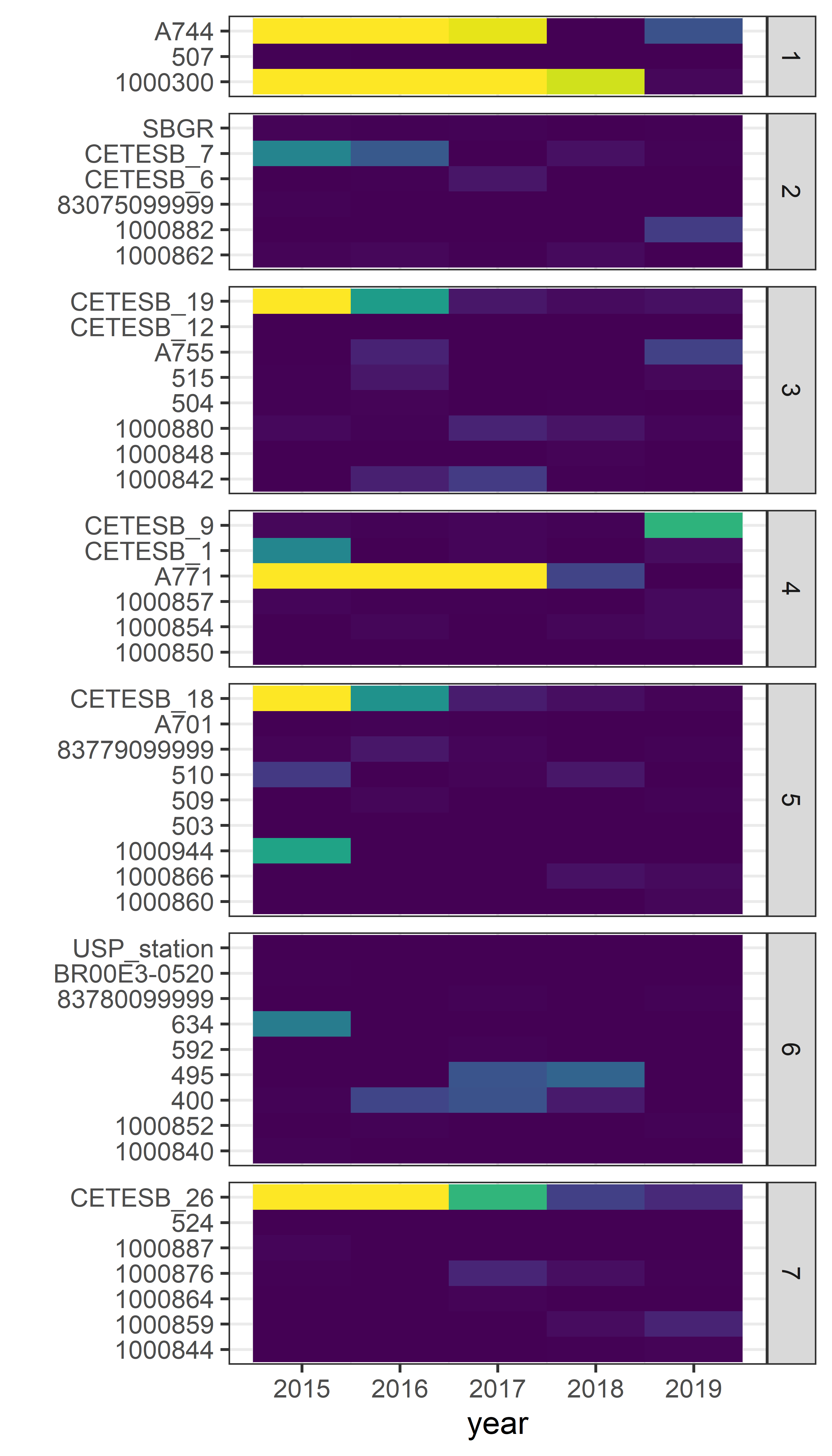


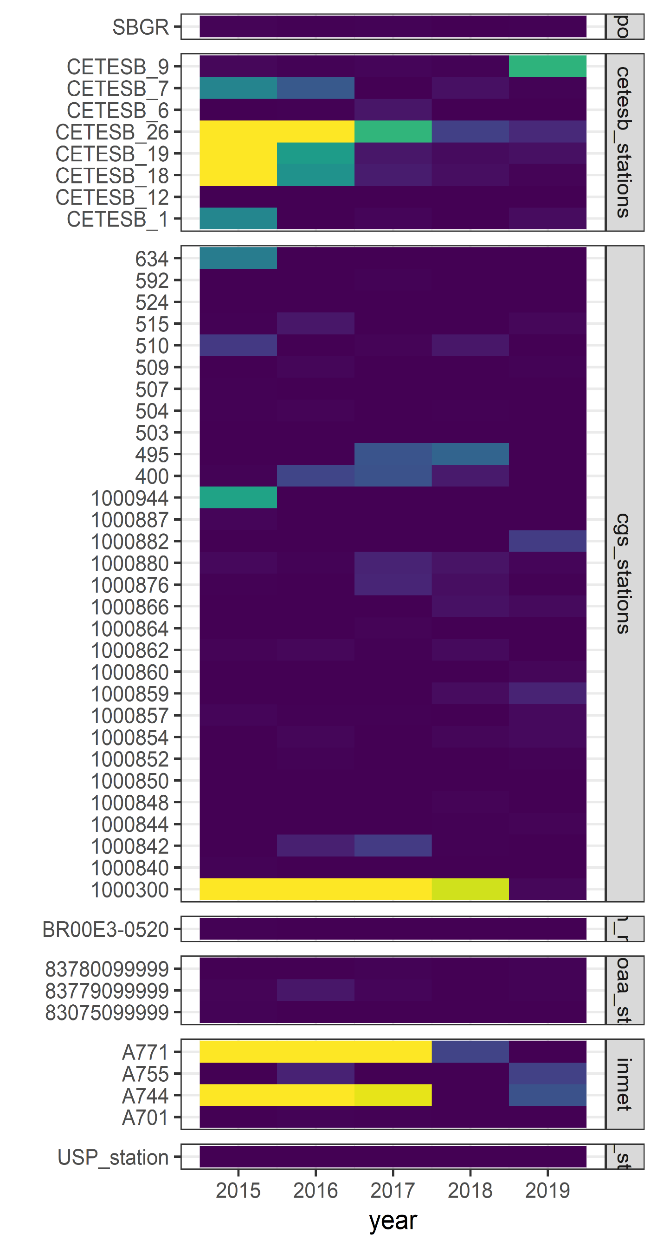

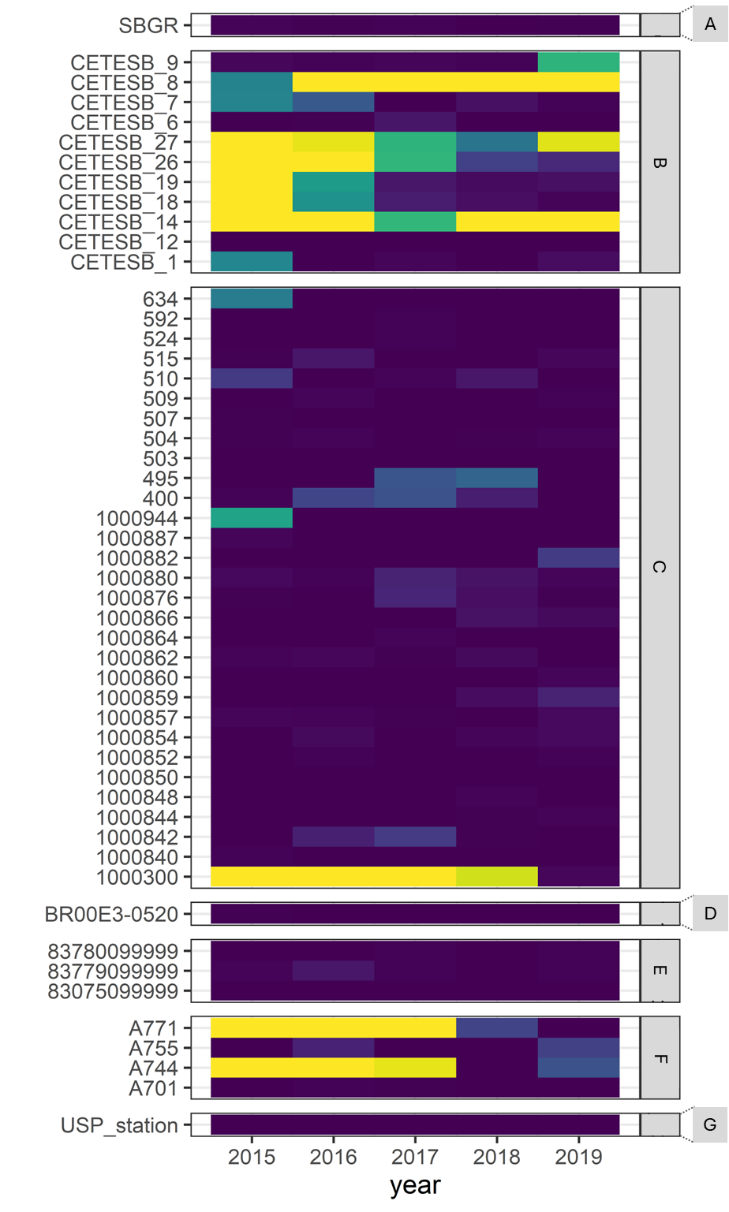

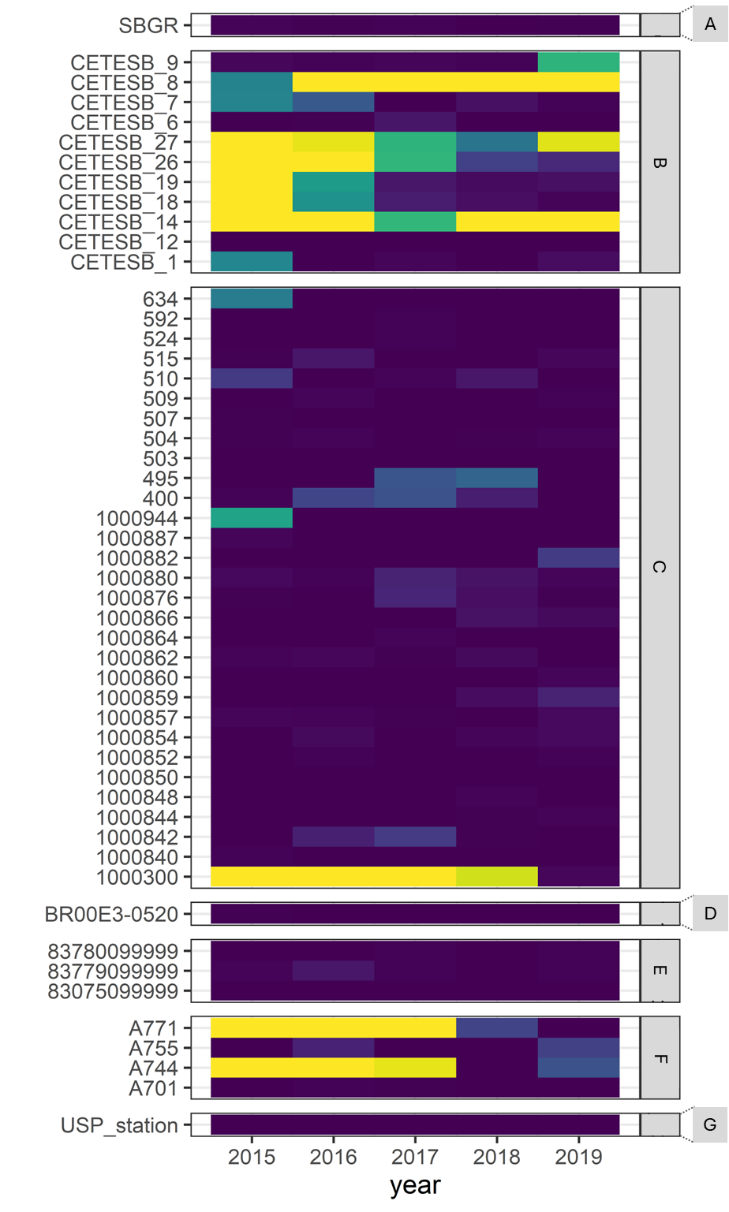


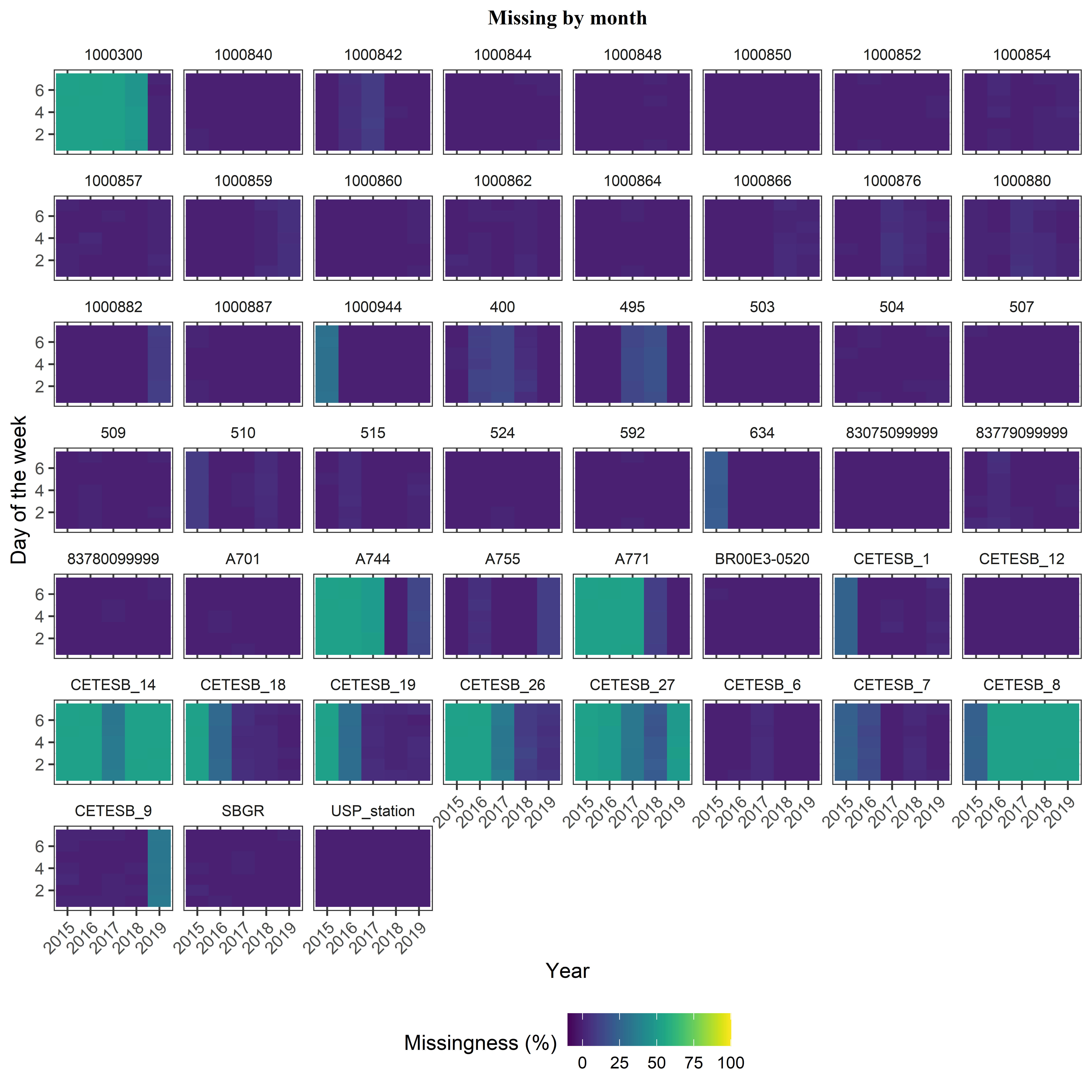


(a) Missing values by network (b) Missing values by cluster

**Figure S2.8**: Missing temperature data by network and cluster. Percentage of daily missing values by year and station, grouped by (a) **meteorological station network** – A: Airport stations; B: CETESTB stations; C: GCS (Global Climate Station); D: GSS (Geostationary Satellite Server, NOAA); E: GHCN (Global Historical Climatology Network); F: INMET (*Instituto Nacional de Meteorologia*); and G: University of São Paulo station – and (b) **spatial cluster**.


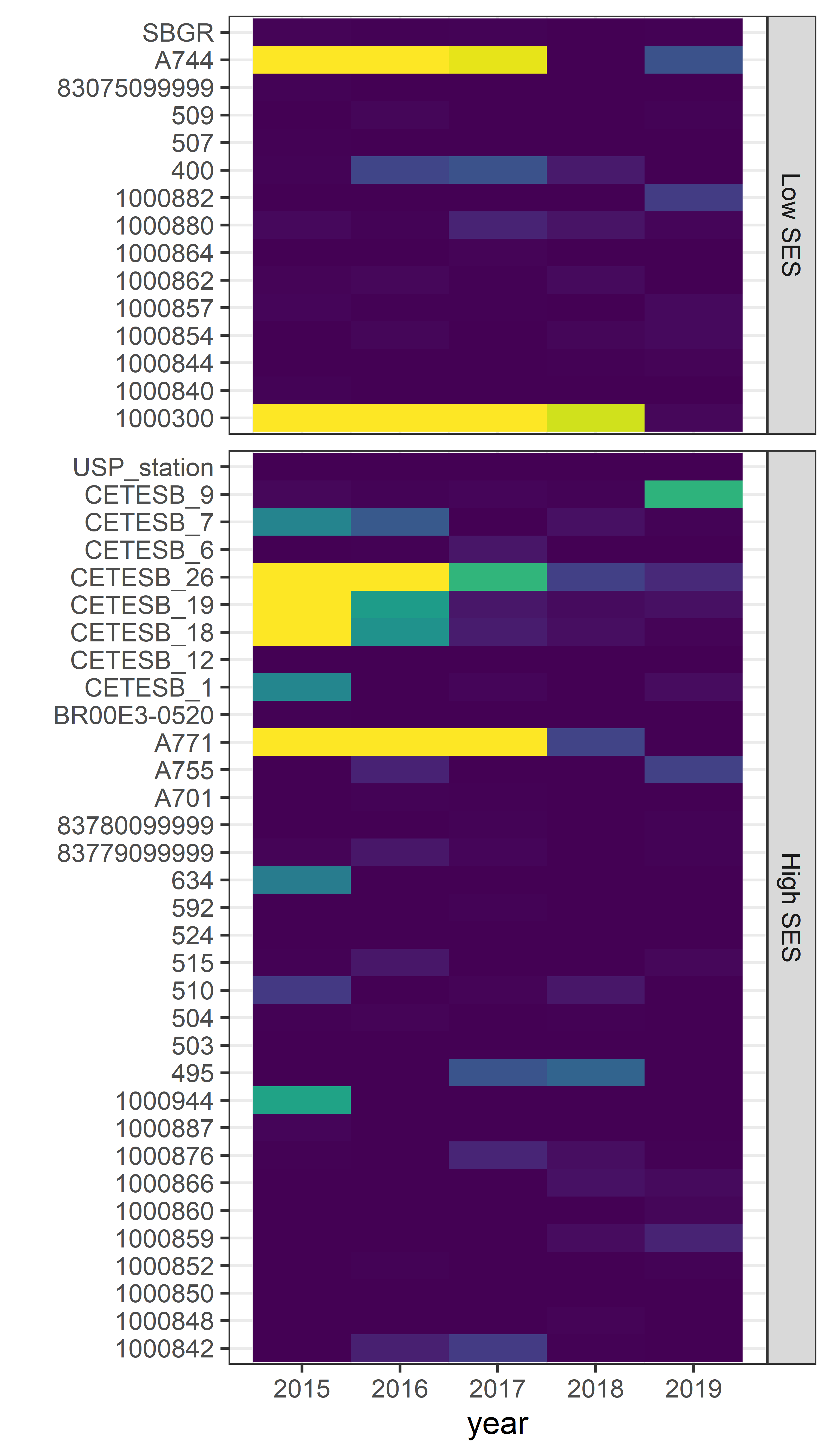

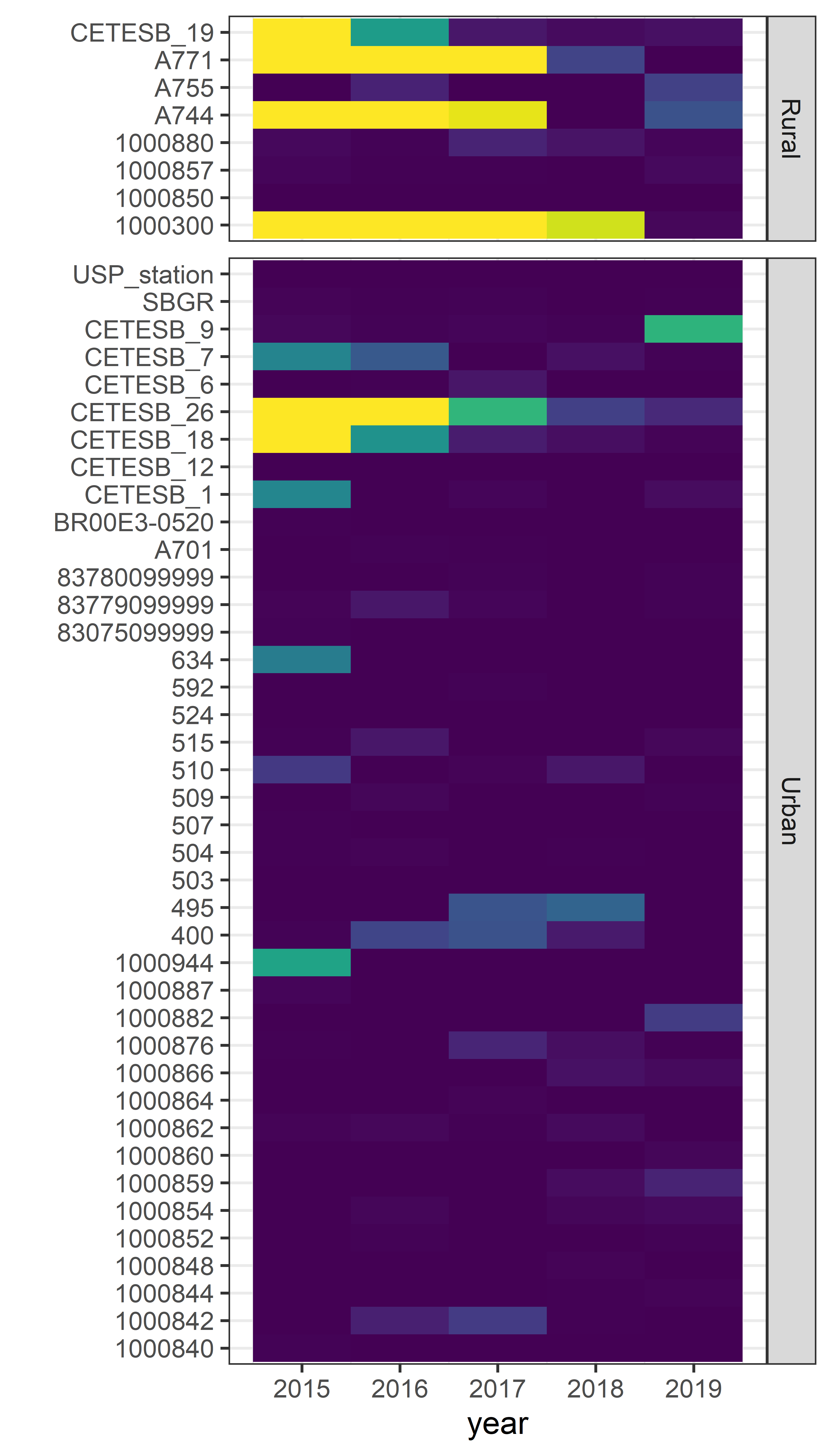


(a) Missing values by SES (b) Missing values by rural/urban

**Figure S2.9.** **Missing temperature by SES and rurality.** Percentage of daily missing values by year and station, grouped by (a) category of socioeconomic deprivation and (b) rural or urban classification of the station.

**Satellite and satellite-derived data collection and initial processing.** For each satellite or satellite-derived product, we proceeded as follows:

1. **Define area of study**. We defined the study area using a rectangular bounding box that matched our area of prediction (lon_min_: -46.9559; lat_min_: -24.0854; lon_max_: -46.2226; lat_max_: -23.2839, coordinate system: WGS84).
2. **Define dates of interest**. We defined the start and end date for the download, i.e., 01/01/2015 to 31/12/2019. The temporal resolution imported varied depending on the temporal resolution of the data product.
3. **Specify data product**. The image collection or data product was loaded. More information on the specific image collection used for each data product can be found in Table and described in detail in each data product specific section.
4. **Cloud-cover mask**. All the imagery used comes from optical sensors. These cannot penetrate clouds. Thus, it is important to detect areas with thick layers of clouds and mask them from the dataset for their poor quality. This step is only required for satellite imagery, not for satellite-derived data products, as these have no cloud problem. MODIS has a cloud mask built-in. Landsat, on the other hand, does not. For Landsat imagery, GEE provides their own cloud scoring algorithm (*ee.Algorithms.Landsat.simpleCloudScore()* ). This algorithm scores each pixel in an image based on its cloudiness. It then adds a band named ‘cloud’ to the input image which contains the exact cloud score ranging from 0 (absence) to 100 (cloudy). The user can then create a mask to flag those pixels which score is above a predefined threshold. A commonly used threshold is 20. Accordingly, we masked any pixels above a threshold of 20.
5. **Reduce spatial resolution**. For those data products that were provided at a resolution finer than our target resolution of 500m^2^we performed a spatial reduction to match it. All images were re-projected to coordinate system: WGS84 (EPSG: 4326).
6. **Output export**. Images at the desired spatial and temporal resolution were exported as *.tif* files.

## S3. Spatiotemporal predictor variables: missing values

**Identification and handling of missing data.** Earth observation data gives us an opportunity to have highly resolved spatial and temporal information at a global scale. Nonetheless, missing values are abundant, most often due to miscellaneous phenomena that interfere in the measurement, such the cloud cover discussed above. This was the case for the features black sky albedo (*bsa)*, solar zenith angle (*sza*), Normalized difference vegetation index (NDVI), and land surface temperature (lst). There are several imputation techniques that can be used depending on the nature of the data and the distribution of the missing values. We first explored the existence of continuous gap-filled datasets already available and validated. This was the case for *lst*, for which a worldwide continuous gap-filled MODIS product exists, developed and maintained by [3]. For all other datasets, we used a *gapfill* imputation method proposed by [4]. Below we explore the approach we took for the different datasets.

**(a) Worldwide continuous MODIS LST dataset, by Shiff et al., 2021** .

It consists of a data fusion method which combines MODIS data with a cloud-mask at 1km^2^ with the Climate Forecast System Version 2 (CFSv2) modelled 2-m daily air temperature [5]. The MODIS dataset provides the granularity on the temporal and spatial variability; the CFSv2 model provides the complete spatial coverage three times a day at the expense of a coarser spatial resolution (0.2^◦^, approx. 20km^2^ in the equator) and so, it can be used to borrow information to fill in the gaps for days/locations with missing data. The CFSv2 is NASA’s fully coupled global NCEP Reanalysis model representing the interaction between the Earth’s atmosphere, oceans, land, and sea ice from 1979 until present. It provides global coverage at a resolution of approximately 20km^2^ with no missing values.

The method is based on the premise that the temperature experienced at a given time and location can be described as the combination of a long-term average temperature for that specific time of the year and location (seasonality or climatology) and the deviation from that average or expected value linked to the weather (temperature anomaly). For each pixel, the seasonality or climatology was derived using Temporal Fourier Analyses (TFA) for both the cloud-free MODIS LST product and the CFSv2 product. The TFA mathematically simplifies complex and noisy data, decomposing it in series of functions or harmonics with specific amplitude and frequency. In this case, the TFA was defined to describe the seasonal cycles of temperature in terms of annual, biannual and tri-annual components (or ‘harmonics’). These Fourier harmonics may be recombined, providing a smoothed signal, which is regarded here as the climatological expected temperatures (*LST_clim_*(s,*t*)). The temperature anomaly (*LST_anom_*(*t*)) for each day and pixel is then calculated as the difference between the observed temperature from the CFSv2 product and the MODIS LST climatological temperature. Finally, each missing pixel in the MODIS LST dataset (*LST_cont_*(*t*)) is replaced by the sum of the MODIS LST climatological temperature (*LST_clim_*(s,*t*)) and the CFSv2 temperature anomaly (*LST_anom_*(s,*t*)) (see Eq. 4.6). These values are then used to replace the cloud affected pixels in the MODIS LST original dataset, creating a continuous gap-free dataset.

${LST}_{cont(s,t)}={LST}_{clim(s,t)}+ T_{anom(t)}$ Eq. (A.1.)

Interactive queries can be made on their web application powered by GEE, yet these have a temporal and spatial limit, far below the requirements of this project. Alternatively, the authors have included extensive documentation and all the code files, including those for validation, in their GitHub account. Using the GEE editor, the code can be used to generate continuous LST data for any period and area of interest. Here, we used this code to generate gap-free continuous daily MODIS LST data for São Paulo study area. The global RMSE and Pearson’s R coefficient for the entire study period and area were 2.34^◦^C and 0.83, respectively (Table S3.1). For detailed documentation refer to [3].

**Table S3.1**: **LST imputation performance metrics.** Annual and global RMSE and Pearson’s R coefficient for the area under study.

| **Year** | **Pearson** | **RMSE (^◦^C)** |
| --- | --- | --- |
| 2015 | 0.83 | 2.37 |
| 2016 | 0.85 | 2.47 |
| 2017 | 0.83 | 2.31 |
| 2018 | 0.8 | 2.21 |
| 2019 | 0.86 | 2.34 |
| Overall | 0.83 | 2.34 |

**(b) Gapfill spatio-temporal interpolation method**

Available imputed datasets are rare. This was the case for the sza, bsa and NDVI datasets. So, missing values were imputed using a spatiotemporal interpolation method called gapfill developed by [4], which can be implemented in R software using the *Gapfill R* package[6]. To ensure consistency, the same imputation method was applied across all three datasets.

Most data imputation methods exploit the temporal dependence of the data. However, remote sensing data both spatial and temporal dependence. The gapfill addresses this challenge and it does so at a low computational expense and providing uncertainty estimates. It has been shown to outperform TIMESAT and gapfill-MAP, both widely used temporal and spatio-temporal imputing methods. The gapfill method consists of four steps. Firstly, for any given missing pixel, one defines a prediction window (like a bounding box) containing the information to use for the imputation. Depending on the spatial and temporal dependence of the data, the size of the window will vary. Secondly, each pixel in the prediction window is given a score based on all the other values in the window. Images are then ranked in an increasing trend based on the score. Thirdly, for each pixel in the prediction window with a non-missing observation one determines the empirical quantile it corresponds relative to all values of the image. The mean of all the empirical quantile levels is then used as the target quantile level. Finally, all the quantile levels are then regressed using a quantile regression with level equal to the target quantile level. The target value can then be predicted based on the fitted quantile regression. An advantage of this method over others is that it provides statistical uncertainty estimates for each prediction. To do so, it estimates the uncertainty associated to each step, which is then combined to provide an overall uncertainty. For more details refer to [4].

The dimensions of the imputation window are key to the performance of the imputation. A window too small will cause the predictions to be noisy; a window too large and the predictions will be too smooth. This window is defined by an *annual, intra-annual* and *spatial extents* (Figure S3.2 (a)). The *annual extent* defines the number of years to consider before and after to account for inter-annual variability. The *intra-annual extent* defines the number of images to consider before and after within one year. The units can vary depending on the temporal resolution of the data (e.g., daily, weekly). Finally, the *spatial extent* controls the number of pixels above/below and right/left considered during the imputation. To select the best parametrization for each dimension, different window sizes were tested using Cross-validation (CV) with 30% random missing values. The accuracy was evaluated using RMSE. The window parametrization with the lowest RMSE was selected. Figure S3.2 (b) shows the RMSE value of the parameters tested. The final window parametrization was (3,2,1) for *sza*, (1,2,3) for *bsa* and (2,2,3) for *NDVI*, for the annual, intra-annual and spatial extents, respectively.

Following the general process described above, we imputed the missing values in the *bsa*, *sza*, and *NDVI* datasets. Figure S3.3 shows the original dataset with missing values (top) and the imputed dataset for a randomly selected day of the *bsa*, *sza*, and *NDVI* datasets (bottom).


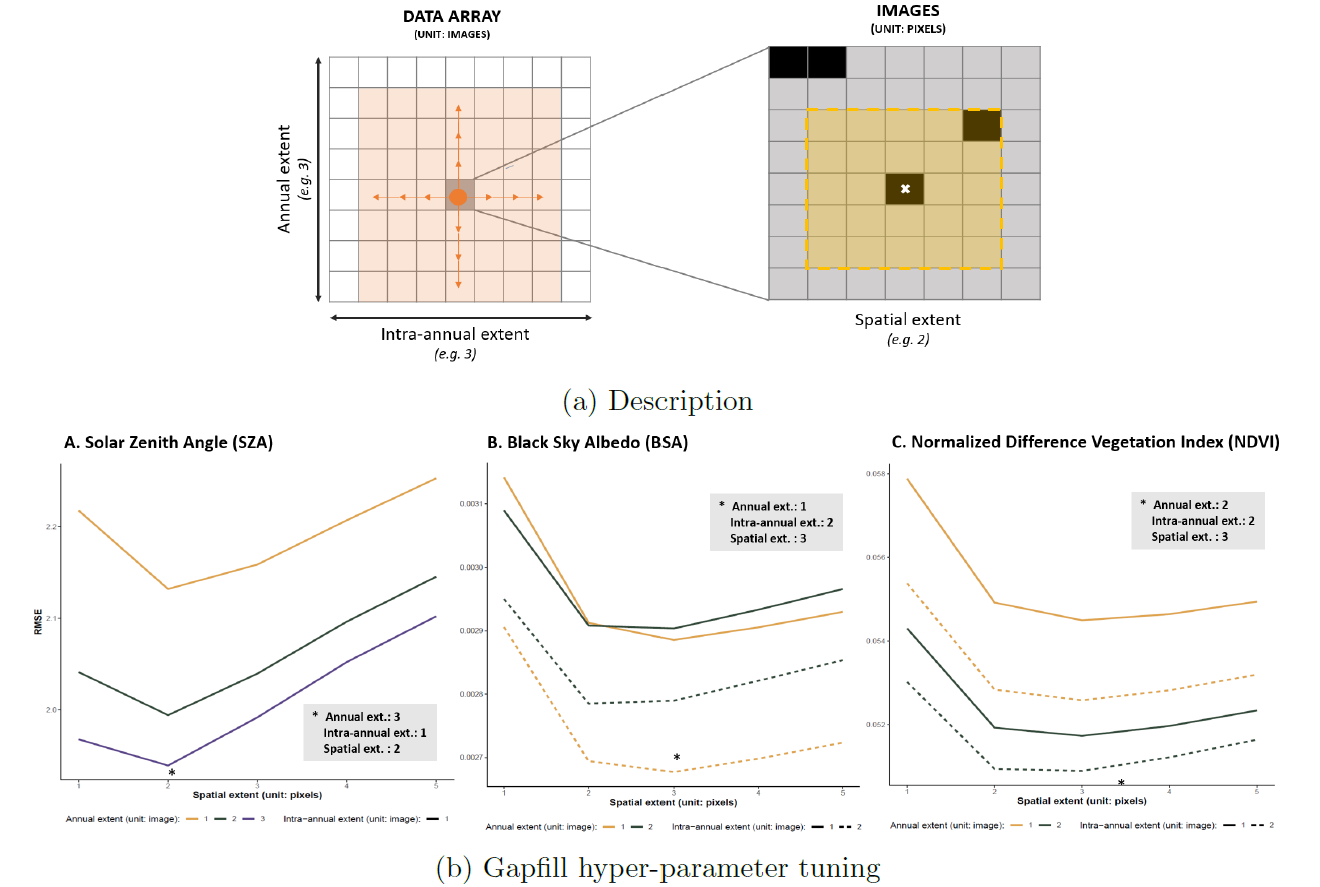


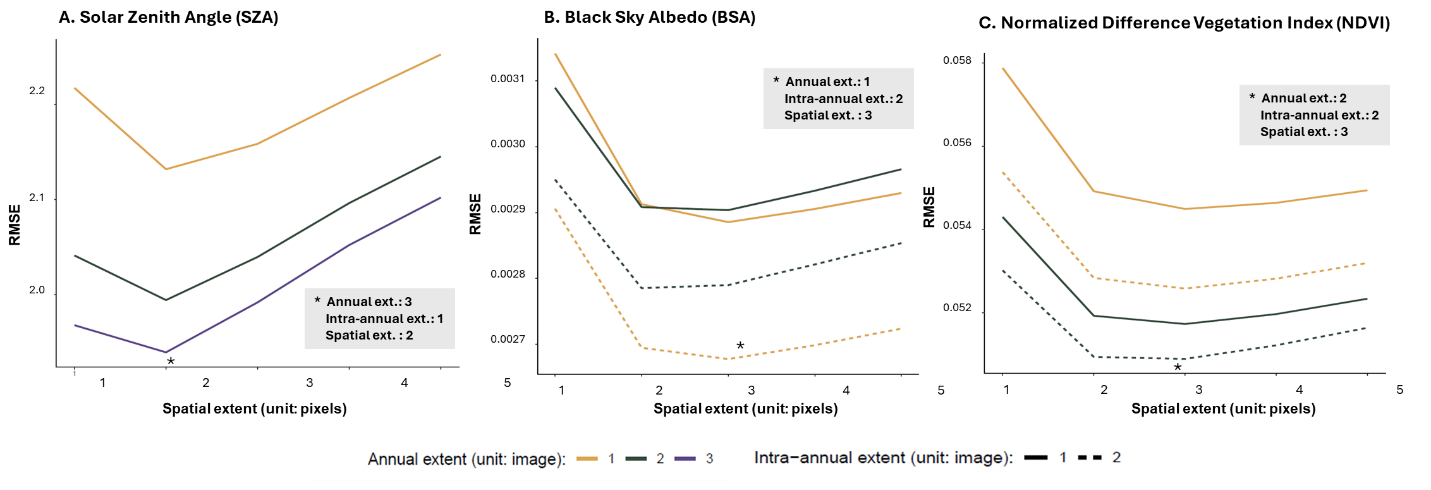


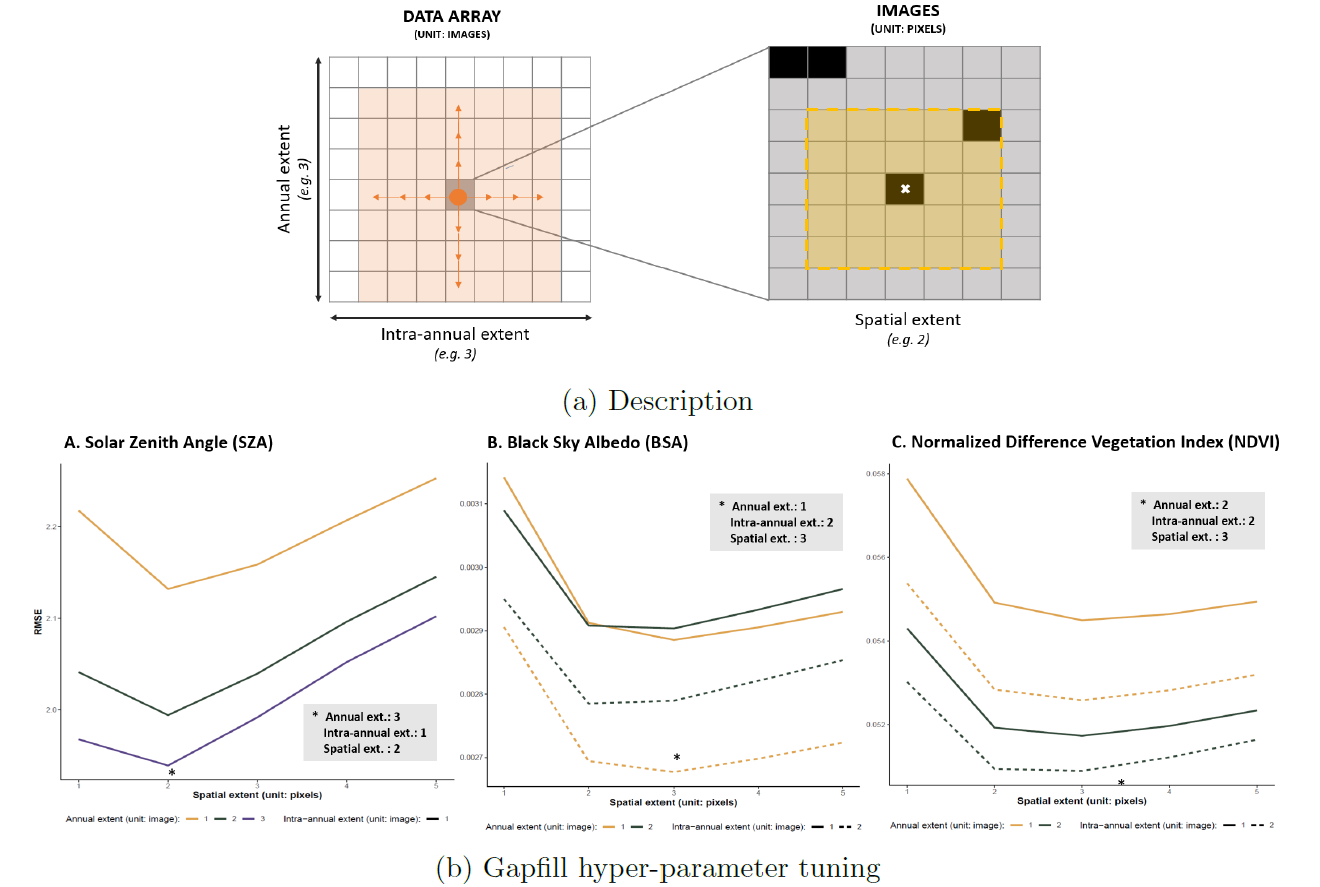


**Figure S3.2.** **Gapfill prediction window hyper-parameter tuning** (a) Scheme of the components that define a *gapfill* imputation window. (b) Results of tuning of the prediction window components using cross-validation using a random sample of 30% missing values for (a) *sza*, (b) *bsa*, and (c) *NDVI*. Windows were defined by modifying the sizes of annual (color), intra-annual (dashed or solid lines) and spatial extent (*x-axis*). RMSE values as *y-axis*. Selected combination (lowest RMSE) indicated with an asterisk (*) and in the grey box.

**
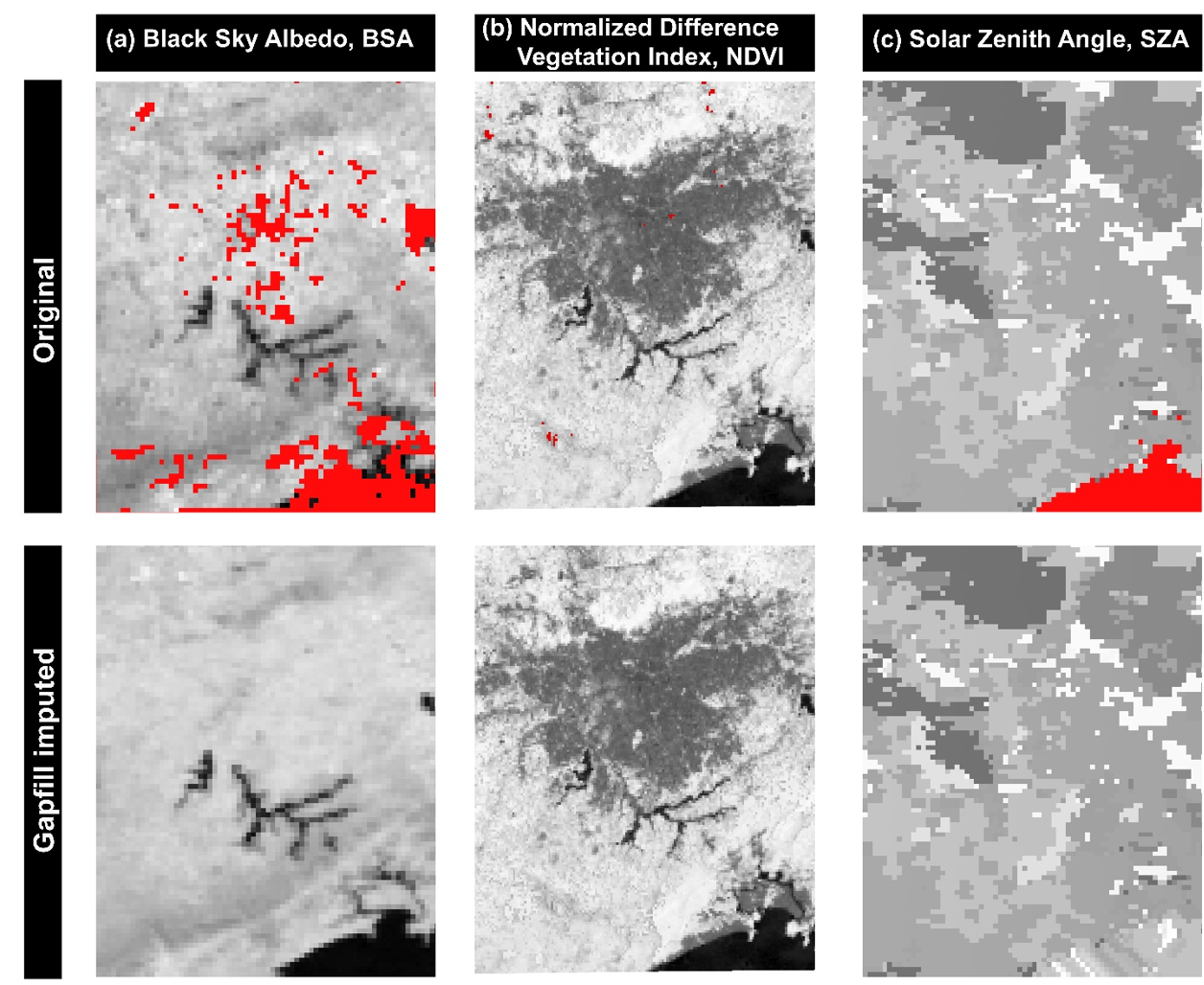
**

**Figure S3.3**: **Example of rasters imputed with** *gapfill***.** Observed (top) versus imputed (bottom) for *bsa* (left), *NDVI* (middle) and *sza* (right). In red, the pixels imputed.

## S4. Spatiotemporal predictors spatial and temporal harmonization

To harmonize the **spatial component,** we applied this methodology to our covariates to resample them all to the same resolution. For that, we used the 500 x 500m as the target raster location.

For categorical variables we used the nearest neighbourhood (NNI). For the continuous variables, we decided the resampling method based on the input/original spatial variability patterns, with two options being NNI or bilinear interpolation (BI). For those with sharp changes and extreme peaks and dips, we used nearest neighbourhood to avoid smoothing the surfaces too much and masking the extremes. For covariates with a softer spatial variability, we used the BI approach. To test that, we resampled them suing both methods and visually assessed their similarity to the original datasets, focusing on capturing spatial heterogeneity. In general, coastal areas show a smothered value for variables with estimates only for land. This adds a bias. Thus, we select NNI for those (*lst, bsa, sza*). We only saw an improvement in the ability to capture the spatial variability for Impervious surfaces, which initially was a dichotomous variable, we can see that a lot of information is lost when using NNI. Hence, we decided to use NNI interpolation for slope, elevation, land cover, population density, proximity to water and coastline, and lake variables; whilst bilinear interpolation was used for all ERA5-Land variables, impervious surfaces, land surface temperature, black sky albedo, solar zenith angle and NDVI (Table S2.1).

**Table S2.1.** Table displaying the method selected for each covariate. NBG, nearest neigbour

| **Variable** | **Name** | **Resampling method** |
| --- | --- | --- |
| slope | *slope* | NNI |
| Elevation | *dem* | NNI |
| Land cover | *landcov* | NNI |
| Population density | *popdens* | NNI |
| Proximity to water | *water* | NNI |
| Coast | *coast* | NNI |
| Lakes | *damns, b200m, b400m* | NNI |
| Impervious surfaces | *impsurf* | B |
| ESA artificial land | *artland* | BI |
| LST | *lst* | BI |
| Black Sky Albedo | *bsa* | BI |
| Solar Zenith Angle | *sza* | BI |
| NDVI | *ndvi* | BI |
| ERA5-Land data | *t2m, d2m, v10, u10, sp, rh, skt* | BI |

To harmonize the **temporal component** to daily average, we performed a cell-wise linear interpolation using each predictor’s raster stack across the study period. The number of available time slices varied based on the native temporal resolution of each dataset (see Table S1). The result was a raster stack of daily data in a 500 x 500 metres regular grid for each of our 23 predictor variables.

We used the *raster* package for the spatial and the *appraox* function for the temporal harmonization in R version 3.6.1.

## S5. Metrics for model evaluation

We used the coefficient of determination (R^2^, Eq. (A.2.)), and the root-mean-square error (RMSE, Eq. (A.3.)), as measures of accuracy of the predictions to evaluate and compare the models. See below for their mathematical expressions:

| $R^{2}=1- \frac{\sum_{j=1}^{m} {(\hat{T}\left( s_{j},t_{j} \right)-T\left( s_{j},t_{j} \right))}^{2}}{\sum_{j=1}^{m} {(T\left( s_{j},t_{j} \right)-\bar{T})}^{2}}$ | Eq. (A.2.) |
| --- | --- |
| $RMSE=\sqrt{\frac{\sum_{j=1}^{m} {[\hat{T}\left( s_{j},t_{j} \right)-T\left( s_{j},t_{j} \right)]}^{2}}{m}}$ | Eq. (A.3.) |

In Equations (A.2) and (A.3), we use a single index j = 1, …, m to label each spatiotemporal observation in the evaluation set. Where *T(s_j,_ t_j_)* is the observed temperature at site *s_j_* on day *t_j_*; $\hat{T}$(*s_j_, t_j_*) is the corresponding model prediction; and $\bar{T}$is the mean observed temperature across all these observations. Here, *m* is the total number of observations included in the evaluation (that is, all site-day pairs in the validation/test set). Finally, to test the results for bias, we followed [7] approach, and estimated the temporal and spatial error associated to the model measured through a regression (R^2^). The temporal error was calculated by regressing the difference between the observed temperature at time *t* and space *s*, and the annual mean temperature observed, against the difference between the predicted temperature at time t and space s, and the annual mean temperature predicted. The spatial error was calculated by regressing the station-specific annual mean estimates in observed temperatures against the station-specific annual means from the predicted temperature. The algebraic notation is as follows:

| $y=\beta_{0}+\beta_{1}x_{i}+e_{i}$ | Eq. (A.4.) |
| --- | --- |
| $R^{2}=1-\frac{\sum{(y_{i}-\hat{y}_{i})}^{2}}{\sum{(y_{i}-y)}^{2}}$ | Eq. (A.5.) |

Where $i$ represents an observation in the total number of observations considered, and $y_{i}$ and $x_{i}$ vary depending on the error being estimated. For temporal error they are:

| $y_{i}=\Delta\left( T \right)={T(s,t)}_{l}-\frac{\sum^{n} {T(s,t)}_{l}}{n}$ | Eq. (A.6.) |
| --- | --- |
| $x_{i}=\Delta\left( \hat{T} \right)=\hat{T}\left( s,t \right)-\frac{\sum^{n} \hat{T}(s,t)}{n}$ | Eq. (A.7.) |

and for the spatial errors they are:

| $y_{l}=\frac{\sum^{n} {T(s,t)}_{l}}{n}$ | Eq. (A.8.) |
| --- | --- |
| $x_{l}=\frac{\sum^{n} {\hat{T}(s,t)}_{l}}{n}$ | Eq. (A.9.) |

where $T(s,t)$ and $\hat{T}(s,t)$ represent the temperature at time $t$ and space $s$ observed and predicted, respectively, and $n$ is the total number of observations within a year$l$.

## S6. Multi-linear regression model (MLR)

In exposure science, multi-linear regression models are a popular approach to explain and predict spatial and temporal variability of environmental variables. Among one of the advantages of regression approaches over RF is their capacity to extrapolate outside of the training range. This is of particular interest for applications where extreme values are important. Therefore, we used this comparison to assess whether the RF model was substantially underestimating extreme values. The model assumed the following general form:


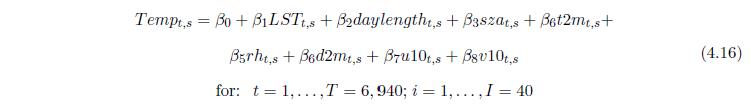


where *Tempt,s* is the predicted temperature at time t and location s, β0 is the intercept, β1−8 are the linear coefficients describing the association between temperature and the selected covariates, namely land surface temperature (*LSTt,s*), day length (*daylengtht,s*), solar zenith angle(*szat,s*), dew point and temperature at 2m (*d2mt,s* and *t2mt,s*), and wind components *(v10t,s* and *u10t,s*), respectively. The features included in the model where those selected for the RF approach. Forward feature selection on a vanilla RF is not only a common approach to select features for multi-linear regression models, but in our case, it also ensures comparability with the RF model.

We assessed model assumptions and outlier influence by examining a histogram of regression standardized residuals, a normal Q-Q plot, and a scatter plot of residuals against fitted values (Figure S5.1). The histogram shows a bell-shaped distribution, indicating normality in residuals. While the Q-Q plot suggests some deviation from normal at the extremes, it is expected. Additionally, the absence of a systematic pattern in residuals across fitted values supports linear assumption, equal error variances, and the absence of problematic outliers. The model coefficients are shown in Figure S5.2.


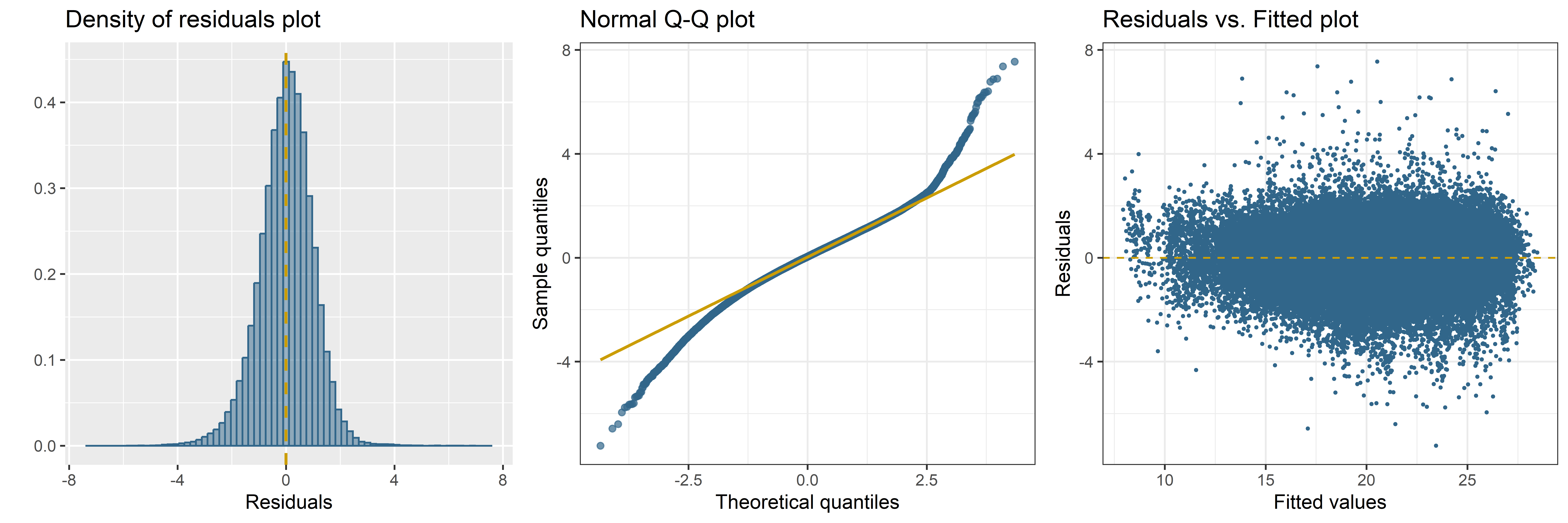


**Figure S5.1**: LUR model residual diagnostic plots. (Left) Density plot of the model residuals distribution. (Center) The Q-Q plot showing the empirically observed quantiles of model residuals (y-axis) as a function of the quantiles expected from a normal distribution with the same mean and variance as the empirical distribution (x-axis). (Right) Scatter plot of the model residuals plotted against the fitted values.


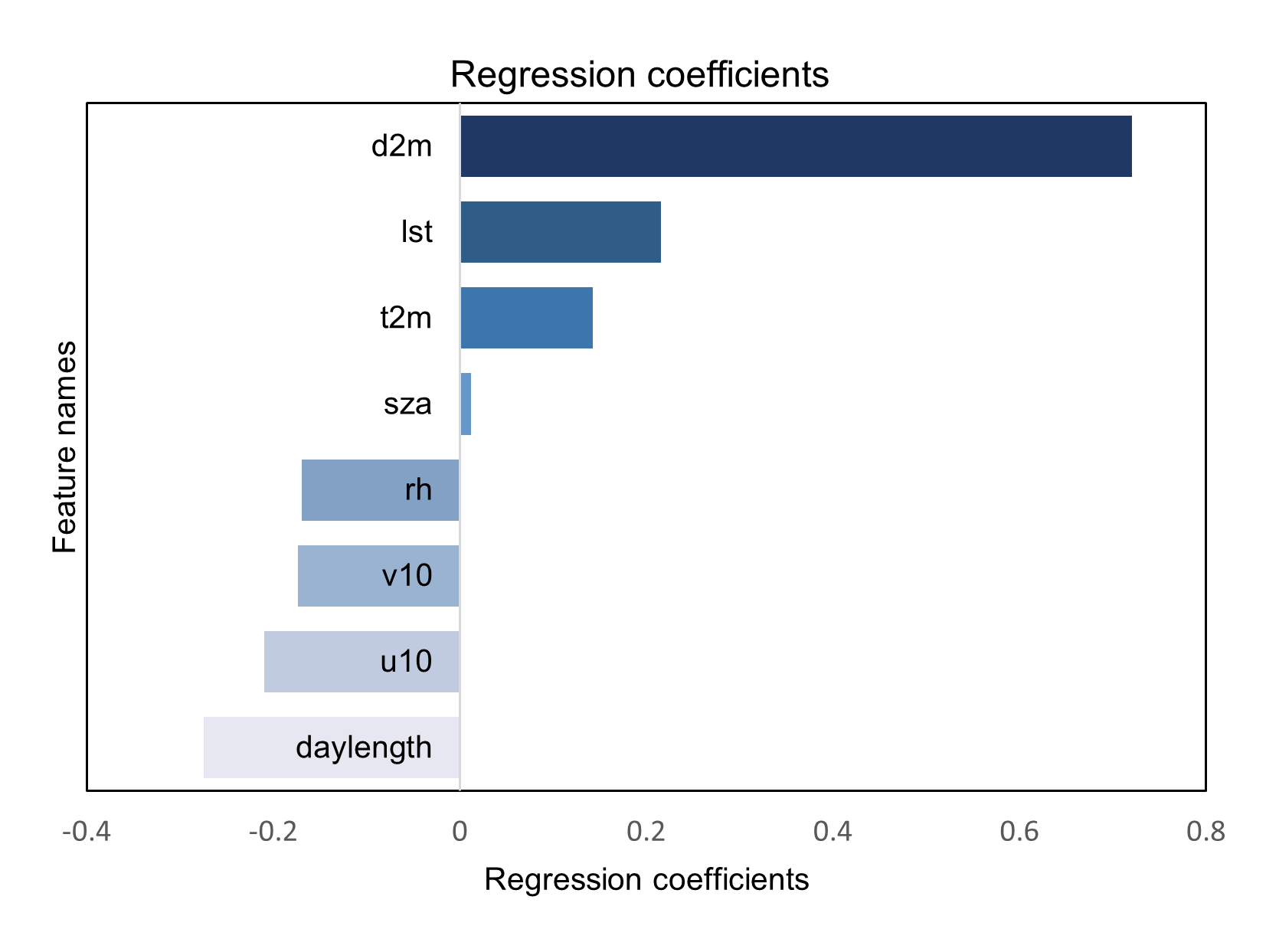


**Figure S5.2.** Regression standardized coefficients for the multi-linear regression model (MLR).

## References

1. Armstrong, B. *et al.* The role of humidity in associations of high temperature with mortality: A multicountry, multicity study. *Environmental Health Perspectives* 127, e097007 (2019).

2. MacQueen, J. B. J. and others. Some methods for classification and analysis of multivariate observations. *Proceedings of the fifth Berkeley symposium on mathematical statistics and probability* 1, 281–297 (1967).

3. Shiff, S., Helman, D. & Lensky, I. M. Worldwide continuous gap-filled MODIS land surface temperature dataset. *Sci Data* 8, 1–10 (2021).

4. Gerber, F., De Jong, R., Schaepman, M. E., Schaepman-Strub, G. & Furrer, R. Predicting Missing Values in Spatio-Temporal Remote Sensing Data. *IEEE Transactions on Geoscience and Remote Sensing* 56, 2841–2853 (2018).

5. Saha, S. *et al.* NCEP Climate Forecast System Version 2 (CFSv2) 6-hourly Products. Preprint at (2011).

6. Gerber, F. Package ’gapfill’ : Fill Missing Values in Satellite Data. Preprint at https://doi.org/10.1109/TGRS.2017.2785240 (2022).

7. Kloog, I., Nordio, F., Coull, B. A. & Schwartz, J. Predicting spatiotemporal mean air temperature using MODIS satellite surface temperature measurements across the Northeastern USA. *Remote Sens Environ* 150, 132–139 (2014).

# Tables

**Table S1. Candidate input features**. List of input features considered for inclusion in the Random Forest model with information the data product names, original and post-processing temporal and spatial resolution, percentage of missing values (NAs), imputation method used if any, temporal coverage of the data product, and data source reference. S: spatial, ST: spatiotemporal

| **Type** | **Overall group** | **Variable** | **Name** | **Product name** | **Missing (% all period)** | **Imputation method** | **Validation Imputation RMSE** | **Original Spatial Resolution** | **Original Temporal Resolution** | **Temporal coverage** |
| --- | --- | --- | --- | --- | --- | --- | --- | --- | --- | --- |
| **S** | Anthropogenic heat proxies | Impervious surfaces | *impsurf* | [Xiao Zhang et al 2020 Impervious surfaces global dataset](https://essd.copernicus.org/articles/12/1625/2020/#section2) | 0% | - | - | 30 x30m | Annual (2015) | 2015 |
| **S** | Terrain | Elevation | *dem* | SRTMGL1 v003 | 0% | - | - | 500 x 500m | Annual (2015) | 2015 |
| **S** | Terrain | Slope (Inclination) | *slope* | SRTMGL1 v003 | 0% | - | - | 500 x 500m | Annual (2015) | 2015 |
| **S** | Terrain | Land cover class | *landcov* | ESACCI-LC: European Space Agency (ESA) Climate Change Initaitive (CCI) Land Cover product (LC) | 0% | - | - | 300 x 300 | Annual (2015) | 2015 |
| **S** | Green and blue spaces | Distance to coastline | *coast* | *Estimated* | 0% | *-* | *-* | shapefile with coast line | Annual (2004) | 2004 |
| **S** | Green and blue spaces | Distance to water bodies | *water* | *WorldPop* | 0% | *-* | *-* | 100 x 100m | Period (2000-2012) | Period (2000-2012) |
| **S** | Green and blue spaces | Distance to damns (damns boundary) | *damns* | *Estimated* | 0% | *-* | *-* | shapefile with lakes profiles | Annual (2004) | 2004 |
| **S** | Green and blue spaces | Distance to damns (200m buffer) | *b200m* | *Estimated* | 0% | *-* | *-* | shapefile with lakes profiles | Annual (2004) | 2004 |
| **S** | Green and blue spaces | Distance to damns (400m buffer) | *b400m* | *Estimated* | 0% | *-* | *-* | shapefile with lakes profiles | Annual (2004) | 2004 |
| **ST** | Green and blue spaces | Normalized difference vegetation index (NDVI) | *ndvi* | USGS Landsat 7 Collection 1 Tier 1 TOA Reflectance | 5.05% | Gapfill interpolation by Gerber et al. 2018 | 0.05 | 500 x 500m | Daily | 2015-2019 |
| **ST** | Anthropogenic heat proxies | Distance to aritficial land cover | *artland* | WorldPop | 0% | - | - | 100 x 100m | Annual (2015) | 2015 |
| **ST** | Anthropogenic heat proxies | Population density | *popdens* | WorldPop | 0% | - | - | 1 x 1km | Annual (2015) | 2015 |
| **ST** | Atmospheric variables | Black Sky Albedo | *bsa* | MCD43A3_v061 | 13.00% | Gapfill interpolation by Gerber et al. 2018 | 0.01 | 1 x 1km | Daily (based on 16-day average) | 2015-2019 |
| **ST** | Atmospheric variables | Land surface Temperature | *lst* | Continuous LST by Shiff et al. 2021 (input: MOD11A1_v006 + Climate Forecast System Version 2 (CFSv2)) | 31.5% | Continuous LST dataset developed by Shiff et al. 2021 | 2.34 | 1 x 1km | 3 times per day | 2015-2019 |
| **ST** | Atmospheric variables | Solar Zenith Angle | *sza* | MOD13A2_v006 | 4.67% | Gapfill interpolation by Gerber et al. 2018 | 1.94 | 1 x 1km | 16-day average | 2015-2019 |
| **ST** | Weather | Temperature at 2m; ERA5-Land | *t2m* | ERA-5 Land | 0% | - | - | ~9 x 9km | Daily | 2015-2019 |
| **ST** | Weather | Wind speed; ERA5-Land | *v10* | ERA-5 Land | 0% | - | - | ~9 x 9km | Daily | 2015-2019 |
| **ST** | Weather | Wind direction; ERA5-Land | *u10* | ERA-5 Land | 0% | - | - | ~9 x 9km | Daily | 2015-2019 |
| **ST** | Weather | Pressure; ERA5-Land | *sp* | ERA-5 Land | 0% | - | - | ~9 x 9km | Daily | 2015-2019 |
| **ST** | Weather | Skin temperature; ERA5-Land | *skt* | ERA-5 Land | 0% | - | - | ~9 x 9km | Daily | 2015-2019 |
| **ST** | Weather | Relative humidity; derived from ERA5-Land | *rh* | ERA-5 Land | 0% | - | - | ~9 x 9km | Daily | 2015-2019 |
| **ST** | Weather | Dew point 2m temperature; ERA5-Land | *d2m* | ERA-5 Land | 0% | - | - | ~9 x 9km | Daily | 2015-2019 |
| **T** | Temporal variables | Day length | *daylength* | *Estimated* | 0% | - | - | - | Daily | 2015-2019 |
|  |  |  |  |  |  |  |  |  |  |  |

**Table S2:** Hyper-parameter definitions. Definition of the tuned parameters according to *Scikitlearn RandomForestRegressor* package. In bold, the values selected by hyper-parameter tuning.

**
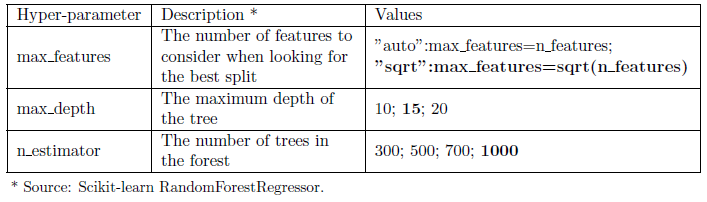
**

**Table S3.** Descriptive statistics (minimum, 1st quartile, median, mean, 3^rd^ quartile, and maximum) for observed temperature data and input features at locations intersecting the stations included in the model.

| **Variables** | **Min.** | **1st Qu.** | **Median** | **Mean** | **3rd Qu.** | **Max.** |
| --- | --- | --- | --- | --- | --- | --- |
| *Temperature*  *(observed)* | 6.06 | 17.88 | 20.52 | 20.29 | 22.86 | 32.82 |
| *bsa* | 0.07 | 0.12 | 0.13 | 0.13 | 0.13 | 0.18 |
| *sza* | 13.60 | 26.87 | 34.85 | 36.20 | 45.74 | 56.39 |
| *t2m* | 7.34 | 17.57 | 20.03 | 19.80 | 22.19 | 29.28 |
| *d2m* | 3.18 | 13.87 | 16.72 | 16.28 | 19.03 | 25.36 |
| *ndvi* | 0.08 | 0.16 | 0.20 | 0.26 | 0.33 | 0.75 |
| *lst* | 8.40 | 22.03 | 25.08 | 24.86 | 27.92 | 38.47 |
| *coast* | 11.54 | 46.14 | 52.40 | 52.07 | 59.90 | 79.97 |
| *popdens* | 0.04 | 4183.70 | 8497.88 | 8398.40 | 12567.32 | 25703.31 |
| *impsurf* | 1.00 | 1.58 | 1.90 | 1.74 | 1.96 | 2.00 |
| *landcov* | 50.00 | 174.40 | 190.00 | 165.46 | 190.00 | 210.00 |
| *artland* | -3.04 | -1.42 | -0.82 | -0.57 | 0.04 | 4.90 |
| *lake* | 0.00 | 0.00 | 0.00 | 1.16 | 0.00 | 34.25 |
| *b200m* | 0.00 | 0.00 | 0.00 | 1.27 | 0.00 | 29.09 |
| *b400m* | 0.00 | 0.00 | 0.00 | 1.44 | 0.00 | 24.11 |
| *dem* | 370.85 | 747.49 | 761.91 | 760.47 | 782.41 | 991.41 |
| *slope* | 0.20 | 1.11 | 1.51 | 1.67 | 1.68 | 9.47 |
| *water* | 0.00 | 0.42 | 1.00 | 1.14 | 1.82 | 4.17 |
| *v10* | -5.04 | -0.79 | 0.33 | 0.23 | 1.32 | 4.55 |
| *u10* | -4.56 | -1.54 | -0.67 | -0.58 | 0.34 | 5.24 |
| *sp* | 91451.05 | 92755.57 | 93076.37 | 93165.11 | 93397.81 | 99088.97 |
| *skt* | 280.77 | 291.51 | 294.33 | 294.06 | 296.79 | 304.97 |
| *rh* | 33.42 | 76.16 | 82.39 | 80.65 | 86.78 | 98.66 |
| *rh* | 0.00 | 72.94 | 80.29 | 79.61 | 87.42 | 100.00 |
| *daylength* | 10.65 | 11.12 | 12.08 | 12.10 | 13.08 | 13.62 |

**Table S4.** Permutation-based feature importance scores for RF, representing average across ten runs.

| **Predictors** | **Feature Importance** | **Standard Deviation** |
| --- | --- | --- |
| ***t2m*** | 0.492 | 0.004 |
| ***lst*** | 0.144 | 0.002 |
| ***d2m*** | 0.068 | 0.001 |
| ***rh*** | 0.052 | 0.001 |
| ***v10*** | 0.028 | 0.001 |
| ***daylength*** | 0.023 | 0.000 |
| ***sza*** | 0.014 | 0.000 |
| ***u10*** | 0.011 | 0.000 |

**Table S5.** Accuracy scores (RMSE in ⁰C, and R^2^) for the RF and MLR models for the whole study period and by meteorological station network, year, month and urbanicity, assessed through station-based CV.

|  | **Random Forest (RF)** | | **Multi-Linear Regression (MLR)** | |
| --- | --- | --- | --- | --- |
| **Station** | **RMSE** | **R^2^** | **RMSE** | **R^2^** |
| Overall | 0.80 | 0.95 | 1.02 | 0.92 |
| 2015 | 0.80 | 0.94 | 1.04 | 0.90 |
| 2016 | 0.79 | 0.96 | 1.04 | 0.93 |
| 2017 | 0.78 | 0.94 | 1.03 | 0.91 |
| 2018 | 0.84 | 0.94 | 0.99 | 0.91 |
| 2019 | 0.81 | 0.95 | 0.99 | 0.92 |
| Jan | 0.74 | 0.89 | 0.94 | 0.82 |
| Feb | 0.74 | 0.89 | 0.92 | 0.83 |
| Mar | 0.69 | 0.86 | 0.84 | 0.80 |
| Apr | 0.69 | 0.93 | 0.87 | 0.89 |
| May | 0.75 | 0.89 | 0.92 | 0.84 |
| Jun | 0.84 | 0.91 | 1.08 | 0.85 |
| Jul | 0.94 | 0.90 | 1.09 | 0.86 |
| Aug | 0.86 | 0.92 | 1.11 | 0.87 |
| Sep | 0.94 | 0.93 | 1.30 | 0.89 |
| Oct | 0.85 | 0.92 | 1.09 | 0.88 |
| Nov | 0.76 | 0.91 | 0.95 | 0.87 |
| Dec | 0.75 | 0.90 | 0.95 | 0.86 |
| Rural | 1.05 | 0.91 | 1.15 | 0.89 |
| Urban | 0.76 | 0.95 | 1.00 | 0.92 |

**Table S6.** External validation performance scores for RF and MLR. Performance scores (RMSE in ⁰C, and R^2^) calculated using the hold-out dataset consisting of 5 randomly selected stations. Results presented overall and for each station.

|  | **Random Forest (RF)** | | **Multi-Linear Regression (MLR)** | |
| --- | --- | --- | --- | --- |
| **Station** | **RMSE** | **R^2^** | **RMSE** | **R^2^** |
| *1000840* | 0.47 | 0.98 | 0.62 | 0.95 |
| *1000944* | 0.66 | 0.98 | 0.80 | 0.94 |
| *A744* | 1.34 | 0.81 | 1.14 | 0.79 |
| *CETESB_12* | 0.90 | 0.98 | 1.03 | 0.94 |
| *CETESB_19* | 1.74 | 0.86 | 1.48 | 0.88 |
| **Overall** | **1.00** | **0.92** | **1.20** | **0.89** |

**Table S7.** Model performance and accuracy by sensitivity analysis. Performance scores (RMSE in ^◦^C, and R^2^) for all sensitivity analyses for the whole study periods and by meteorological station network, year, month and urbanicity, assessed through station-based CV. MM, main model; SA_coast_, MM adding variable *coast*; SA_d2m_, MM removing variable *d2m*, and SA_d2m_, MM adding *coast* but removing *d2m*

|  | **MM** | | **SA_coast_** | | **SA_d2m_** | | **SA_both_** | |
| --- | --- | --- | --- | --- | --- | --- | --- | --- |
| **Group** | **RMSE** | **R^2^** | **RMSE** | **R^2^** | **RMSE** | **R^2^** | **RMSE** | **R^2^** |
| Total | 0.803 | 0.947 | 0.803 | 0.947 | 0.802 | 0.947 | 0.802 | 0.947 |
| airport_stations | 0.738 | 0.96 | 0.738 | 0.96 | 0.739 | 0.96 | 0.739 | 0.96 |
| cetesb_stations | 0.728 | 0.955 | 0.729 | 0.955 | 0.729 | 0.955 | 0.728 | 0.955 |
| cgs_stations | 0.837 | 0.944 | 0.837 | 0.944 | 0.836 | 0.944 | 0.836 | 0.944 |
| GHCN_NOAA_stations | 0.432 | 0.985 | 0.433 | 0.985 | 0.432 | 0.985 | 0.434 | 0.985 |
| gss_noaa_stations | 0.798 | 0.951 | 0.798 | 0.951 | 0.797 | 0.951 | 0.797 | 0.951 |
| INMET | 0.762 | 0.95 | 0.762 | 0.95 | 0.761 | 0.95 | 0.761 | 0.95 |
| USP_station | 0.626 | 0.968 | 0.627 | 0.967 | 0.628 | 0.967 | 0.628 | 0.967 |
| 2015 | 0.798 | 0.939 | 0.798 | 0.939 | 0.798 | 0.939 | 0.797 | 0.939 |
| 2016 | 0.788 | 0.96 | 0.788 | 0.96 | 0.788 | 0.96 | 0.788 | 0.96 |
| 2017 | 0.779 | 0.945 | 0.78 | 0.945 | 0.779 | 0.945 | 0.78 | 0.945 |
| 2018 | 0.837 | 0.935 | 0.837 | 0.935 | 0.836 | 0.935 | 0.836 | 0.935 |
| 2019 | 0.807 | 0.95 | 0.807 | 0.95 | 0.806 | 0.95 | 0.806 | 0.95 |
| Jan | 0.74 | 0.893 | 0.739 | 0.893 | 0.739 | 0.893 | 0.738 | 0.893 |
| Feb | 0.741 | 0.888 | 0.74 | 0.888 | 0.74 | 0.888 | 0.741 | 0.888 |
| Mar | 0.694 | 0.863 | 0.693 | 0.863 | 0.693 | 0.863 | 0.692 | 0.863 |
| Apr | 0.694 | 0.929 | 0.695 | 0.929 | 0.695 | 0.929 | 0.694 | 0.929 |
| May | 0.749 | 0.89 | 0.75 | 0.889 | 0.749 | 0.89 | 0.75 | 0.889 |
| Jun | 0.843 | 0.908 | 0.843 | 0.908 | 0.843 | 0.908 | 0.842 | 0.908 |
| Jul | 0.936 | 0.899 | 0.937 | 0.899 | 0.935 | 0.899 | 0.937 | 0.899 |
| Aug | 0.856 | 0.922 | 0.856 | 0.922 | 0.856 | 0.922 | 0.857 | 0.922 |
| Sep | 0.944 | 0.93 | 0.945 | 0.929 | 0.944 | 0.93 | 0.944 | 0.93 |
| Oct | 0.855 | 0.922 | 0.856 | 0.922 | 0.855 | 0.922 | 0.855 | 0.922 |
| Nov | 0.764 | 0.911 | 0.765 | 0.911 | 0.764 | 0.911 | 0.765 | 0.911 |
| Dec | 0.75 | 0.901 | 0.75 | 0.902 | 0.748 | 0.902 | 0.749 | 0.902 |
| Rural | 1.053 | 0.909 | 1.053 | 0.909 | 1.051 | 0.909 | 1.052 | 0.909 |
| Urban | 0.765 | 0.952 | 0.765 | 0.952 | 0.765 | 0.952 | 0.765 | 0.952 |
| RMSE and R^2^ values with three decimal places to allow to see differences across Sensitivity Analyses. | | | | | | |  |  |

# Figures

**
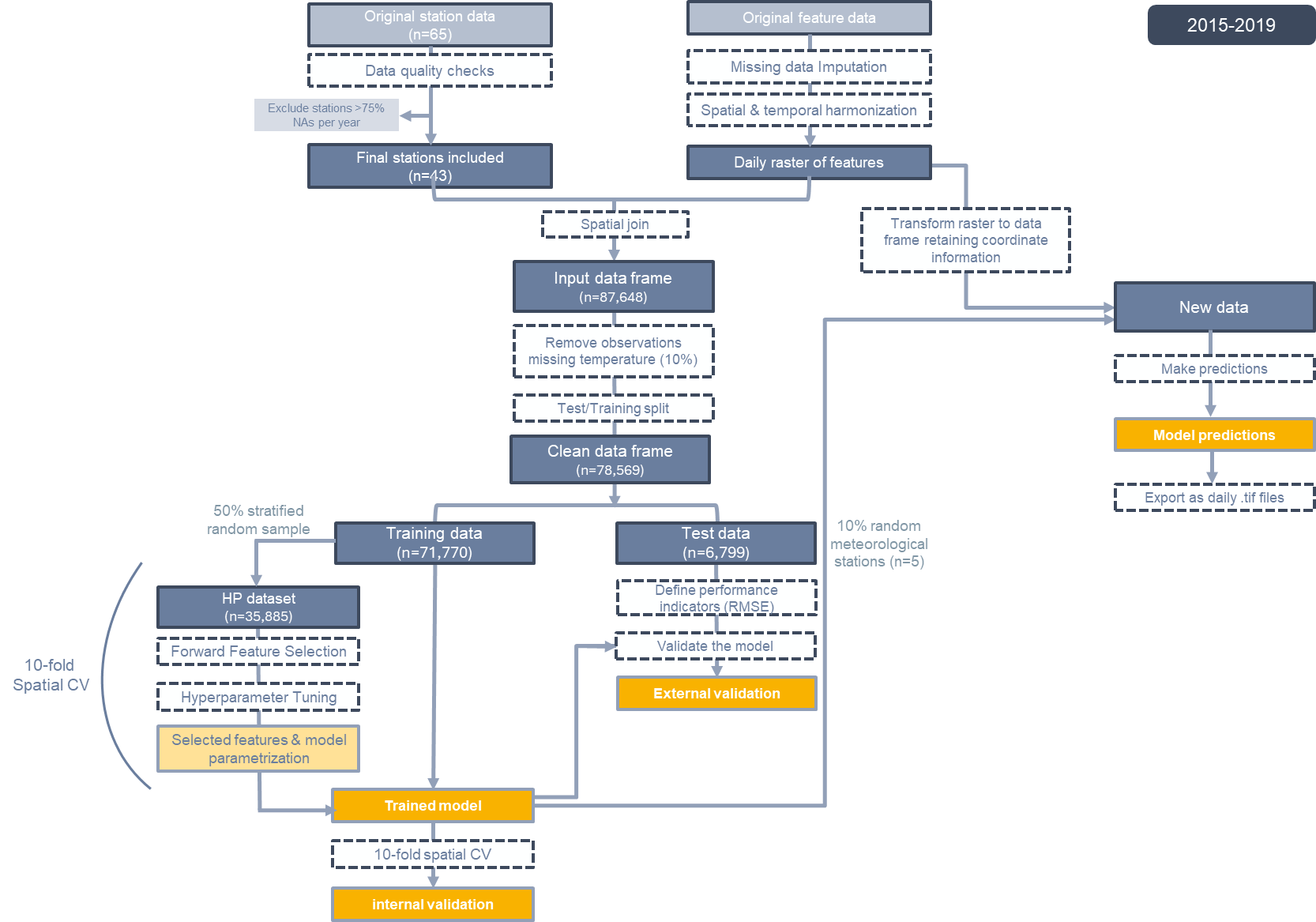
**

**Figure S1:** Model workflow. Diagram of the workflow followed, including data pre-processing, model training and predictions.

**
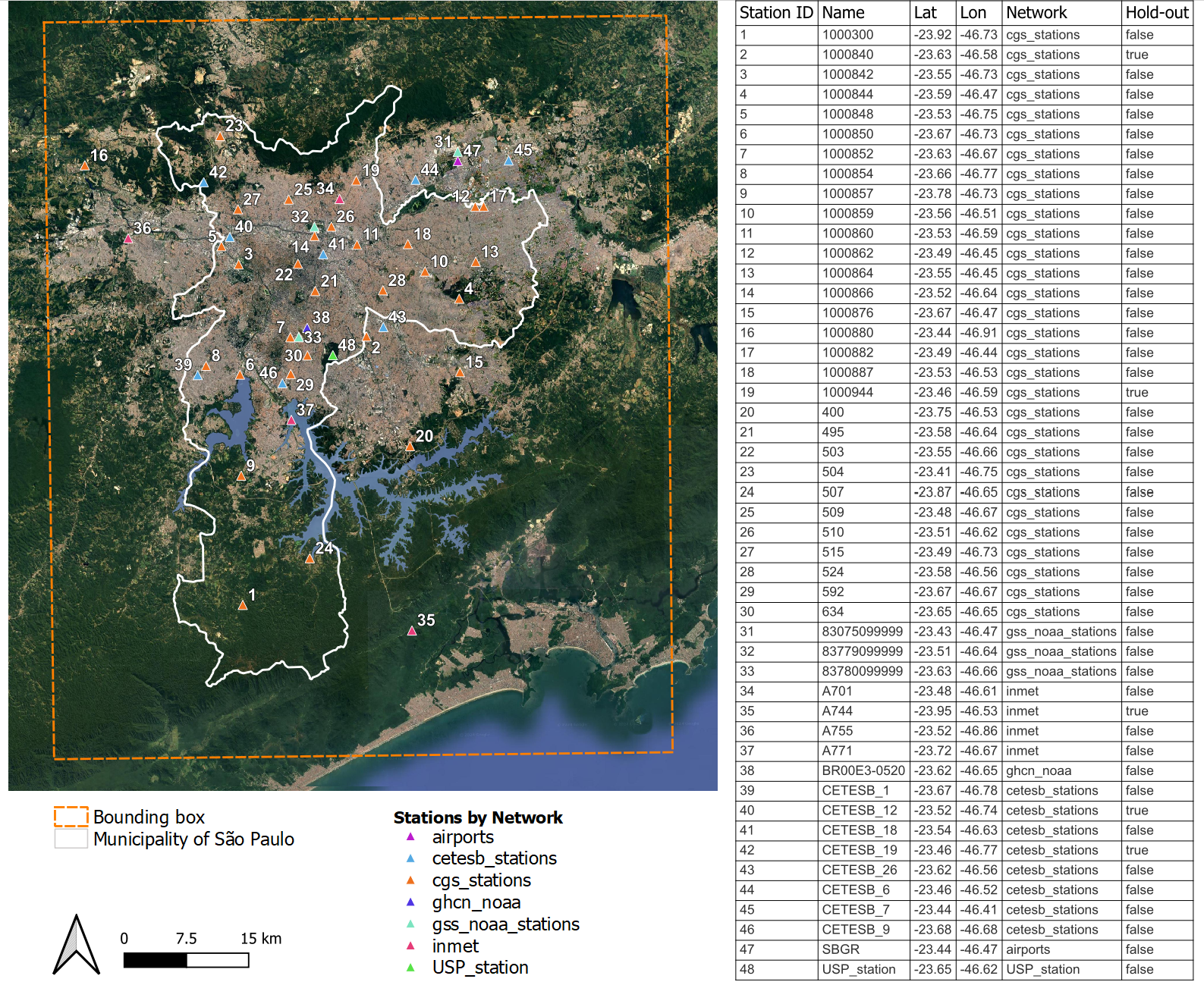
**

**Figure S2.** Distribution of included meteorological stations (n=48) coloured by network. Numbers correspond to the names of the stations in the table. CETESB, *Companhia Ambiental do Estado de São Paulo;* GCS, Global Climate Station; GHCN, Global Historical Climatology Network;  GSS, Geostationary Satellite Server; INMET, *Instituto Nacional de METeorologia*; NOAA, National Oceanic and Atmospheric Administration, and USP, *Universidade de São Paulo.*


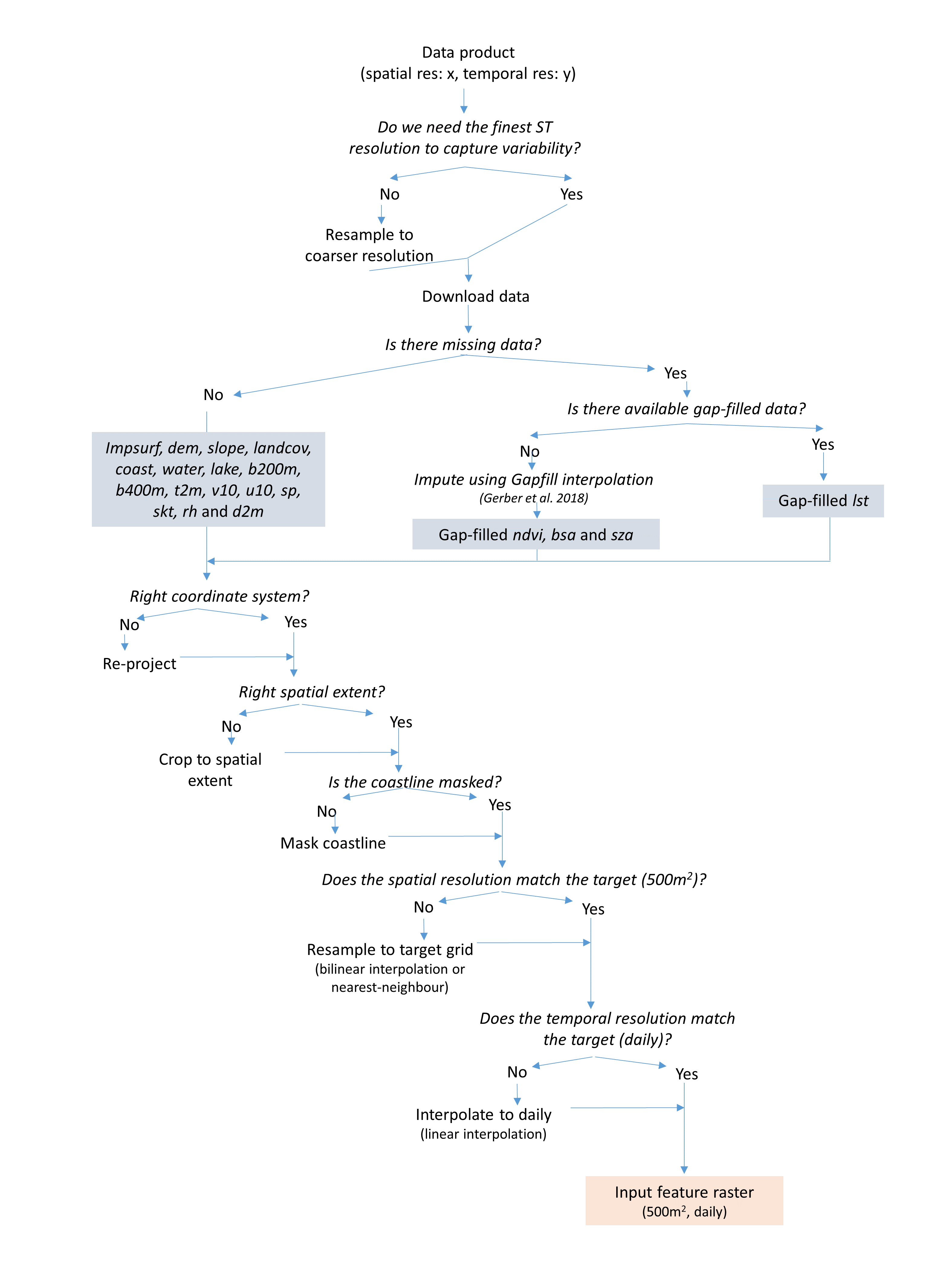


**Figure S3.** Visual representation of the workflow followed to assess the need for data imputation and spatial and temporal harmonization of all considered input features.

**
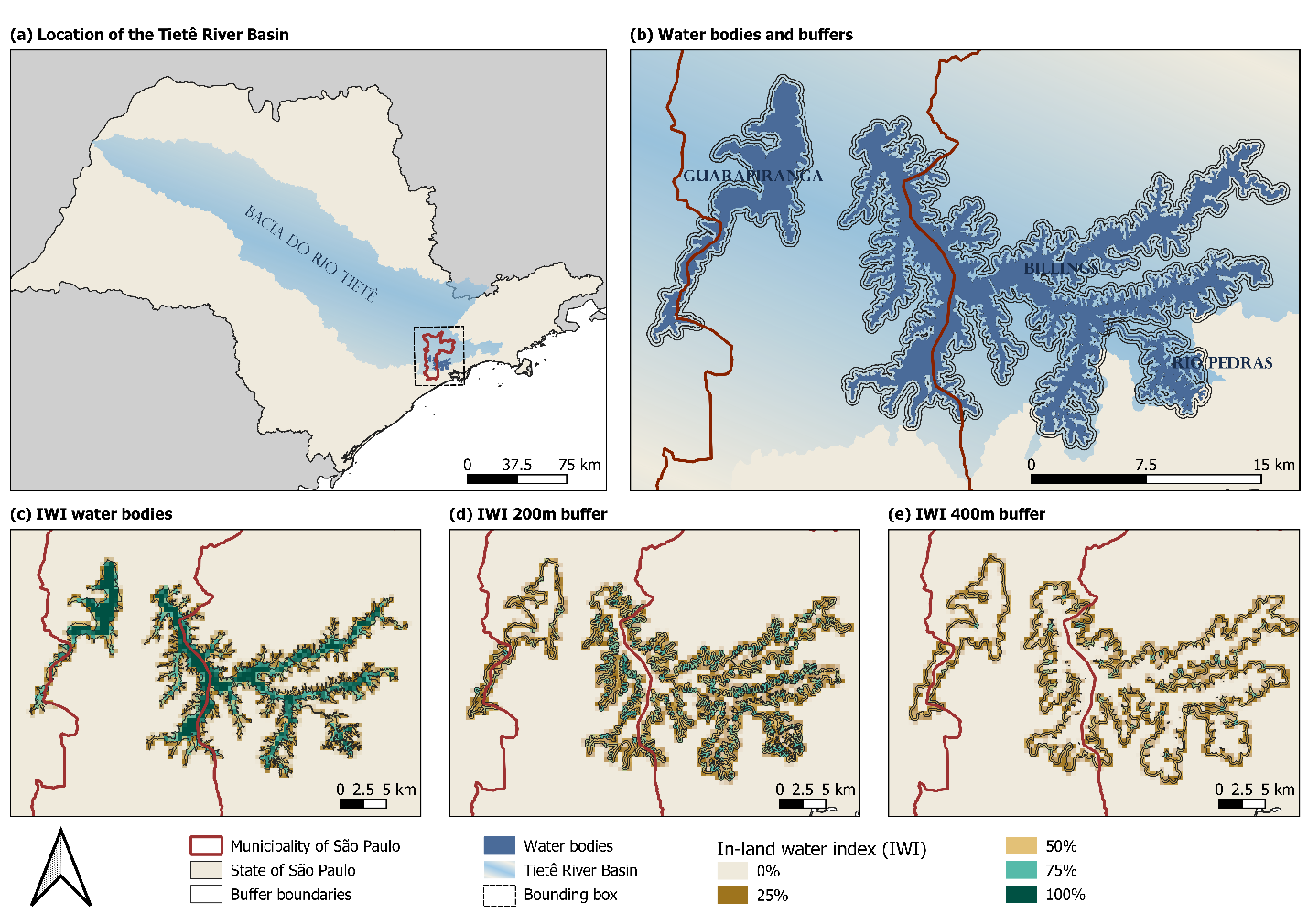
**

**Figure S4.** (Top, a) In light blue, the *Tiete* river basin expanding over the State of São Paulo. (b) Close up view of the three major inland water bodies; the *Guarapiranga*, the *Billings* and *Rio Pedr*as lake/damn in dark blue. The 200m and 400m distance buffers are shown in black. (Bottom) Maps showing the percentage of area per grid cell (or *in-land water index*, IWI) intersecting (c) the water bodies, (d) the 200m buffer, and (e) the 400m buffer.


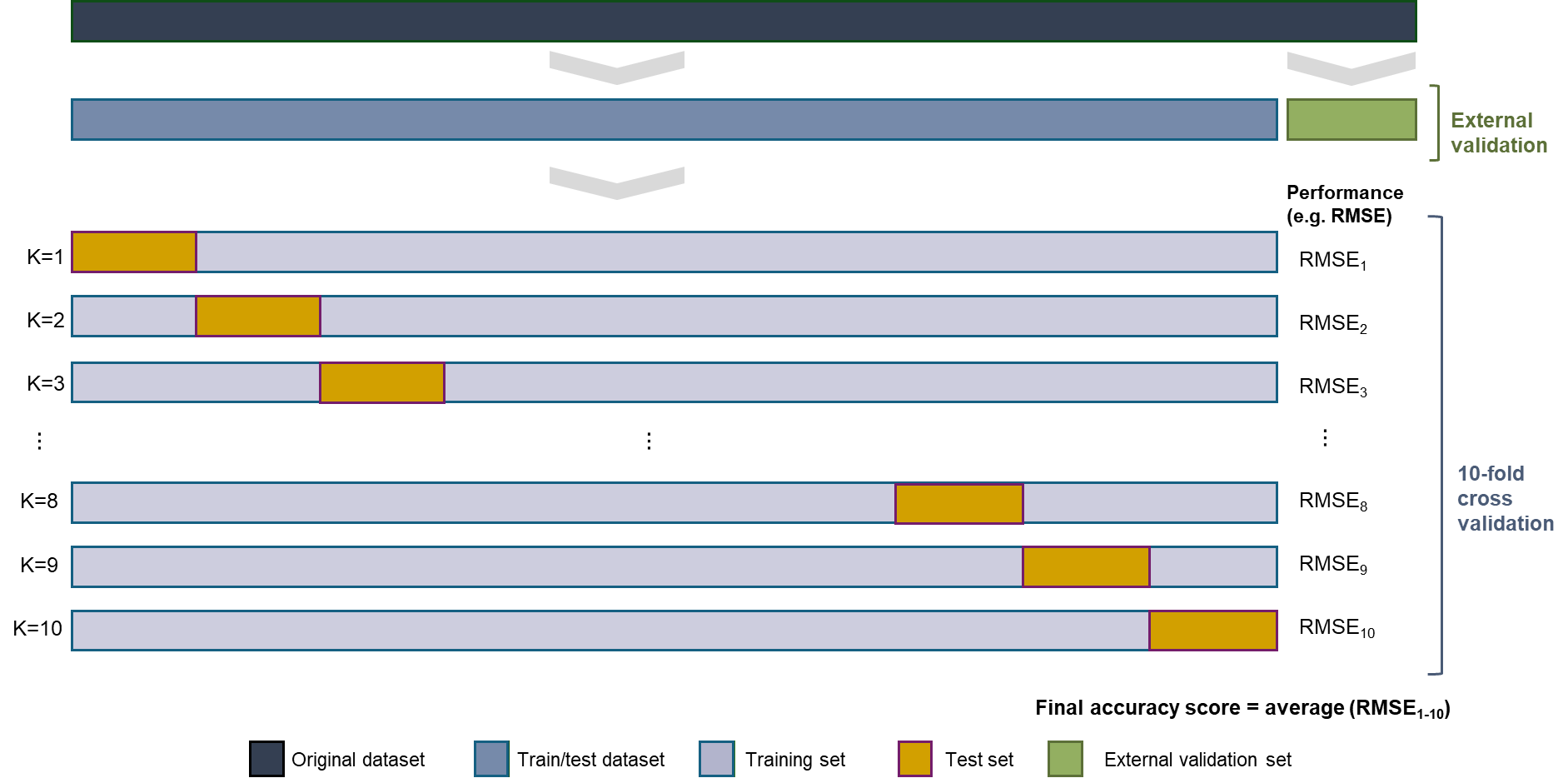


**Figure S5.** Diagram of the data split between training, testing and external validation.


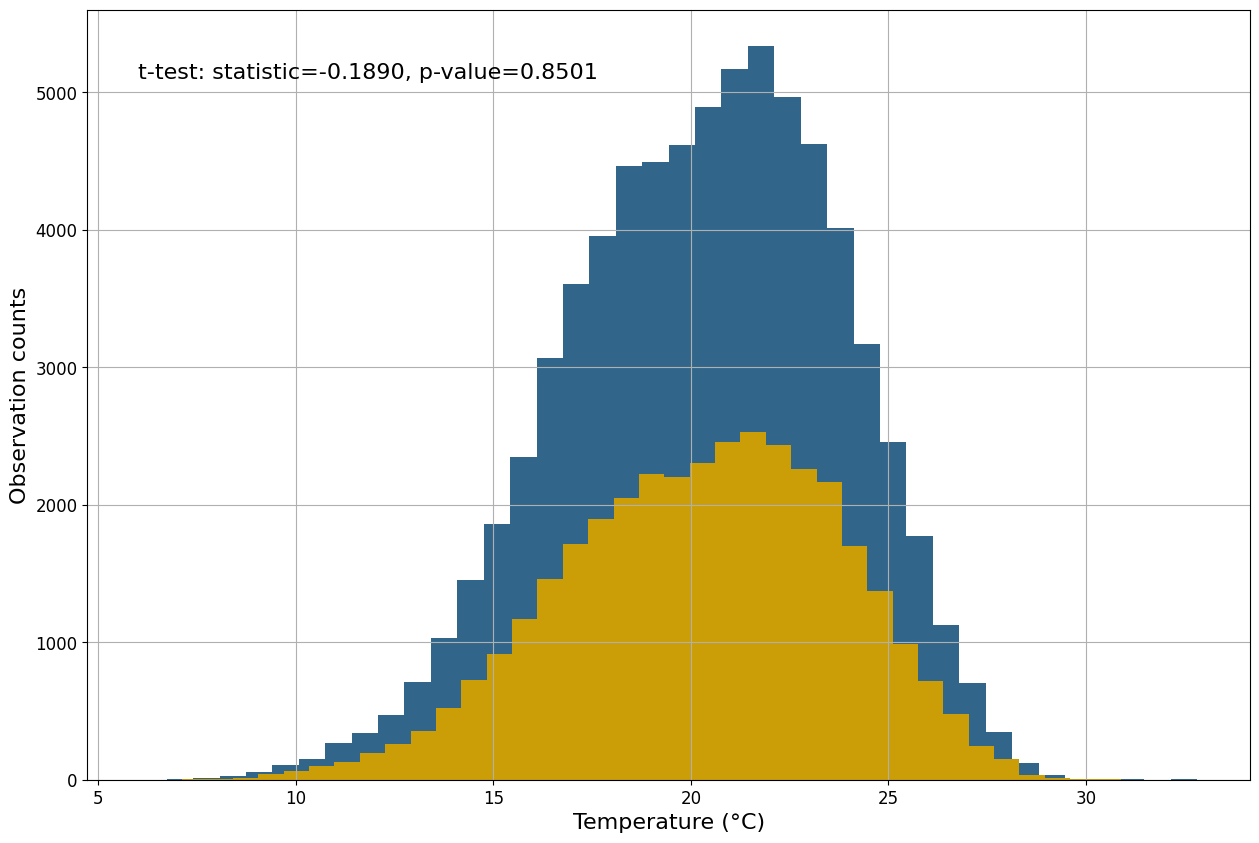


**Figure S6.** Density plot of daily temperature for hyperparameter tuning. In blue, the original dataset and in ochre, the stratified random sample used for the hyperparameter tuning strategy. t-test statistic and p-value provided.


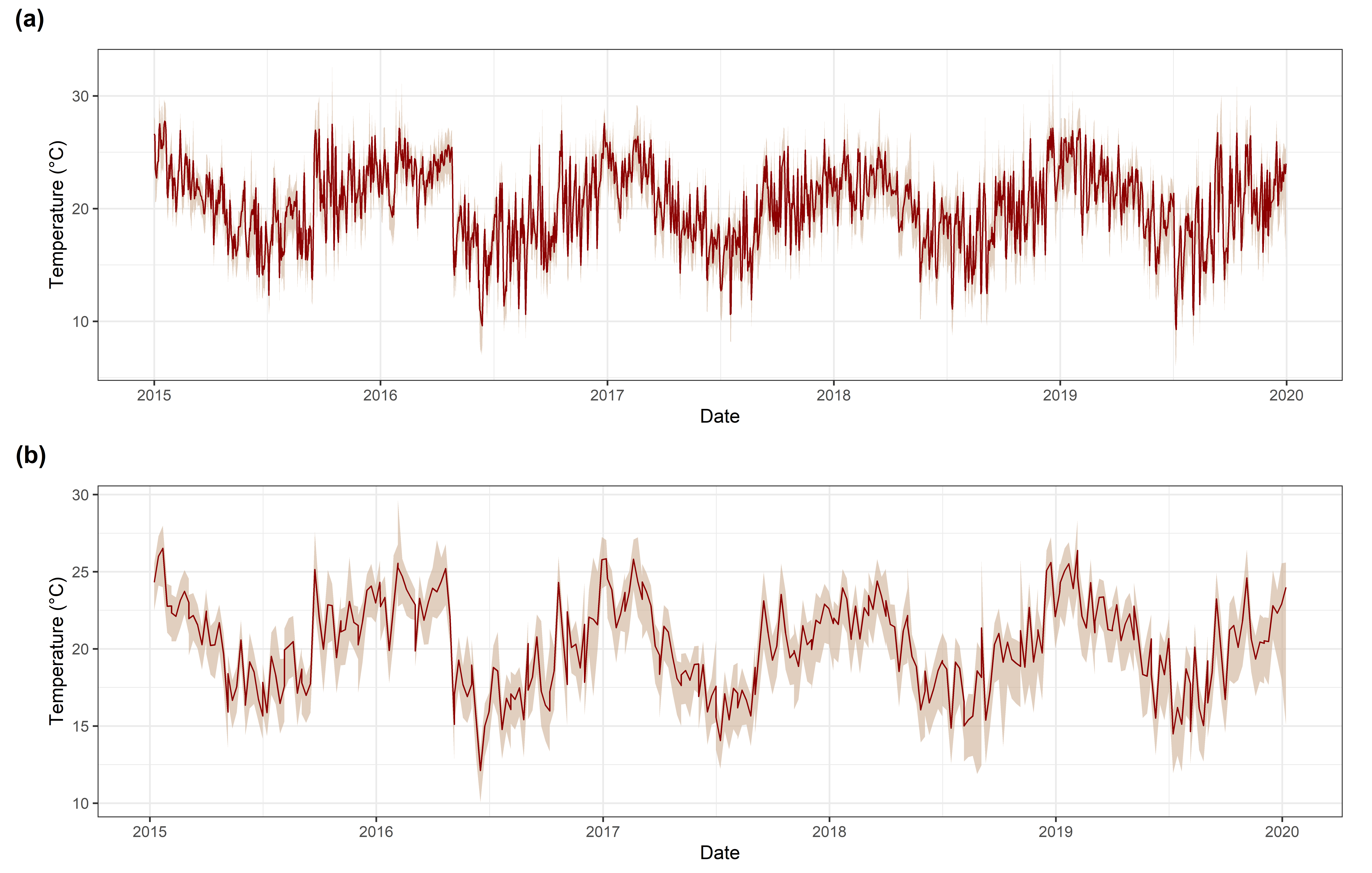


**Figure S7:** Daily and weekly and temperature from included meteorological stations. Average (solid line) and minimum/maximum (shaded) mean temperature by (a) day and (b) week between 2015 and 2020.


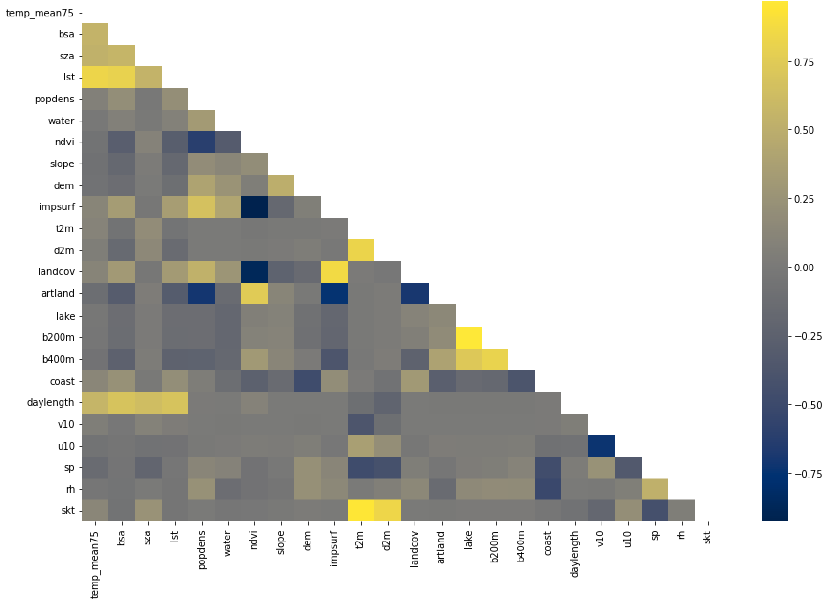


**Figure S8:** Pearson correlation matrix for target variable and all input features.

**
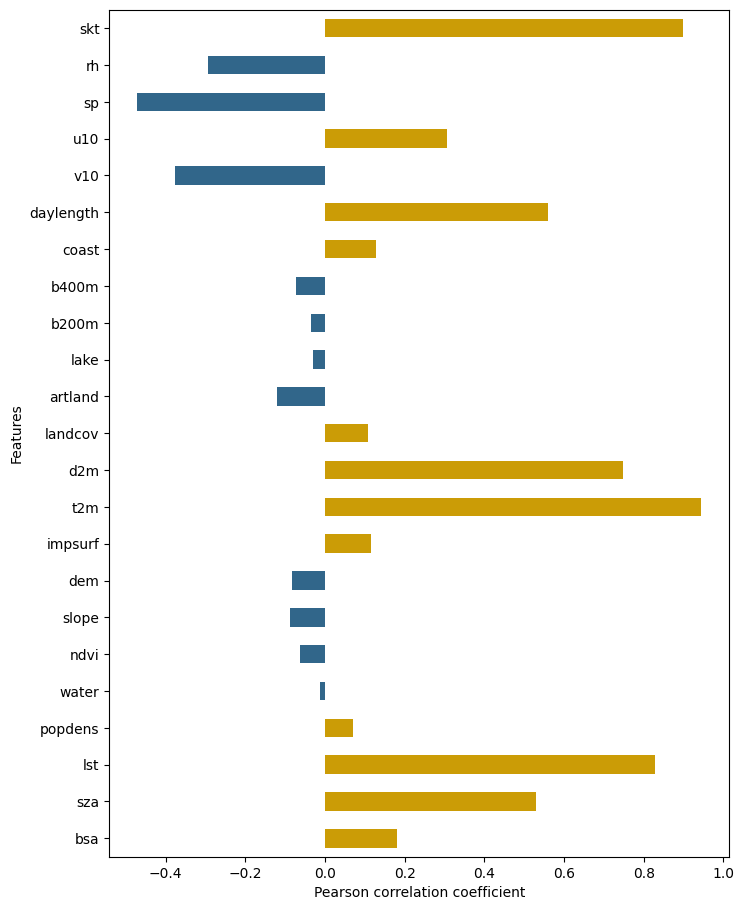
**

**Figure S9.** Bar plot showing the pairwise Pearson correlation coefficient between each predictor variable (y-axis) and the daily mean temperature as recorded by meteorological stations. Positive correlations in ochre, negative correlations in steel blue. Abbreviations: *rh*, relative humidity; *skt*, skin temperature; sp, surface pressure; *u10* and *v10*, horizontal and vertical wind component respectively; *daylength*, length of days; *coast*, distance to coast; *b400m* and *b200m*, buffer of 400 and 200m from perimeter of damns/lakes, respectively; *lake*, the perimeter of the damns/lakes; *artland* and *water*, distance to artificial land and inland water bodies per ESA-CCI-CL 2015 classification, respectively; *d2m* and *t2m*, dewpoint and surface temperature at 2m height; *dem*, elevation; *slope*, inclination or slope; *ndvi*, greenness index; *popdens*, population density; *lst*, land surface temperature; *sza*, solar zenith angle, and bsa, black sky albedo.


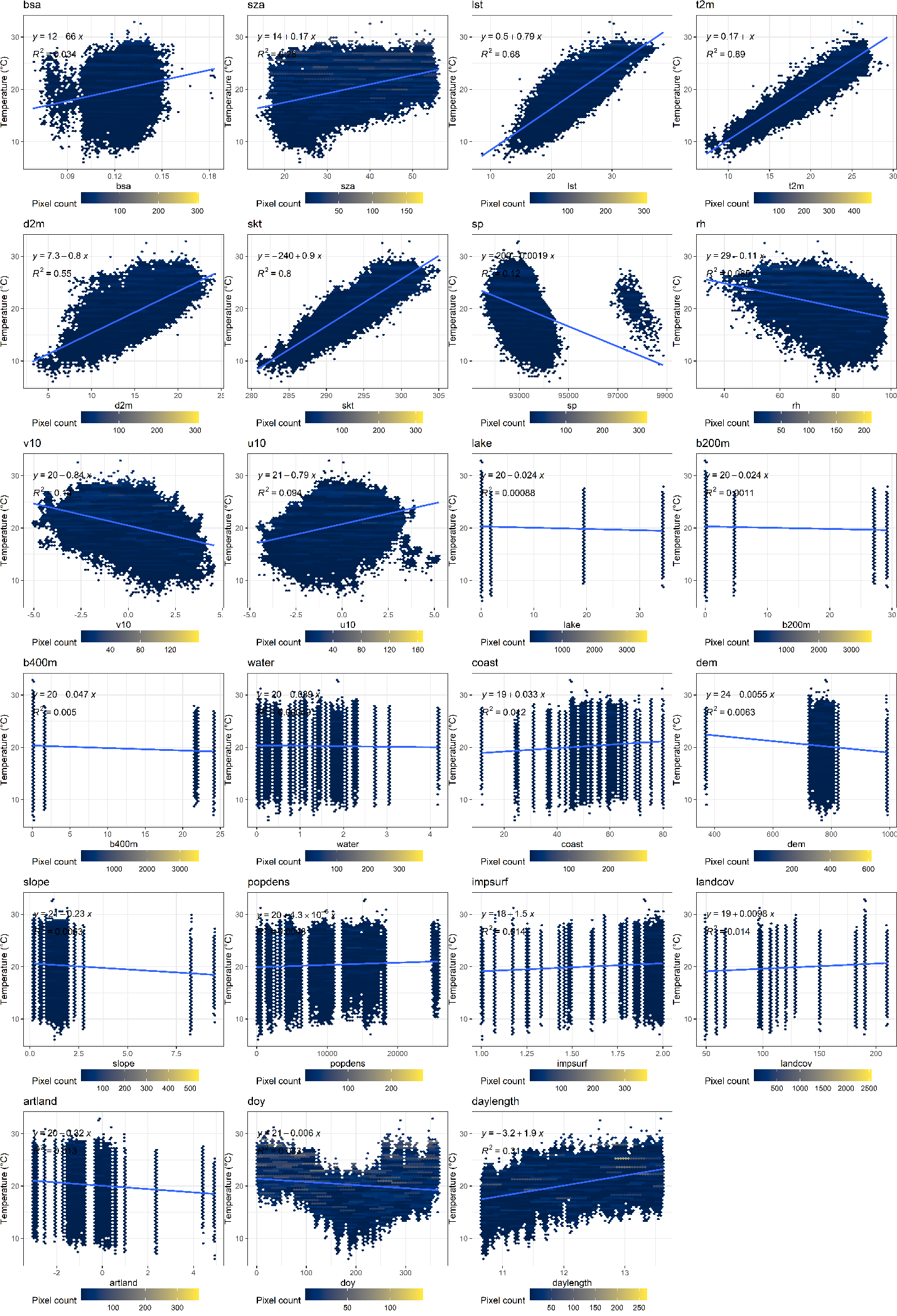


**Figure S10.** Scatter plots of all predictor variables against temperature. Linear regression equation and line (blue) provided with R^2^ value. Pixel count shown in a colour gradient.


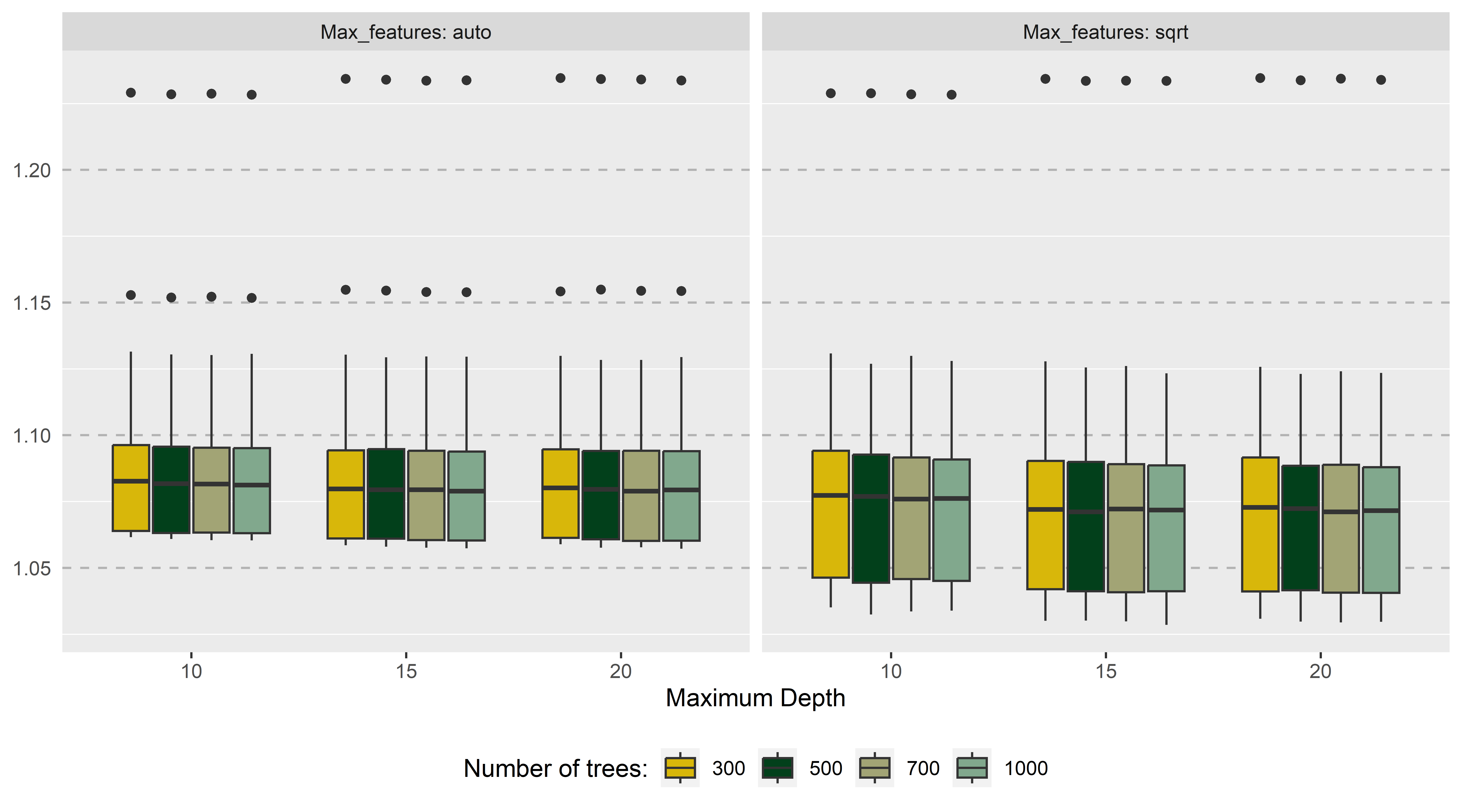


**Figure S11:** Hyper-parameter tuning. Model performance (RMSE, y-axis) by maximum feature (max_features, left and right hand side panels), maximum Depth (x-axis) and number of trees (colored)


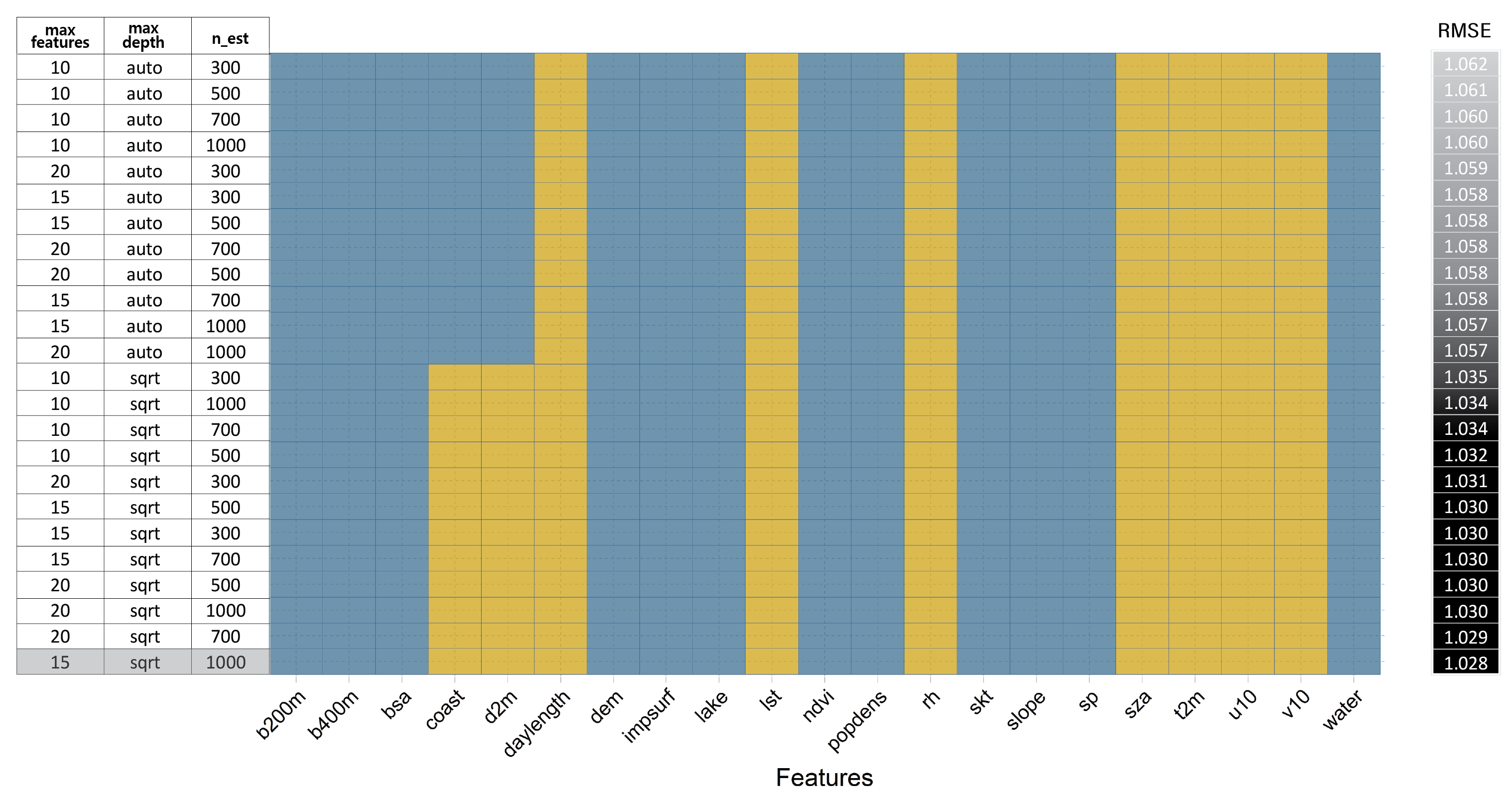


**Figure S12.** (Left) Heatmap showing the feature combination resulting in the best model performance for each hyper-parameter combination. In yellow, the features selected and in blue those excluded. (Right) RMSE for each model are shown in a gray scale, sorted from top (highest) to bottom (lowest) by performance. Performance assessed using spatial cross-validation.


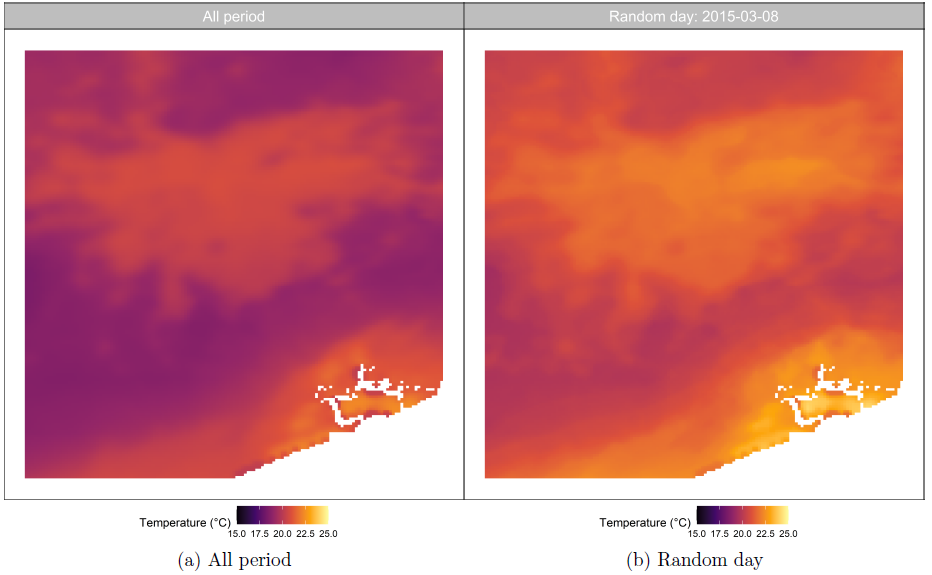


**Figure S13:** Average daily mean temperature for (a) the entire study period (2015 to 2019) and for (b) March 8th, 2015 (randomly selected day), as predicted by the RF algorithm.


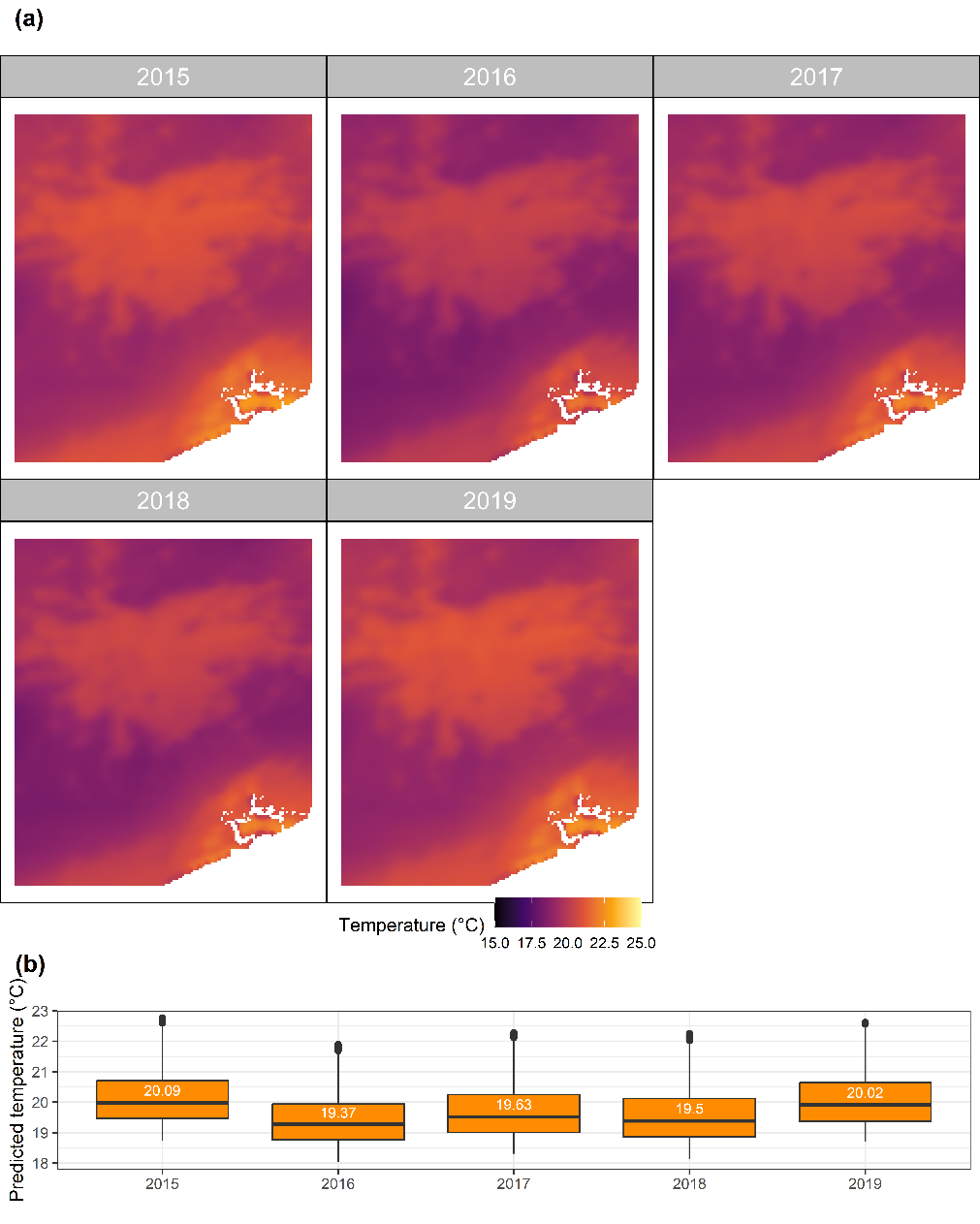


**Figure S14.** (a) Map of the daily mean temperature averaged by year across all study period (2015-2019) and (b) boxplot of the monthly temperature, as predicted by the RF algorithm.





**Figure S15.** RF temperature predictions by month. (a) Map of the daily mean temperature averaged by month across all study period (2015-2019) and (b) boxplot of the monthly temperature, as predicted by the RF algorithm.


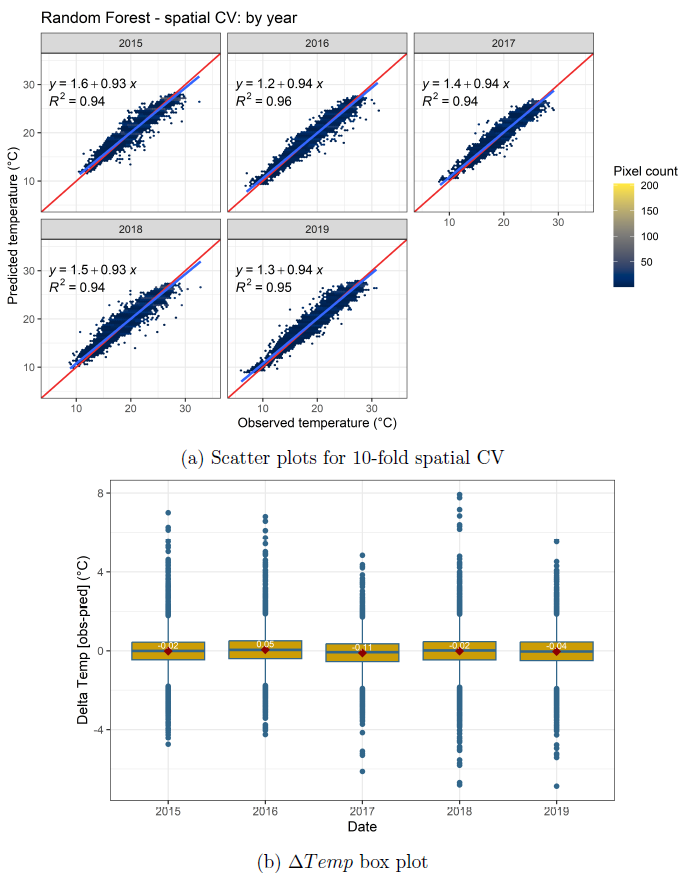


**Figure S16**. (a) Annual density scatter plots of predicted daily mean temperature versus daily mean temperature assessed through station-based CV. The red and blue lines represent the 1:1 line and the linear regression, respectively. The R^2^ and regression equation are shown for each plot. (b) Boxplot showing the difference between observed and predicted daily temperatures across all stations for each month in the study period (2015-2019). Red diamond indicates the mean; value label above.


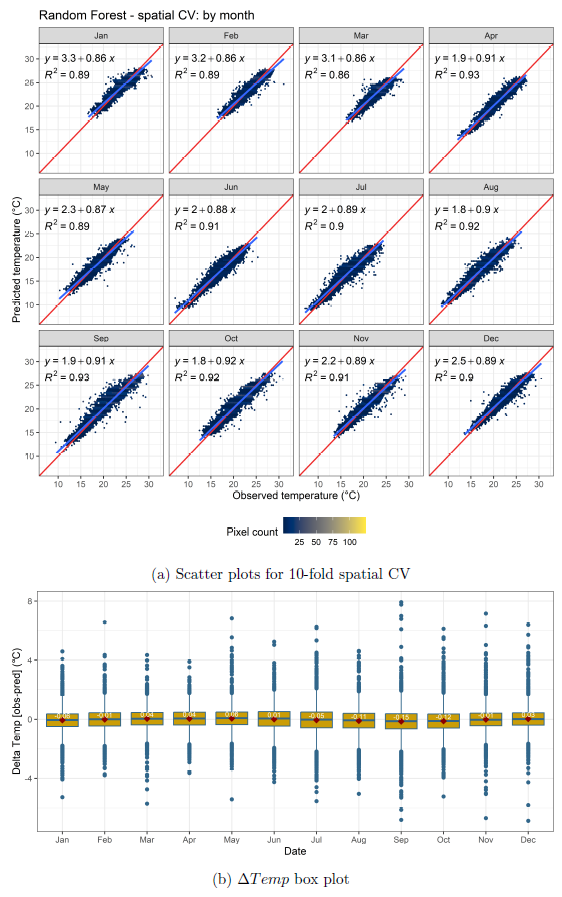


**Figure S17.** Monthly differences in model accuracy and ΔTemp.(a)Monthly density scatterplots of predicted daily mean temperature versus daily mean temperature assessed through station-based CV. The red and blue lines represent the 1:1 line and the linear regression, respectively. The R^2^ and regression equation are shown for each plot. (b) Boxplot showing the difference between observed and predicted daily temperatures across all stations for each year in the study period (2015-2019). Red diamonds indicate the mean; value label above.


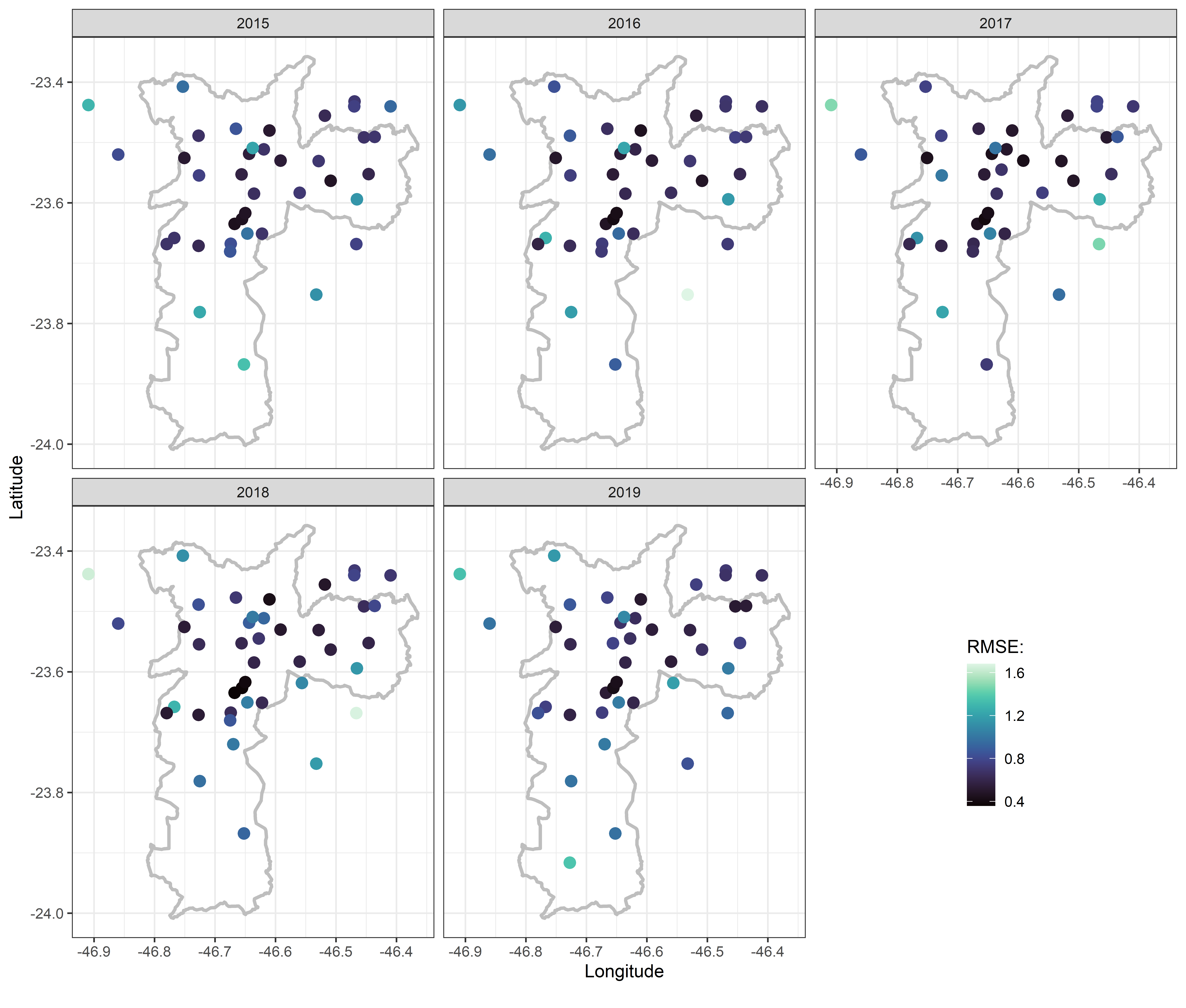


**Figure S18.**  Average RMSE (^◦^C) per station by year, assessed through station-based CV.


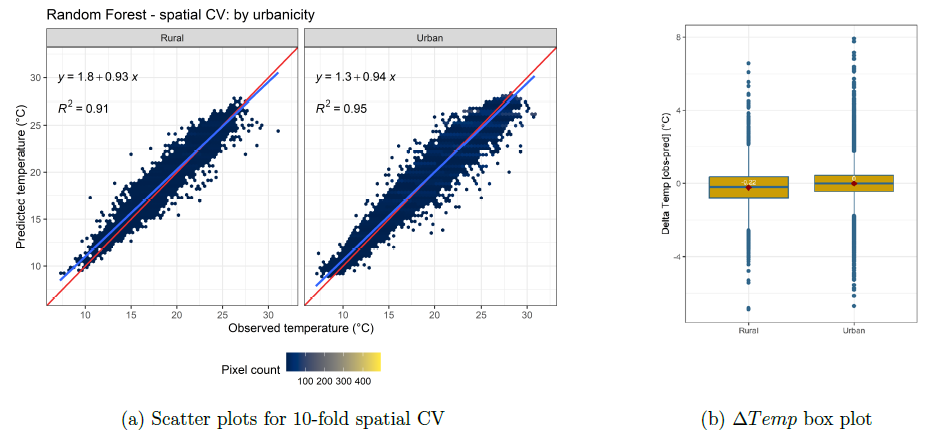


**Figure S19.** Rural/urban differences in model accuracy and ΔTemp. (a) Density scatter plots of predicted daily mean temperature versus daily mean temperature for stations classified as urban vs. rural, assessed through station-based CV. The red and blue lines represent the 1:1 line and the linear regression, respectively. The R^2^ and regression equation are shown for each plot. (b) Boxplot showing the difference between observed and predicted daily temperatures (ΔTemp) across all stations by urban/rural classification across all study period (2015-2019). The red diamond indicates the mean; value label above.


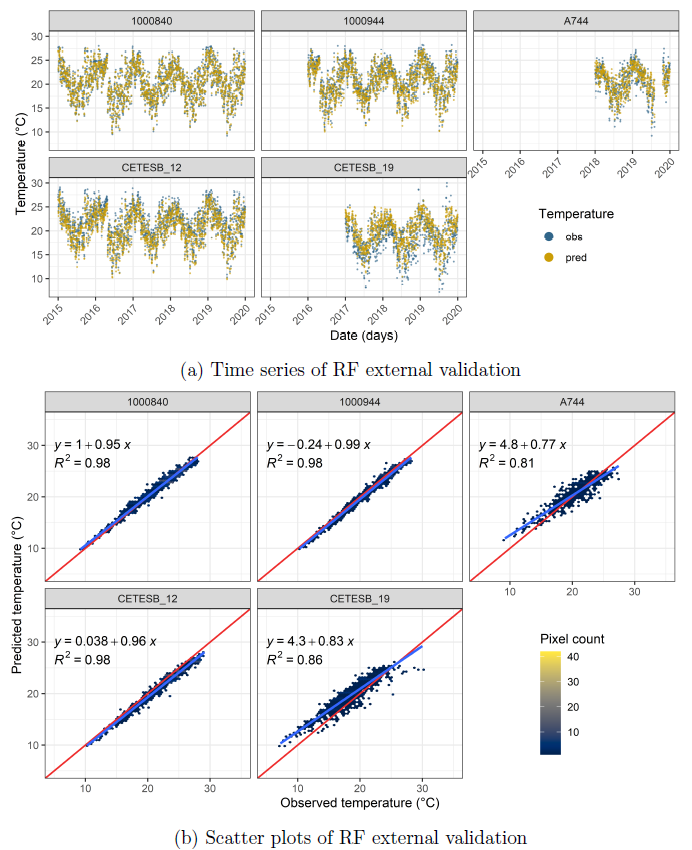


**Figure S20:** Time series and scatter plot of RF external validation. (a) Time series plots of

predicted daily mean temperature (ochre) versus daily mean temperature from the five meteorological stations (hold-out dataset, blue). (b) Density scatter plots of predicted daily mean temperature versus daily mean temperature from the five meteorological stations (hold-out dataset). The red and blue lines represent the 1:1 line and the linear regression, respectively. The R^2^ and regression equation are shown for each plot.


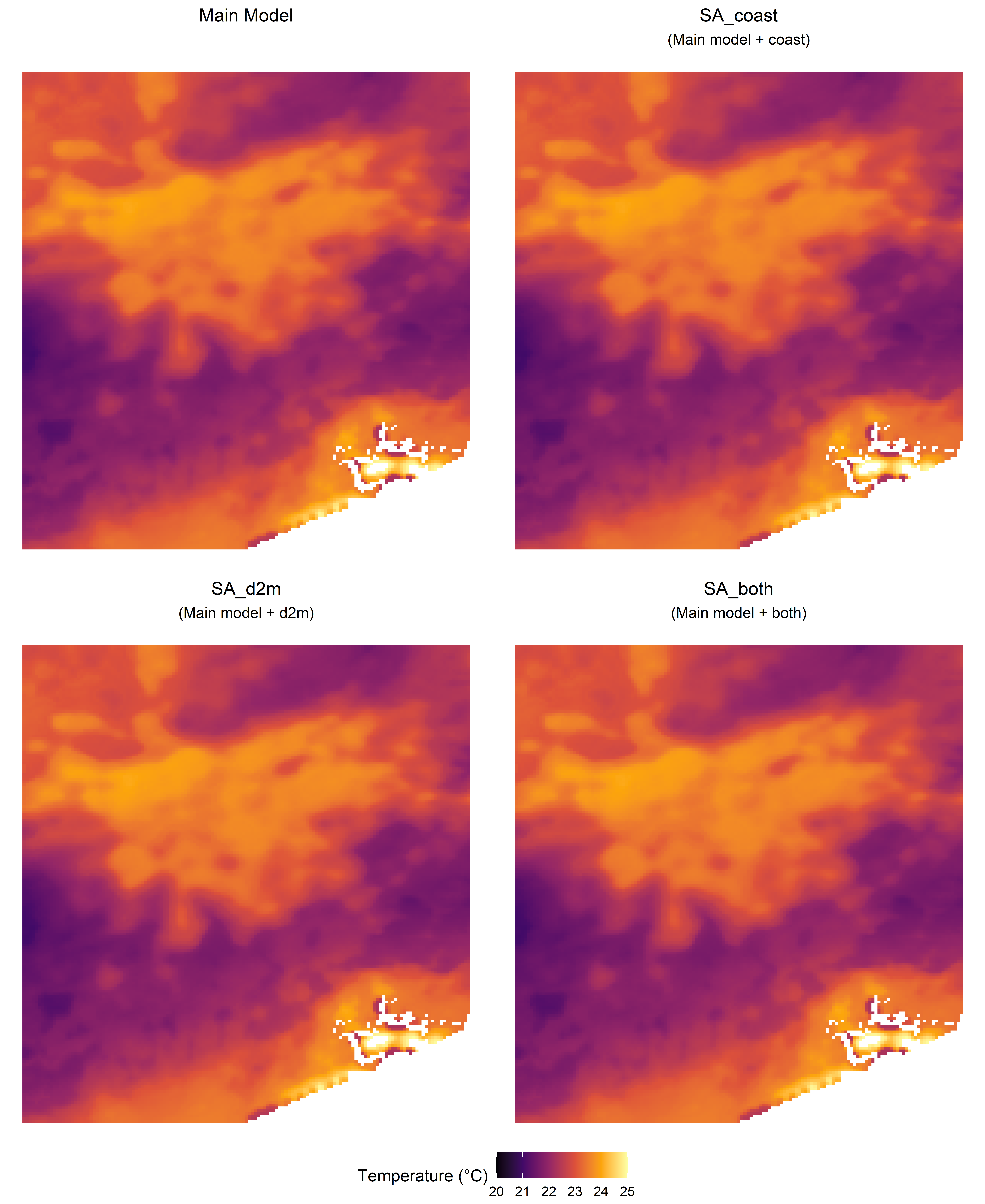


**Figure S21.** Map of predicted temperature overall, by sensitivity analyses. Map of the

daily mean temperature averaged across all study period as predicted by each sensitivity analyses.

(a)

(b)


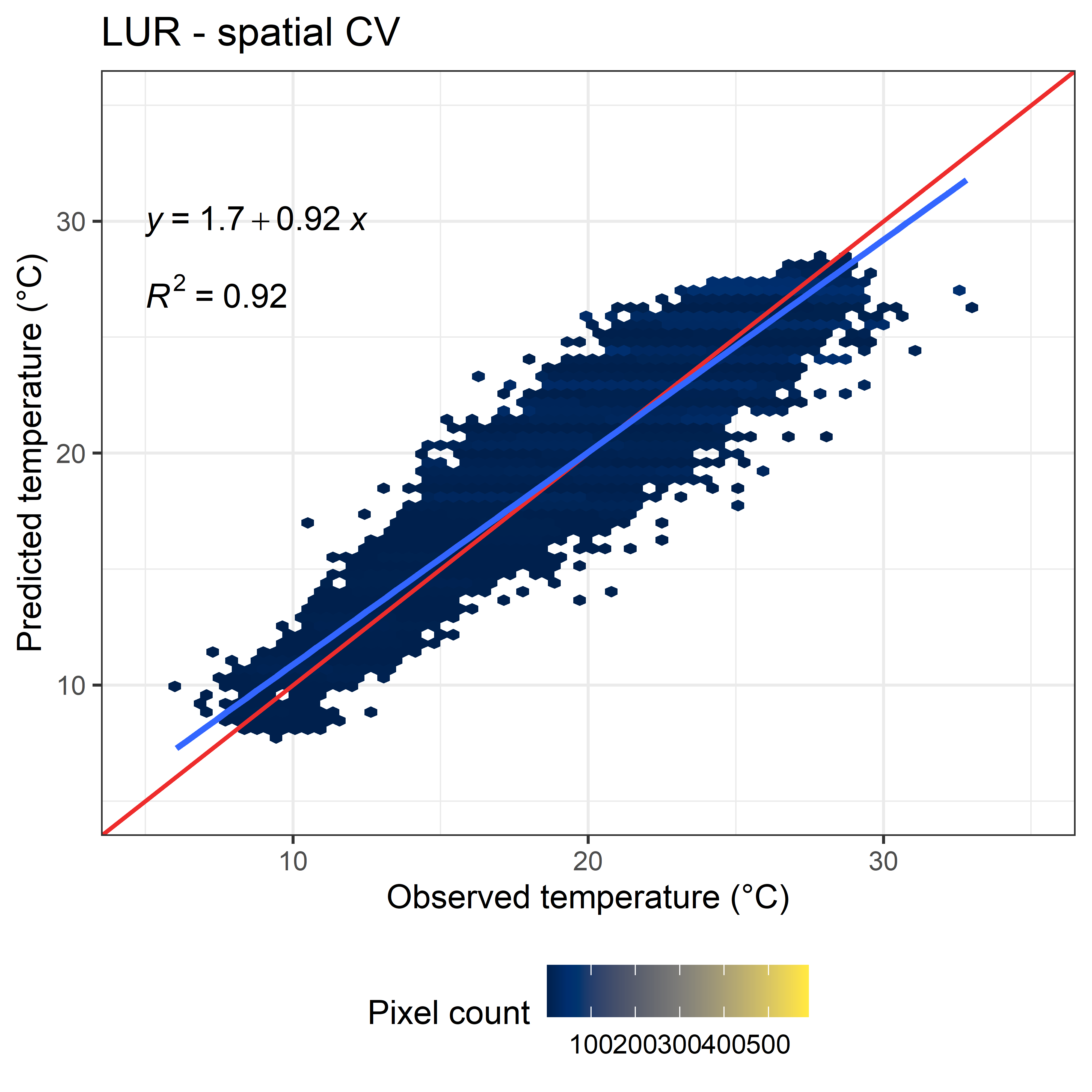


1. Scatter plot of MLR station-based CV


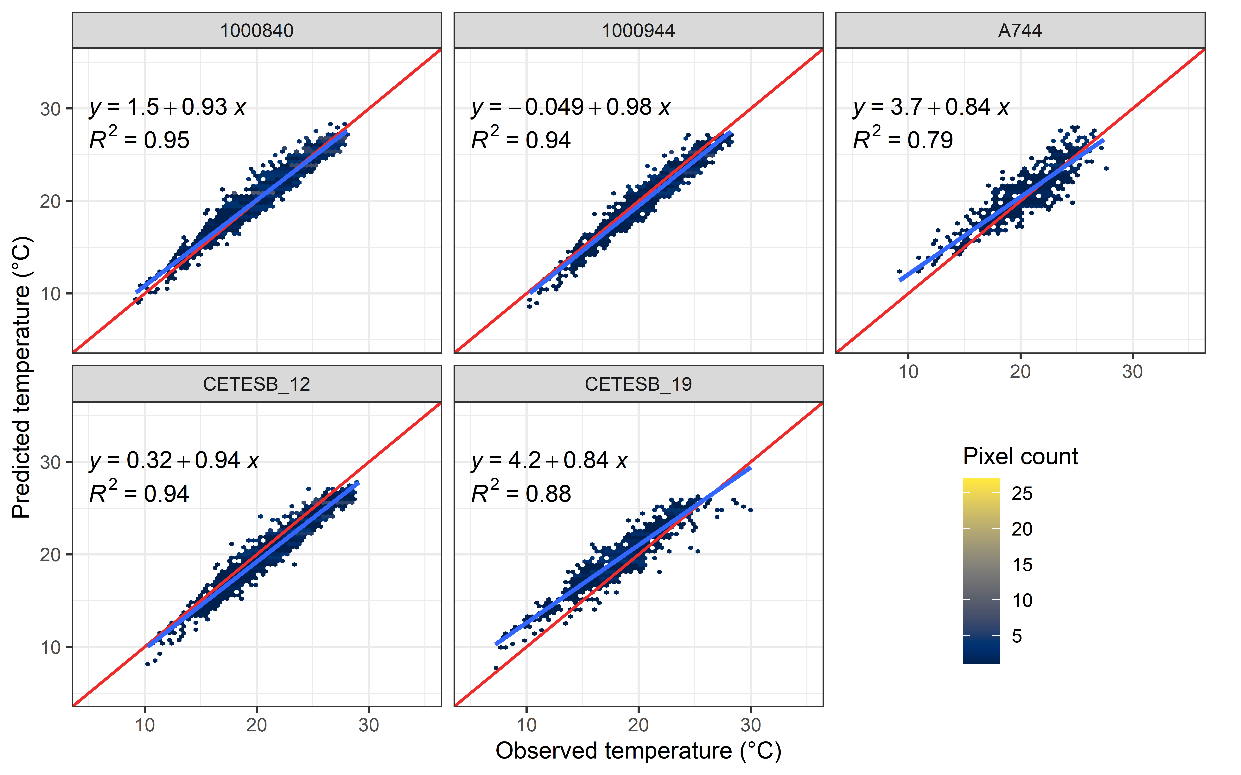


1. Scatter plot of MLR external validation

**Figure S22**. Density scatter plots of predicted daily mean temperature versus daily mean temperature using (a) 10-fold station-based CV, and for (b) the five hold-out meteorological stations (external validation). Red and blue lines represent the 1:1 line and the linear regression, respectively. R^2^ and regression equation shown at each plot.

(a)

(b)

(c)


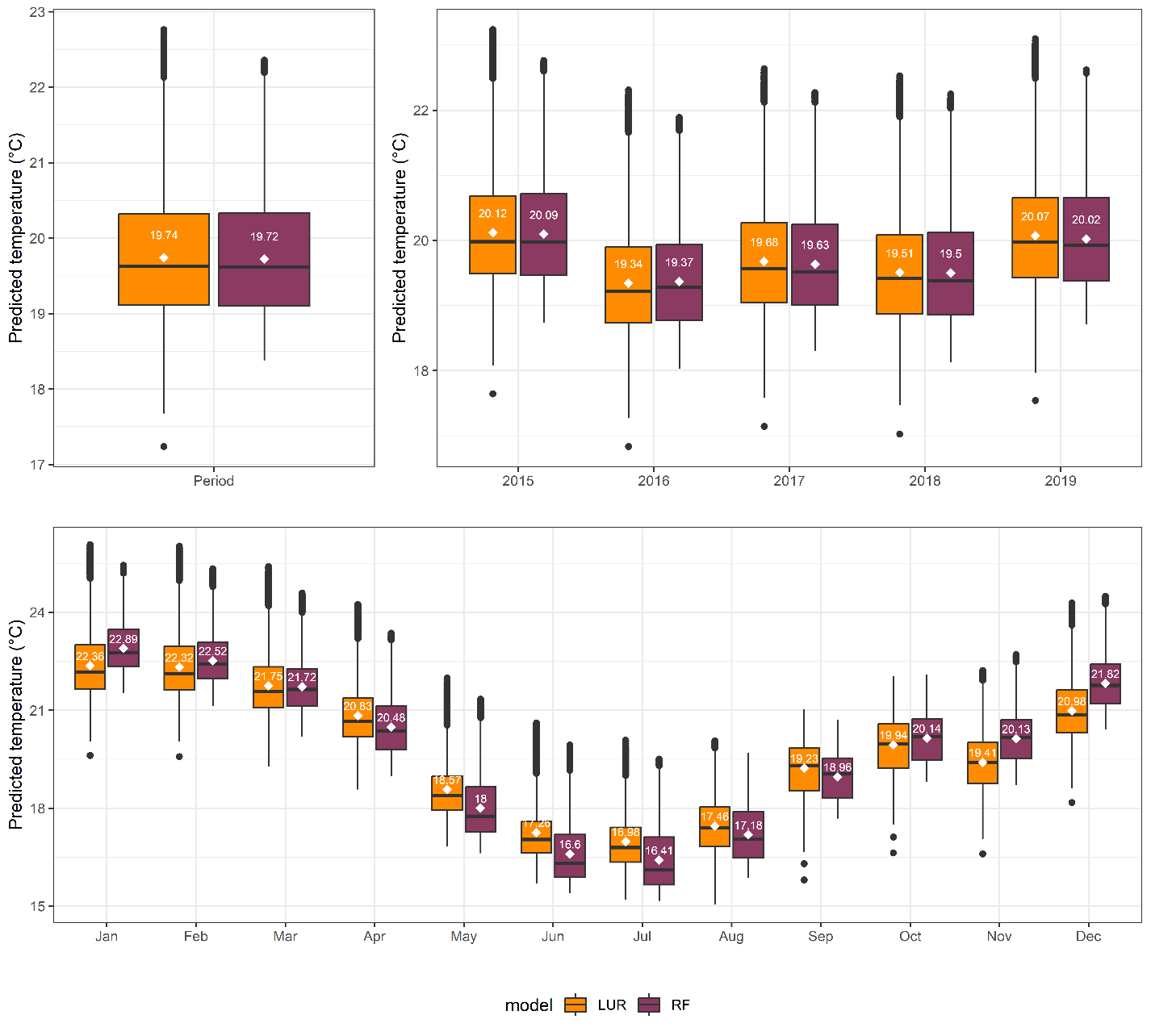


MLR

**Figure S23.** Boxplot of the daily mean temperature predicted by the RF and MLR models (a) over the entire study period, (b) by year, and (c) by month. Lower and upper box boundaries indicate the 25th and 75th percentiles, respectively, line inside box median, lower and upper error lines 10^th^ and 90th percentiles, respectively, filled circles data falling outside 10th and 90th percentiles.


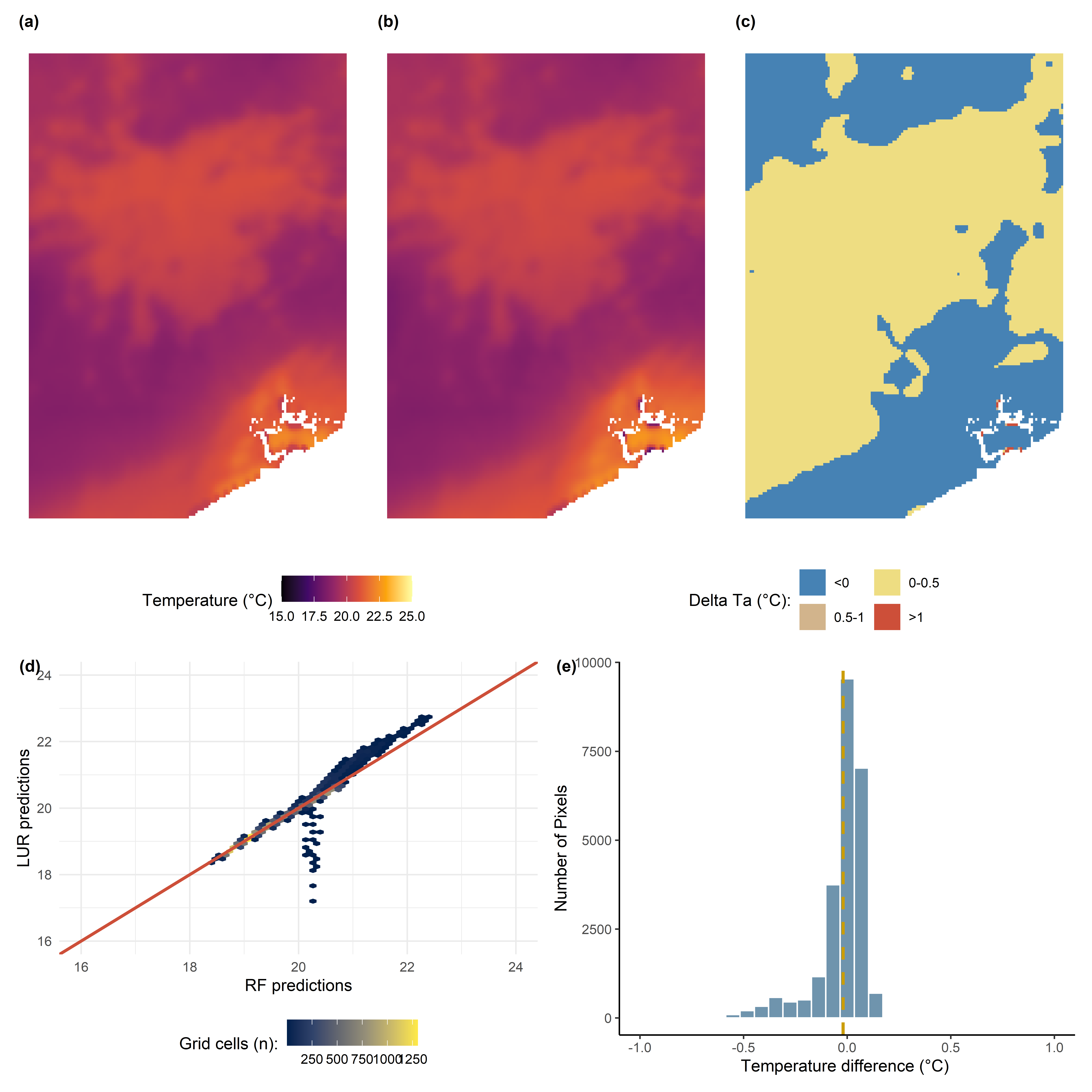


**Figure S24.** Visual comparison of the spatial distribution of the average daily mean temperature predicted by (a) RF, (b) MLR and (c) the delta temperature between RF-MLR model predictions over the entire study period (2015-2019).
